# Lymph node-targeted, mKRAS-specific amphiphile vaccine in pancreatic and colorectal cancer: phase 1 AMPLIFY-201 trial final results

**DOI:** 10.1038/s41591-025-03876-4

**Published:** 2025-08-11

**Authors:** Zev A. Wainberg, Colin D. Weekes, Muhammad Furqan, Pashtoon M. Kasi, Craig E. Devoe, Alexis D. Leal, Vincent Chung, James R. Perry, Thian Kheoh, Lisa K. McNeil, Esther Welkowsky, Peter C. DeMuth, Christopher M. Haqq, Shubham Pant, Eileen M. O’Reilly

**Affiliations:** 1 University of California, Los Angeles, Los Angeles, CA USA; 2 Massachusetts General Hospital, Boston, MA USA; 3 University of Iowa, Iowa City, IA USA; 4 Northwell Health, Lake Success, NY USA; 5 University of Colorado School of Medicine, Aurora, CO USA; 6 City of Hope, Duarte, CA USA; 7 Elicio Therapeutics, Boston, MA USA; 8 University of Texas MD Anderson Cancer Center, Houston, TX USA; 9 Memorial Sloan Kettering Cancer Center, New York City, NY USA

**Keywords:** Cancer immunotherapy, Pancreatic cancer, Colon cancer

## Abstract

Cellular immunity, mediated by tumor antigen-specific CD4^+^ and CD8^+^ T cells, has a critical role in the success of cancer immunotherapy by targeting intracellular driver and passenger tumor mutations. We present the final results of the phase 1 AMPLIFY-201 trial, in which patients who completed standard locoregional treatment, with minimal residual mKRAS disease (*n* = 25, 20 pancreatic cancer and 5 colorectal cancer), received monotherapy vaccination with lymph node-targeting ELI-002 2P, including mutant KRAS (mKRAS) amphiphile-peptide antigens (G12D, G12R) and amphiphile-adjuvant CpG-7909. At a median follow-up of 19.7 months, efficacy correlated with mKRAS-specific T cell responses above or below a threshold 9.17-fold increase over baseline, with median radiographic relapse-free survival not reached, versus 3.02 months (hazard ratio (HR) = 0.12, *P* = 0.0002) and median overall survival not reached versus 15.98 months (HR = 0.23, *P* = 0.0099). Seventy-one percent of evaluable patients induced both CD4^+^ and CD8^+^ subsets, with sustained immunogenicity. Following ELI-002 2P treatment, antigen spreading was observed in 67% of patients, with increased T cells reactive to personalized, tumor antigens absent from the ELI-002 2P vaccine. Therefore, lymph node-targeting amphiphile vaccination induces persistent T cell responses targeting oncogenic driver KRAS mutations, alongside personalized, tumor antigen-specific T cells, which may correlate to clinical outcomes in pancreatic and colorectal cancer. ClinicalTrials.gov registration: NCT04853017.

## Abstract (web summary)

In the final analysis of a phase 1 trial evaluating a lymph node-targeting mutant KRAS peptide vaccine combined with CpG in the adjuvant setting for patients with KRAS mutant pancreatic or colorectal cancer, median relapse-free survival and overall survival were not reached for patients with robust antigen-specific T cell responses, and these responses were durable with evidence of antigen spreading.

## Main

Tumor-promoting driver mutations in KRAS occur in approximately 20–25% of human tumors, including colorectal cancer (CRC) (50%) and pancreatic ductal adenocarcinoma (PDAC) (93%). Despite curative intent, relapses are common following standard locoregional therapy, particularly for resectable PDAC. Subsequent elevated ctDNA or serum tumor antigen defines a biomarker-relapsed, minimal residual disease (MRD^+^) patient group at high risk for radiographic progression^1,2^. Further treatment following disease relapse/recurrence is primarily palliative and noncurative in intent (5-year survival = 23.3%^3^). Mutations in KRAS are attractive public neoantigens for immunotherapy due to their prevalence, truncal status and essential driver function. Growing evidence of mKRAS recognition by diverse human HLA alleles^4–6^ suggests that many patients could benefit from an effective off-the-shelf mKRAS-specific therapeutic vaccine. Recent analysis found T cells specific to G12D and/or G12V in 20/20 (100%) of healthy donors evaluated across a diverse HLA background, suggesting that the majority of patients include mKRAS-specific TCRs within their immune repertoire^6^. However, vaccination with conventional immunogens, including relatively small (<20 kDa) peptide antigens and molecular adjuvants, results in poor accumulation in lymph nodes where uptake by antigen-presenting cells programs adaptive immunity^7^. Conversely, chemical modification with albumin-binding lipid moieties facilitates delivery of amphiphile vaccines from peripheral injection sites to lymph nodes via size-dependent lymphatic transport by endogenous albumin (~65 kDa), resulting in improved antigen-specific T cell responses^8^.

**Extended Data Fig. 1:**
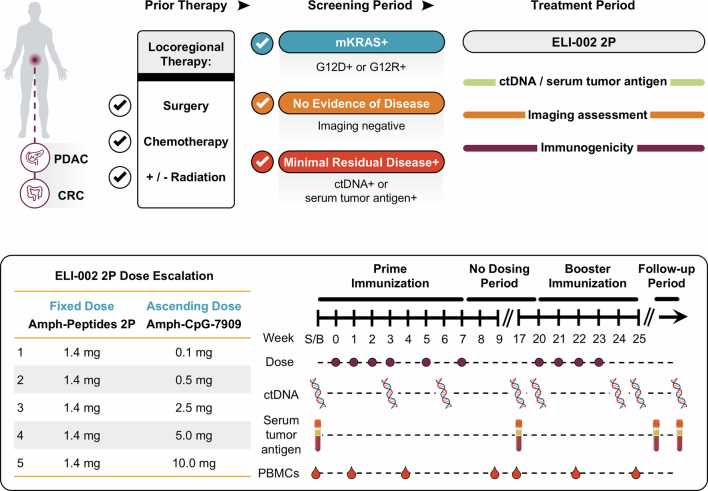
Study schema. Treatment with ELI-002 2P was conducted in a priming immunization period (B: baseline, S: Screening; week 0–week 7) and subsequent boosting immunization period (week 20–23) for CRC or PDAC patients exhibiting tumor expression of mKRAS G12D or G12R following completion of locoregional therapy, radiological confirmation of no evidence of disease and detection of either ctDNA or serum tumor biomarker indicating positivity for minimal residual disease (MRD). The figure was created with BioRender.com.

We previously reported the safety, immunogenicity and preliminary antitumor activity observed in a dose escalation phase 1 trial^9^. Patients received ELI-002 2P, including lymph node-targeted amphiphile mKRAS G12D-specific and G12R-specific, 18-mer peptide antigens, designed to be processed into HLA class I and HLA class II epitopes. These immunizing peptide antigens were co-administered with an amphiphile-adjuvant CpG-7909 in 25 patients with surgically resected stage I-IV PDAC (*n* = 20) and CRC (*n* = 5) who had no evidence of disease on imaging but had detectable MRD^+^ (ctDNA positive in *n* = 13/25, CA19-9 and/or carcinoembryonic antigen (CEA) criteria in *n* = 7/25, or both *n* = 5/25; Extended Data Fig. 1 and Extended Data Table 1). Preliminary results at the initial time of data cutoff (6 September 2023; median cohort follow-up time 8.5 months) showed that monotherapy treatment with ELI-002 2P-induced potent mKRAS-directed T cell responses in 21/25 (84%) of the participants, including both CD4^+^ and CD8^+^ T cells in 59%. T cell responses above the median 12.75-fold increase from baseline significantly correlated with improved tumor biomarker response and relapse-free survival (RFS)^9^. Herein we update post hoc analyses of immunogenicity and clinical outcomes with more than double the follow-up time to demonstrate that ELI-002 2P-induced T cell responses are potent, sustained and continue to correlate with freedom from relapse and death. Immune responses included sustained mKRAS-specific CD4^+^ and CD8^+^ T cell subsets exhibiting favorable effector cytokine function, cytotoxic markers and memory phenotype, as well as antigen spreading to personalized tumor antigens beyond the immunizing antigens.

**Fig. 1:**
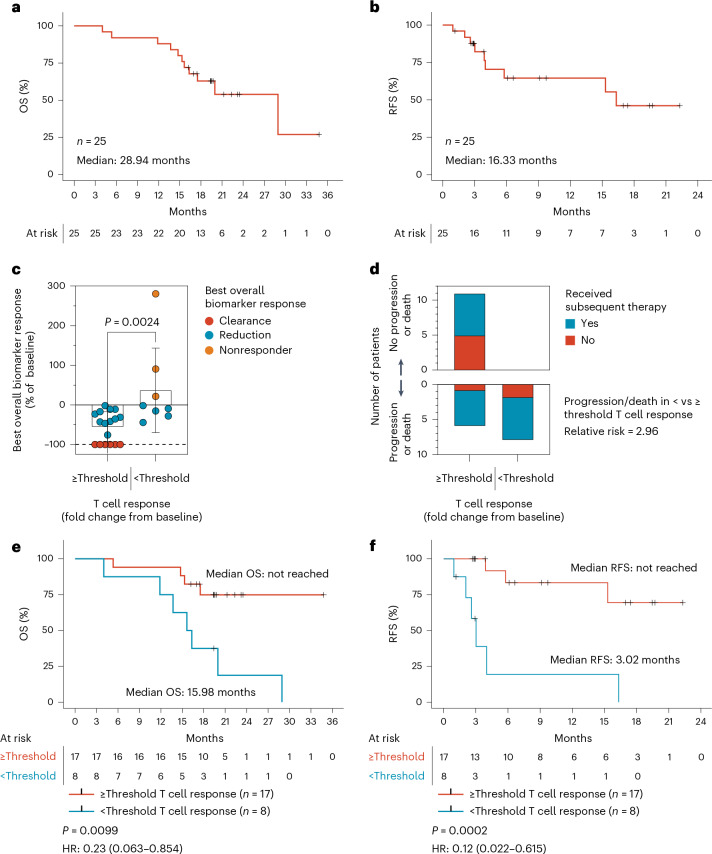
mKRAS-specific T cell response correlates with tumor biomarker response and long-term clinical outcomes. **a**,**b**, OS defined as the time from first vaccine dose until death from any cause (**a**) and radiographic RFS defined as the time from first vaccine dose until confirmed radiographic progression according to iRECIST criteria or death in 25 patients (**b**). If subsequent therapy was given, RFS was censored at the date of the most recent radiographic scan before the date of subsequent therapy, the data cutoff or the date of death. **c**–**f**, Patients were stratified by mKRAS-specific T cell response fold change from baseline at the threshold of 9.17. Patients at or above versus below threshold were compared for **c**, best overall biomarker response reported as percentage relative to the baseline value, error bars are mean ± s.d., *P* value calculated by two-tailed Mann–Whitney test, number of patients with or without iRECIST progression or death (**d**), OS (**e**) and radiographic RFS (**f**). *n* indicates individual patients. HR indicates hazard ratio with 95% CI. *P* values calculated using two-tailed log-rank test. Source data

**Extended Data Fig. 2:**
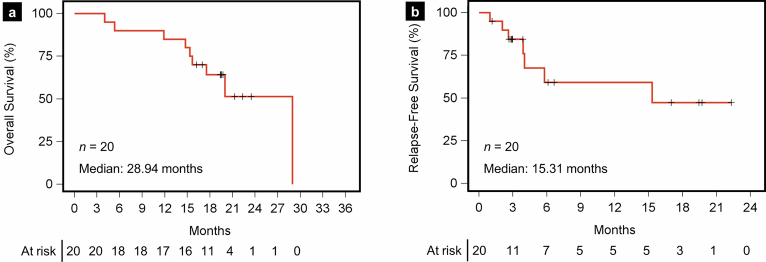
Clinical outcomes for PDAC subset. **a**,**b**, OS (**a**) and radiographic RFS (**b**) from study start in n = 20 PDAC patients. Source data

At a median follow-up of 19.7 months for the cohort (data cutoff: 24 September 2024), no new safety signals were identified in the primary endpoint assessment. The exploratory endpoints of radiographic RFS and overall survival (OS) were reassessed. At extended follow-up, the *n* = 25 cohort median OS was 28.94 months (Fig. 1a). The median RFS was maintained at 16.33 months (Fig. 1b). Analysis of the PDAC subset indicated that mRFS (15.31 months) and mOS (28.94 months) were similar to those observed for the whole cohort (Extended Data Fig. 2). Study visits were concluded for AMPLIFY-201 in August 2024.

**Extended Data Fig. 3:**
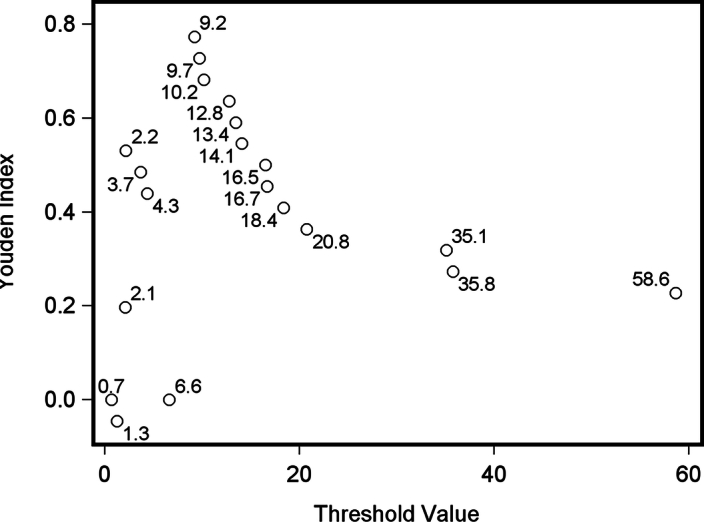
ROC analysis. Scatter plot of T cell fold change threshold values by decreasing order of Youden Index. A larger Youden Index indicates a more optimal cutoff value^22^. T cell fold change threshold values were rounded to the nearest tenth decimal. Source data

**Extended Data Fig. 4:**
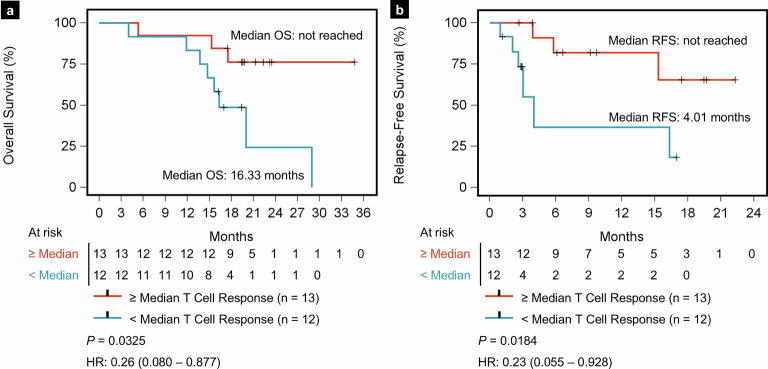
rRFS and OS supervised by prior median 12.75 T cell fold change. **a**, OS defined as the time from first vaccine dose until death from any cause and **b**, radiographic RFS defined as the time from first vaccine dose until confirmed radiographic progression according to iRECIST criteria or death in n = 25 patients. If subsequent therapy was given, radiographic RFS was censored at the date of the most recent radiographic scan before the date of subsequent therapy, the data cutoff or the date of death. Patients were stratified by mKRAS-specific T cell response fold change from baseline at the previously determined median of 12.75. Patients at or above versus below median were compared for **a** (OS) and **b** (radiographic RFS). n indicates individual patients. HR indicates hazard ratio with 95% CI. P values calculated using two-tailed log-rank test. Source data

To assess the potential impact of mKRAS-specific T cell responses on clinical outcomes (OS and radiographic RFS), an exploratory receiver-operating-characteristics (ROC) analysis was conducted^10^. While prior supervision was performed empirically using the median^9^, we identified a T cell fold-change threshold of 9.17 that optimally separated patients with better (*n* = 17, 68%) and worse (*n* = 8, 32%) outcomes, as determined by ROC analysis (Extended Data Fig. 3). As observed previously, the strength of ELI-002 2P-induced mKRAS-specific T cell response was correlated to tumor biomarker response (Fig. 1c), with patients exhibiting T cell response fold change above the 9.17 threshold universally achieving biomarker reductions, including six of six (100%) patients achieving complete ctDNA clearance. Presence or absence of radiographic progression or death (radiographic RFS) was also significantly associated with T cell response (Fig. 1d and Extended Data Table 1)—among the 17 patients with T cell responses above the 9.17 threshold, 11 (65%) were free from radiographic progression, including 5 who remained free of relapse and did not receive any subsequent therapy following ELI-002 2P; 6 (35%) patients received subsequent chemotherapy following tumor biomarker increase yet remained free from disease progression throughout follow-up. As three of six patients who remained free from radiographic progression received subsequent therapy, including immune checkpoint inhibition, following biomarker progression, it will be essential to evaluate these combinations prospectively in future trials. Although subsequent chemotherapy has the potential to negatively impact expanding tumor-specific T cells, prior use of sequential vaccination and chemotherapy regimens has shown that vaccine-induced T cell responses can be maintained following cytotoxic therapy^11,12^. The current data suggest that anti-mKRAS T cell induction may synergize with subsequent treatment to enable unexpectedly positive outcomes. In contrast to patients with T cell responses above the 9.17 threshold, radiographic progression was observed for all eight patients below the T cell threshold, and seven of eight (88%) died. This is consistent with an approximate threefold increase in risk of radiographic progression/death in the below threshold group (relative risk = 2.96), further suggesting that sufficient cellular immunity to driver mKRAS oncogenes may confer durable benefit. Moreover, the median OS of patients with above-threshold T cell responses was not reached, compared to 15.98 months (Fig. 1e; hazard ratio (HR) = 0.23, *P* = 0.0099). Finally, the median RFS was not reached compared to 3.02 months for patients with T cell fold change above or below the threshold (Fig. 1f; HR = 0.12, *P* = 0.0002). This was consistent with updated analysis of radiographic RFS and OS supervised by the prior 12.75 median T cell fold change (Extended Data Fig. 4). The high proportion of patients with above-threshold T cell responses who remained without radiographic evidence of disease following subsequent therapy for biochemical progression (6/11, 55%) suggests that vaccination with ELI-002 or early treatment at the time of biochemical progression may be important strategies to evaluate in future prospective trials.

**Fig. 2:**
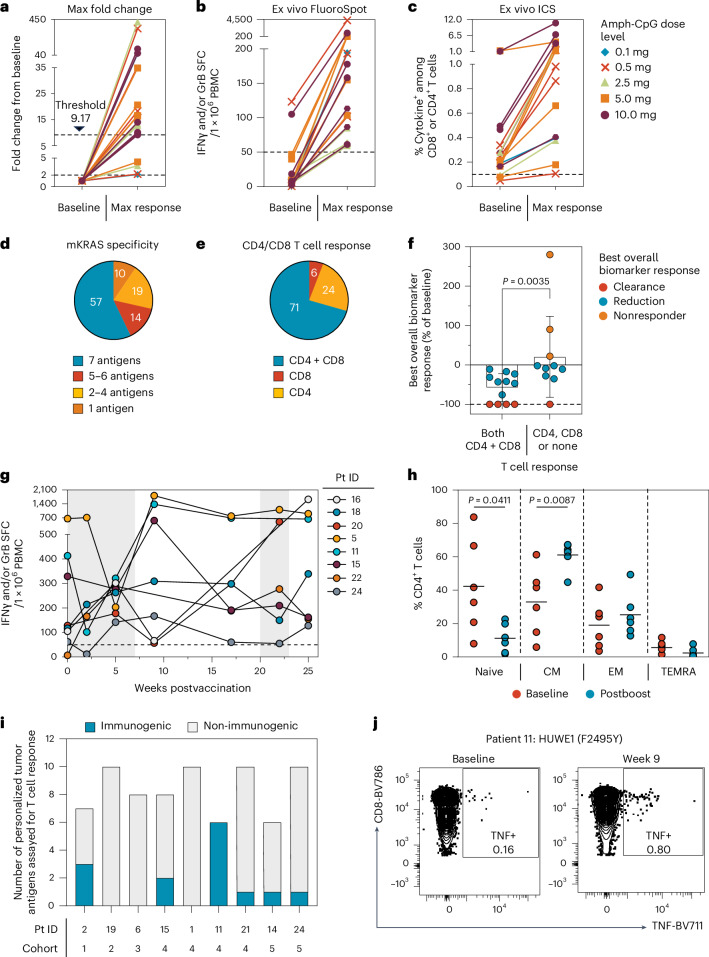
Expansion of mKRAS-specific T cells and induction of antigen spreading to nonimmunizing personal tumor neoantigens. PBMCs were assessed for direct, ex vivo, T cell response at baseline and maximum postimmunization timepoints. **a**, mKRAS-specific fold change from baseline to response for T cell responders (*n* = 21/25 patients; 9.17-fold threshold indicated by dashed line). **b**, mKRAS-specific, background-subtracted IFNγ and/or GrB SFCs per 1 × 10^6^ PBMCs. *n* = 19 responders. **c**, Frequencies of mKRAS-specific CD4^+^ or CD8^+^ T cells with intracellular IFNγ/TNF/IL2 assessed by flow cytometry. *n* = 17 responders. Response thresholds indicated by dashed lines in **a**–**c** and **g**. **d**, Frequency of T cell responders to 1–7 assessed mKRAS antigens. **e**, Frequency of T cell responders including CD4^+^, CD8^+^ or both CD4/8^+^ cytokine+ cells in ICS assay. **f**, Best overall tumor biomarker response for patients reported as percentage of the baseline value, stratified by CD4^+^/CD8^+^ T cell response; error bars are mean ± s.d. *n* = 22. **g**, Longitudinal IFNγ/GrB SFCs per 1 × 10^6^ PBMCs for the eight patients with booster vaccinations. Vaccination periods indicated in gray. **h**, CD4^+^ memory phenotype of baseline cytokine negative and postboost mKRAS-specific T cells, *n* = 6 patients (CM, CCR7^+^ CD45RA^−^; EM, CCR7^−^ CD45RA^−^; TEMRA, CCR7^−^ CD45RA^+^; naive, CCR7^+^ CD45RA^+^); line indicates mean. **i**,**j**, Antigen-spreading assessment of patient-specific number of assessed and positive direct, ex vivo T cell responses to nonvaccine tumor neoantigens in FluoroSpot or ICS assay of postprime PBMCs (**i**) and representative ICS data from patient 11 at baseline and week 9 (**j**). All *P* values calculated by two-tailed Mann–Whitney test. SFC, spot-forming cell. Source data

**Extended Data Fig. 5:**
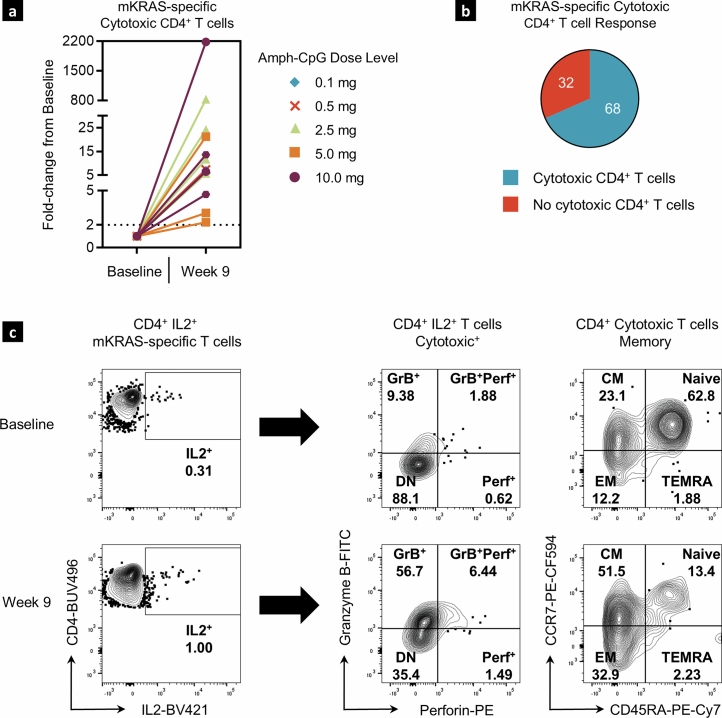
ELI-002 2P vaccination amplifies cytotoxic mKRAS-specific CD4^+^ T cells. Patients were immunized with 1.4 mg Amph-peptides 2P admixed with 0.1, 0.5, 2.5, 5 or 10 mg of Amph-CpG-7909. PBMCs were collected for T cell response assessment at baseline and week 9 timepoints. **a**, Shown is the ex vivo mKRAS-specific cytotoxic CD4^+^ T cell fold change from baseline to week 9 in the extended ICS assay for n = 19 patients. T cell cytotoxic responders are defined as a patient having ≥2-fold increase from baseline at week 9 (dotted line) and >0.1% GrB+ and/or perforin+. **b**, Pie chart depicts the percentage of tested ELI-002 2P patients that induce CD4^+^ cytotoxic+ T cells in extended ICS assay. **c**, Representative flow cytometry plots of PBMCs from patient 11 at baseline and week 9 PBMCs that have been stimulated with OLPs to G12D mKRAS antigen and tested in an ex vivo ICS assay for cytokine and cytotoxic marker production. Shown are CD4^+^ IL2^+^ mKRAS-specific T cells that are then gated on granzyme B, perforin and CD45RA and CCR7 memory markers. The frequencies of each population are shown on the dot plots. Source data

**Extended Data Fig. 6:**
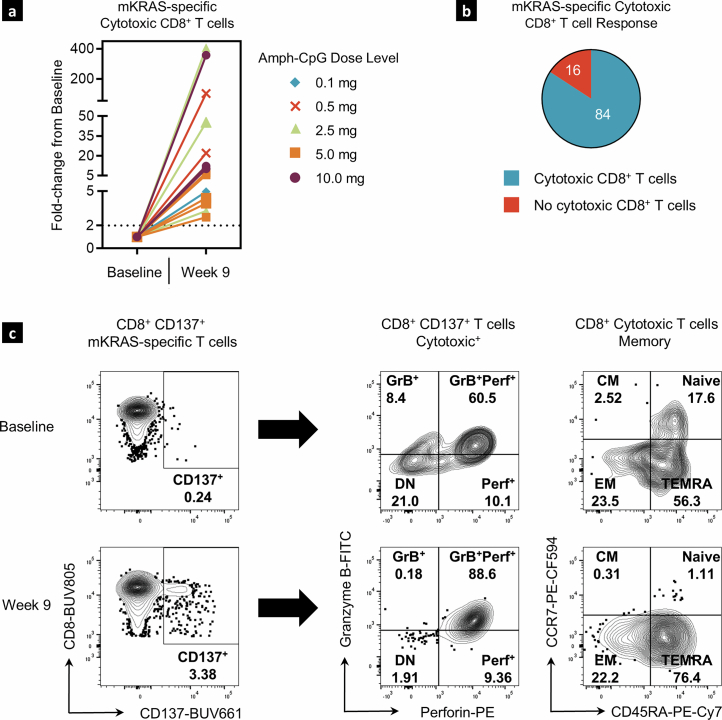
ELI-002 2P vaccination amplifies cytotoxic mKRAS-specific CD8^+^ T cells. Patients were immunized with 1.4 mg Amph-Peptides 2P admixed with 0.1, 0.5, 2.5, 5 or 10 mg of Amph-CpG-7909. PBMCs were collected for T cell response assessment at baseline and week 9 timepoints. **a**, Shown is the ex vivo mKRAS-specific cytotoxic CD8^+^ T cell fold change from baseline to week 9 in the extended ICS assay for n = 19 patients. T cell cytotoxic responders are defined as a patient having ≥2-fold increase from baseline at Week 9 (dotted line) and >0.1% GrB+ and/or perforin+. **b**, Pie chart depicts the percentage of tested ELI-002 2P patients that induce CD8^+^ cytotoxic^+^ T cells in extended ICS assay. **c**, Representative flow cytometry plots of PBMCs from Patient 19 at baseline and week 9 PBMCs that have been stimulated with OLPs to all 7 mKRAS antigen and tested in an ex vivo ICS assay for cytokine and cytotoxic marker production. Shown are CD8^+^ CD137^+^ mKRAS-specific T cells that are then gated on granzyme B, perforin and CD45RA and CCR7 memory markers. The frequencies of each population are shown on the dot plots. Source data

At extended follow-up, 84% (21/25) of patients generated mKRAS-specific T cell responses following ELI-002 2P immunization with 100% responders at the two highest dose levels (5.0, 10.0 mg of adjuvant Amph-CpG-7909). The median response was 13.38-fold over baseline for all patients; however, 17 patients exhibited responses above the ROC threshold of 9.17-fold (Fig. 2a–c). Fifty-seven percent of patients induced responses to all seven mKRAS antigens evaluated (Fig. 2d). Seventy-one percent of patients induced both CD4^+^ and CD8^+^ mKRAS-specific T cells and the induction of both CD4^+^ and CD8^+^ T cells significantly correlated with overall tumor biomarker response, indicating the potential importance of a balanced T cell response for improved tumor biomarker response (Fig. 2e,f). All 12 patients who induced both CD4^+^ and CD8^+^ T cells had T cell responses above the 9.17 threshold, suggesting an association between these biomarkers (Extended Data Table 2). ELI-002 2P vaccination amplified mKRAS-specific CD4^+^ and CD8^+^ T cells secreting granzyme B and perforin, indicating cytolytic potential; 68% (13/19) of patients induced granzyme B and perforin secreting mKRAS-specific CD4^+^ T cells after vaccination with predominant central and effector memory phenotype (Extended Data Fig. 5). Likewise, 84% (16/19) of patients had cytotoxic mKRAS-specific CD8^+^ T cells, substantially composed of TEMRA memory cells (Extended Data Fig. 6). ELI-002 2P-induced mKRAS-specific T cells were persistent before and after booster vaccination with seven of eight (88%) evaluable patients maintaining elevated T cell responses relative to baseline levels following booster vaccinations (Fig. 2g). Furthermore, postboost, long-lived mKRAS-specific CD4^+^ T cells showed significantly increased central memory phenotype compared to baseline with associated decreases in the naive subset (Fig. 2h). Although not required in the AMPLIFY-201 study, collection of tumor tissue at the time of progression in future studies will allow for assessment of the tumor-immune microenvironment, which may elucidate potential mechanisms of resistance. Finally, antigen spreading was assessed to examine the potential for ELI-002 2P to promote the expansion of immune responses targeting personalized tumor neoantigens not present in the vaccine. Nonimmunizing antigens from each patient’s personal tumor mutanome were selected for peripheral blood mononuclear cell (PBMC) immunogenicity assessment directly ex vivo. T cell responses targeting nonvaccine tumor neoantigens were significantly expanded from baseline levels in 67% (6/9) of the tested patients (Fig. 2i,j). Overall, 13 of 52 (25%) evaluated neoantigens showed increased T cell responses, including examples of both CD4^+^ and CD8^+^ T cells, as well as expansion of pre-existing, baseline-detectable responses or de novo expansion. Furthermore, five of six (83%) patients with positive antigen-spreading responses had mKRAS T cell counts above the ROC threshold, suggesting that robust mKRAS-specific T cell induction may be associated with diversification of the tumor-directed T cell response. Future assessment of larger patient groups will be critical to defining the potential contribution of antigen spreading to clinical outcomes.

Recently, multiple examples of next-generation therapeutic cancer vaccination have demonstrated promise in early phase and randomized clinical studies^12–15^. Innovative trial designs have focused on the adjuvant setting, where tumor burden is low, potentially allowing for robust and durable T cell responses to provide long-term protection from recurrence and death. Historically, PDAC has previously been considered a poor setting for immunotherapy; however, the favorable results from ELI-002 2P are consistent with the long-term follow-up of adjuvant patients with PDAC who received personalized mRNA vaccination in combination with atezolizumab and adjuvant mFOLFIRINOX^12^. In the long-term follow-up of ELI-002 2P treatment, mKRAS-specific T cell responses above a 9.17-fold threshold were significantly correlated to freedom from radiographic progression and death (radiographic RFS HR = 0.12, *P* = 0.0002; OS HR = 0.23, *P* = 0.0099). Outcomes in the PDAC subset, with a median radiographic RFS of 15.31 months and a median OS of 28.94 months, are notable given the historically rapid progression of patients with PDAC who have ctDNA^+^ MRD-relapse postsurgery, with a median radiographic RFS/DFS of 5.0–6.37 months and a median OS of 17.0 months^12,16^. Limitations of the study include the small sample size, nonrandomized design and that the median follow-up of the cohort is shorter than the median OS, suggesting that this estimate may continue to mature. Additionally, while the relatively small sample size and lack of an external validation cohort are limitations for the ROC analysis as performed, the subgroups were relatively well balanced for tumor type, tumor stage, prior therapies and baseline MRD characteristics (Extended Data Table 3). Notably, all five patients with G12R tumor mutations had T cell responses above the 9.17 threshold. Prior studies in PDAC have observed favorable clinical outcomes as well as decreased PDL1 expression for G12R^17,18^. This factor, together with potentially increased immunogenicity, may in part explain the more favorable clinical outcomes of PDAC patients with this mKRAS variant.

ELI-002 2P-induced mKRAS-specific T cell responses obtained in the monotherapy setting were observed in 100% of patients treated at the RP2D (adjuvant Amph-CpG-7909 was dose escalated), included both CD4^+^ and CD8^+^ T cells, and were sustained throughout the follow-up period, including development of memory and maintenance of critical antitumor effector functions. In the majority of T cell responders (17/21), T cell responses were observed against the specific tumor antigen detected at the time of enrollment. Analysis of tumor-specific T cell response and antigen spreading among larger patient groups, and with additional serial PBMC samples, will be helpful to further understand the instances where responses to the tumor antigen identified during screening were not observed. Additional longitudinal data will be useful to inform whether long-term dosing may augment cellular immunity. In addition to expanded T cells targeting mKRAS driver mutations, treatment with ELI-002 2P frequently led to antigen spreading, with ex vivo-detectable expansion of CD4^+^ and CD8^+^ T cells targeting additional personalized tumor neoantigens similar to those discretely targeted by personalized vaccination. These are consistent with prior observations of antigen spreading following amphiphile vaccination^19–21^ suggesting a role for lymph node immune activation as a mechanism supporting the development of tumor-specific T cells in situ.

Taken together, the long-term follow-up of the AMPLIFY-201 phase 1 study provides evidence that ELI-002 2P induces potent, polyfunctional CD4^+^ and CD8^+^ T cell immunity to mKRAS alongside frequent antigen spreading that may delay tumor recurrence. A randomized phase 2 study (NCT05726864) of a seven-peptide formulation (ELI-002 7P—KRAS/NRAS G12D, R, V, S, A, C and G13D) is ongoing in the adjuvant setting of PDAC. Beyond PDAC, off-the-shelf availability of ELI-002 supports broad development for various mKRAS-expressing tumor types. In conclusion, our observations support continued study of amphiphile lymph node-targeted immunotherapy for solid tumors.

## Methods

### Study design, patient eligibility, treatment and oversight

A phase 1, multicenter, open-label, first-in-human trial of ELI-002 2P monotherapy was conducted in five ascending dose cohorts at seven centers in the United States between 4 October 2021 and 24 September 2024 (the clinical cutoff date for the results presented here). A fixed dose of Amph-Peptides 2P (G12D and G12R, 0.7 mg each) was administered with escalating doses of 0.1, 0.5, 2.5, 5.0 and 10.0 mg Amph-CpG-7909 adjuvant. Eligible patients were 18 years or older, had mKRAS G12D-mutated or G12R-mutated pancreatic or colorectal cancers and were at high risk for relapse because of the presence of MRD (indicated by ctDNA-positivity or elevated serum CA19-9 and/or CEA). Clinical data was entered into Medidata Rave 2018.2.4. Additional details are provided in the study protocol (Supplementary Data 1).

At two institutions, City of Hope and the University of Colorado School of Medicine, central institutional review board (IRB) approval was obtained from WIRB Copernicus IRB. Local IRB approvals were provided for Memorial Sloan Kettering Cancer Center’s IRB, the University of Texas MD Anderson (Office of Human Subject Protection), the University of Iowa (Human Subjects Office/IRB), Northwell Health (Feinstein Institutes for Medical Research, Northwell Health IRB), the University of California, Los Angeles (Office of the Human Research Protection Program) and Massachusetts General Hospital (Dana–Farber Cancer Institute Office for Human Research Studies). The US Food and Drug Administration approved the study, which was registered on ClinicalTrials.gov (NCT04853017).

### Patients

We enrolled adult (≥18 years old) patients with Eastern Cooperative Oncology Group performance status of 0 or 1 with pathologically confirmed mKRAS (G12D or G12R) PDAC or CRC, who were MRD^+^ with either (1) absolute CA19-9 ≥ 90 U ml^−1^ or CEA ≥ 15 ng ml^−1^ or (2) successively rising values (≥1 week apart) in either CA19-9 or CEA not attributable to a noncancer condition, such as pancreatitis, peritonitis, postoperative leak/fistula or biliary obstruction. Patients had recovered from prior surgery, chemotherapy or radiation without ongoing medical/surgical issues and were willing to use effective methods to avoid pregnancy and provided written informed consent. Baseline absolute neutrophil count ≥1.5 × 10^9^ l^−1^, platelets ≥100 × 10^9^ l^−1^, normal range liver function tests, serum creatinine <1.5 (or if serum creatinine was ≥1.5 mg dl^−1^, creatinine clearance calculated by the Cockcroft–Gault formula ≥60 ml min^−1^ was acceptable), albumin ≥2.5 g dl^−1^ and IL6 <500 pg ml^−1^ were required.

PDAC patients had high risk tumor stages I, II, III or stage IV oligometastatic disease per current American Joint Committee on Cancer criteria with no evidence of disease on current imaging (equivocal radiographic findings such as subcentimeter lesions or potential resolving soft tissue changes after surgery were accepted), prior treatment with standard chemotherapy/chemoradiation administered in the neoadjuvant and/or adjuvant setting, and complete tumor resection (R0 or R1 pathologic margins), with focal use of intraoperative irreversible electroporation permitted.

CRC patients had high risk stage II (T4N0), stage III (T4N1-2/TanyN2) or stage IV oligometastatic disease per current American Joint Committee on Cancer staging criteria, prior cytotoxic chemotherapy administered in the neoadjuvant or adjuvant setting, or as total neoadjuvant therapy, and complete surgical resection (R0 or R1 pathologic margins), with focal use of intraoperative irreversible electroporation permitted.

We excluded patients who received antitumor therapy within 4 weeks, who had history of brain metastasis, other malignancies within the last 3 years (except for adequately treated carcinoma of the cervix, bladder, prostate, basal or squamous cell skin cancer), were receiving immunosuppressive drugs, those with serious comorbid illness including uncontrolled infection, class III or IV (New York Heart Association) cardiac failure, myocardial infarction within 6 months, active seizure disorders, autoimmune diseases or interstitial lung disease if requiring systemic steroids, pulse oximetry <92% on room air, prior organ transplants, HIV/AIDS, hepatitis B, hepatitis C (unless they had a sustained virologic response to direct-acting antiviral therapy) and those in the first two weeks of SARS-CoV-2. Women were excluded if pregnant or lactating. PDAC patients were excluded when tumors were of neuroendocrine subtype, or when there was a germline BRCA 1/2 mutation; CRC patients were excluded when tumors were mismatch repair defective (MSI^+^).

Treatment was divided into a ‘prime immunization series’ (six subcutaneous doses of ELI-002 2P over 8 weeks), a 3-month ‘no dosing period’ (observation) and a ‘booster immunization series’ (4 weekly doses of ELI-002 2P). A follow-up period of up to 2 years was included after the first dose of ELI-002 2P to monitor safety and efficacy.

The study was sponsored and designed by Elicio Therapeutics in collaboration with the academic authors. The study and analyses were conducted in accordance with the general principles of the Declaration of Helsinki and Good Clinical Practice guidelines of the International Council for Harmonization. The trial protocol, amendments and supporting documents were approved by the local/central institutional review board for each study site, the US Food and Drug Administration and were registered on Clinicaltrials.gov (NCT04853017). All patients provided written informed consent.

A safety and monitoring committee was convened to review safety and determine dose escalation and cohort expansion decisions. Cohorts ranged from three to six patients with expansions allowed after the first three patients completed 28 days without dose-limiting toxicity and when additional eligible patients had been identified.

All the authors affirm that the trial was conducted in accordance with the study protocol and vouch for the accuracy and completeness of the data. All the authors reviewed and revised the manuscript and made the decision to submit it for publication.

The initial protocol (version 1.0) was approved on 13 July 2020. Key protocol amendments are as follows: Amendment 2 (version 3.0) was approved on 23 February 2021 and included changes requested by the US Food and Drug Administration. This was the initial protocol for initiating the study. On 8 April 2022, Amendment 4 (version 5.0) was approved and added serum tumor biomarkers (that is, CEA and CA19-9) to the MRD eligibility along with ctDNA. Amendment 5 (version 6.0), approved on 2 August 2022, added language regarding pseudo-progression and continued ELI-002 dosing. Amendment 6 (version 7.0) was approved on 25 January 2023 and added language for public record search for OS. Amendment 7 (version 8.0) was approved on 7 August 2023 and added another year of follow-up to collect additional RFS and OS.

### Endpoints and assessments

Primary endpoints of the study were safety (adverse events were graded per Common Terminology Criteria for Adverse Events, version 5.0), tolerability and determination of the RP2D. Secondary and exploratory endpoints include tumor biomarker reduction and clearance defined through assessment of ctDNA and/or serum tumor antigens (CA19-9 or CEA), radiographic relapse-free survival, defined as the time from initiation of ELI-002 treatment until confirmed radiographic progression using iRECIST criteria, and OS, and immunogenicity.

### Immunogenicity analysis

PBMCs for immunogenicity analysis were processed from leukapheresis (baseline, week 9) or whole-blood collections (all other timepoints). Patient PBMCs were processed by the Ficoll-Hypaque gradient protocol for leukapheresis samples or cell processing tubes (BD) for whole-blood samples. PBMCs were resuspended in CS10 freezing media (Cryostor), frozen in aliquots of 10–20 million cells per cryovial and stored in a temperature-monitored liquid nitrogen vapor phase freezer. Only PBMCs collected before subsequent therapy are included in datasets and graphs, with the exception of the long-term duration graphs (Fig. 2g,h). The maximum T cell response was determined as the maximum fold change from baseline to any postvaccination timepoint in either the ‘Ex vivo FluoroSpot assay’ or ‘Ex vivo ICS assay’ for any of the seven mKRAS antigens or a pool of all seven antigens combined. Figure 2a includes data from all T cell responders (*n* = 21/25). Figure 2b contains data from all responders with FluoroSpot data (*n* = 19/25) while Fig. 2c contains data from all responders with ICS data (*n* = 17/25). Figure 2g contains data from all patients with booster doses that had responses in FluoroSpot assay (*n* = 8) while Fig. 2h contains data from the boosted patients in Fig. 2g that were also tested for memory markers in the intracellular cytokine staining (ICS) assay (*n* = 6/8).

### Ex vivo FluoroSpot assay

A direct IFNγ/granzyme B (GrB) FluoroSpot assay was performed on thawed PBMCs. Cryopreserved PBMCs were thawed in 10% human AB serum/RPMI media + Benzonase and rested overnight at 37 °C. Precoated human IFNγ/GrB FluoroSpot plates were washed with phosphate-buffered saline and blocked with AIM-V media for at least 30 min (MabTech). The 2 × 10^5^ rested PBMCs were plated into each well and stimulated for 44 h as per the manufacturer’s instructions with seven individual mKRAS peptide pools and a WT peptide pool. Each pool consisted of a KRAS 18-mer peptide along with the corresponding 9-mer and 10-mer overlapping peptides (OLPs), at a concentration of 2 µg per peptide per ml. No exogenous cytokines were added to the PBMCs during this assay. All samples were plated in triplicate. Dimethyl sulfoxide was used as the negative control (background wells) and anti-CD3 (MabTech) was used as the positive control. The plate was developed based on the manufacturer’s instructions. Plates were scanned and counted using the IRIS plate reader (MabTech) using the FITC and Cy3 filters. Data are background subtracted, averaged per triplicate measurements and normalized to 1 × 10^6^ PBMCs. A postvaccination sample was characterized as positive if it was at least 2 s.d. above the DMSO negative control. A responder in the FluoroSpot assay was defined as a patient with a ≥2-fold increase from baseline at any postvaccination timepoint and more than the minimum threshold of 50 total IFNγ and GrB spot-forming cells per 1 × 10^6^ PBMCs.

### Ex vivo ICS assay

A direct ICS assay for IL2, IFNγ and TNF was performed by flow cytometry. PBMCs were thawed and rested overnight. In total, 10^6^ PBMCs per well were plated and stimulated for 17 h at 37 °C with individual mKRAS peptide pools at 2 μg ml^−1^ per peptide (Supplementary Table 1). GolgiStop and GolgiPlug (BD) were also added to each well. The next day, cells were surface stained with antibodies against CD4 (BV421—clone, SK3; BD, 566907; 2.5 μl per well), CD8 (BV786—clone, RPA-T8; BD, 563823; 1:25), CD45RA (Alexa 700—clone, HI100; BioLegend, 304120; 1:25), CCR7 (PE-CF594—clone, 15053; BD, 562381; 1:12.5), Aqua Live/Dead marker (Thermo Fisher Scientific, L34966; 0.5 μl per well) and dump markers CD14 (PE-Cy5—clone, 61D3; Thermo Fisher Scientific, 15-0149-42; 1:200), CD16 (PE-Cy5—clone, 3G8; BioLegend, 302010; 1:200), and CD19 (PE-Cy5—clone, SJ25C1; BioLegend, 363042; 1:100). Cells were subsequently fixed with CytoFix/CytoPerm (BD) and further stained with antibodies against CD3 (APC-H7—clone, SK7; BD, 560176; 2.5 μl per well), IFNγ (FITC—clone, Mab11; BioLegend, 506504; 1:200), TNF (BV711—clone, B27; BioLegend, 502940; 1:50) and IL2 (BV650—clone, MQ1-17H12; BioLegend, 500334; 1:50). Cells fixed in 0.5% formaldehyde were acquired on a BD FACSymphony and data were analyzed with BD FlowJo V10 software (gating progression and example plots in Supplementary Fig. 1). A responder in the ICS assay was defined as a patient having ≥2-fold increase in total IFNγ, IL2 and TNF from baseline at any postvaccination timepoint, along with a cytokine+ T cell frequency of ≥0.1%.

Some patients were also tested in an extended ‘Ex vivo ICS assay’ that included additional activation and cytotoxic markers. The extended ‘Ex vivo ICS assay’ was set up using the same methods as above, with the addition of CD107a (Alexa Fluor 700—clone, H4A3; BD, 561340; 1.25 μl per well) during the 17 h stimulation. The next day, cells were surface stained with antibodies against CD8 (BUV805—clone, SK1, BD, 612889), CD45RA (PE-Cy7—clone, HI100; BD, 560675), CCR7 (BUV615—clone, 3D12; BD, 562381), Aqua Live/Dead marker (Thermo Fisher Scientific, L34966; 0.5 μl per well) and dump markers CD14 (PE-Cy5—clone, 61D3; Thermo Fisher Scientific, 15-0149-42; 1:400), CD16 (PE-Cy5—clone, 3G8; BioLegend, 302010; 1:100) and CD19 (PE-Cy5—clone, SJ25C1; BioLegend, 363042; 1:100). Cells were subsequently fixed with the FoxP3/transcription factor staining buffer set (Thermo Fisher Scientific) and further stained with antibodies against CD3 (APC-H7—clone, SK7; BD, 560176; 1:40), CD4 (BUV496—clone, SK3; BD, 612936; 1:40), IFNγ (BB700—clone, B27; BD, 566394; 1:80), TNF (BV750—clone, MAb11; BioLegend, 502940; 1:80), IL2 (BV421—clone, MQ1-17H12; BD, 564164; 1:40), granzyme B (FITC—clone, GB11; BD, 560211; 1:40), perforin (PE—clone, B-D48; BioLegend, 353304; 1;80), CD137 (BUV661—clone, 4B4-1; BD, 741642; 1;80), CD154 (BUV563—clone, TRAP-1; BD, 748984; 1:80), CD69 (BV711—clone, FN50; BD, 563836; 1:160), Ki67 (BV650—clone, B56; BD, 563757; 1;80) and FoxP3 (PE/Dazzle 594—clone, 206D; BioLegend, 320126; 1:160).

### Tumor biomarker assessment and mutation identification

Comprehensive genomic profiling, whole-exome sequencing (WES), was performed to determine whether the patient’s tumor harbored at least one of the two mKRAS alleles targeted by the ELI-002 2P (G12D or G12R). The Natera Signatera ctDNA test evaluated for the presence or absence of circulating tumor DNA. WES was performed on formalin-fixed, paraffin-embedded tumor samples with at least 20% tumor content confirmed by a pathologist under the Central Lab Improvement Amendments and College of American Pathologists guidelines. Genomic DNA was extracted from the patient’s normal (whole blood) and tumor tissue. Libraries of tumor and matched germline DNA were prepared and exomic regions were captured. The assay was performed by target enrichment of the isolated DNA, followed by 440× coverage sequencing on an Illumina HiSeq 2500 or NovaSeq 6000 (Illumina). Somatic single-nucleotide variants (SNVs) that were present in the tumor and absent in the germline were identified. A proprietary Natera algorithm selected a set of 16 SNVs to maximize the detectability of tumor DNA if present in plasma. Polymerase chain reaction primers targeting the 16 personalized SNVs were designed and synthesized to be used to identify and track ctDNA in a patient’s plasma. Cell-free DNA was extracted from plasma and analyzed using a multiplexed personalized polymerase chain reaction assay. Plasma samples with ≥2 SNVs detected above a predefined confidence threshold were deemed ctDNA^+^, and ctDNA concentration was reported as mean tumor molecules per milliliter of plasma. In patients without adequate tumor tissue, a plasma-based ctDNA assay for mKRAS variants was performed using Sysmex SafeSEQ RAS-RAF. Cell-free DNA was isolated from plasma and a next-generation sequencing-based assay that evaluated K/NRAS to detect SNVs was performed using NextSeq 550 (Illumina). The ctDNA concentration was reported as the number of mutant molecules per variant and the mutant allele frequency. Local testing was permitted if already available to confirm mKRAS status. Serum tumor biomarkers, CA19-9 and CEA, were analyzed by the local laboratories at each study site.

### Antigen-spreading assay

To assess for antigen spreading, PBMCs were stimulated in the ‘Ex vivo FluoroSpot assay’ and ‘Ex vivo ICS assay’ as above, with neoantigens not included in the ELI-002 2P vaccine. Genomic DNA is extracted from the patient’s normal and tumor samples using next-generation sequencing WES. Using the WES data for each patient, somatic single-nucleotide mutations present in the tumor and absent in the germline genomic DNA were identified using a validated bioinformatics tool (GEM ExTra pipeline NG2-LDT 1.14.0; Natera). The reference genome assembly used for alignment is NCBI GRCh37. Stop-gain and start-loss mutations were excluded. Up to ten neoantigens were randomly selected from the list of somatic SNVs generated by WES for each tested patient for antigen spreading testing. An algorithm was not used to select these neoantigens. First, an 18-mer was designed (generally with the mutation centered in the middle) and then Genscript synthesized two 15-mers overlapping by 11 to cover the mutated 18-mer (18-mer sequences found in Supplementary Table 1).

### Statistical analysis

Descriptive statistics were used to summarize demographic, medical history and safety data. Continuous variables were summarized using mean, s.d., median, minimum value and maximum value. Categorical variables were summarized using frequency counts and percentages. Clinical efficacy outcomes, such as tumor biomarker reduction or clearance, were examined for association with categorical variables, including high versus low T cell response, using the Mann–Whitney test. The Kaplan–Meier method was used to estimate the survival distributions. The log-rank test was used to compare the RFS between the high and low T cell responders and the ROC analysis was performed using a logistic regression model. SAS v9.4 and R v4.4.3 were used to create Fig. 1 and Extended Data Figs. 2–4 and perform statistical analysis. GraphPad Prism v9.4 was used to create Figs. 1 and 2 and Extended Data Figs. 5 and 6 and perform statistical analysis.

### Reporting summary

Further information on research design is available in the Nature Portfolio Reporting Summary linked to this article.

## Online content

Any methods, additional references, Nature Portfolio reporting summaries, source data, extended data, supplementary information, acknowledgements, peer review information; details of author contributions and competing interests; and statements of data and code availability are available at 10.1038/s41591-025-03876-4.

## Supplementary information

Supplementary Information Supplementary Fig. 1 and Table 1.

Reporting Summary

Supplementary Data 1 ELI-002-001 Protocol Version 8.0.

## Source data

Source Data Fig. 1 Statistical source data.

Source Data Fig. 2 Statistical source data.

Source Data Extended Fig. 2 Statistical source data.

Source Data Extended Fig. 3 Statistical source data.

Source Data Extended Fig. 4 Statistical source data.

Source Data Extended Fig. 5 Statistical source data.

Source Data Extended Fig. 6 Statistical source data.

Source Data Extended Table 2 Statistical source data.

## Figures and Tables

**Extended Data Table 1:**
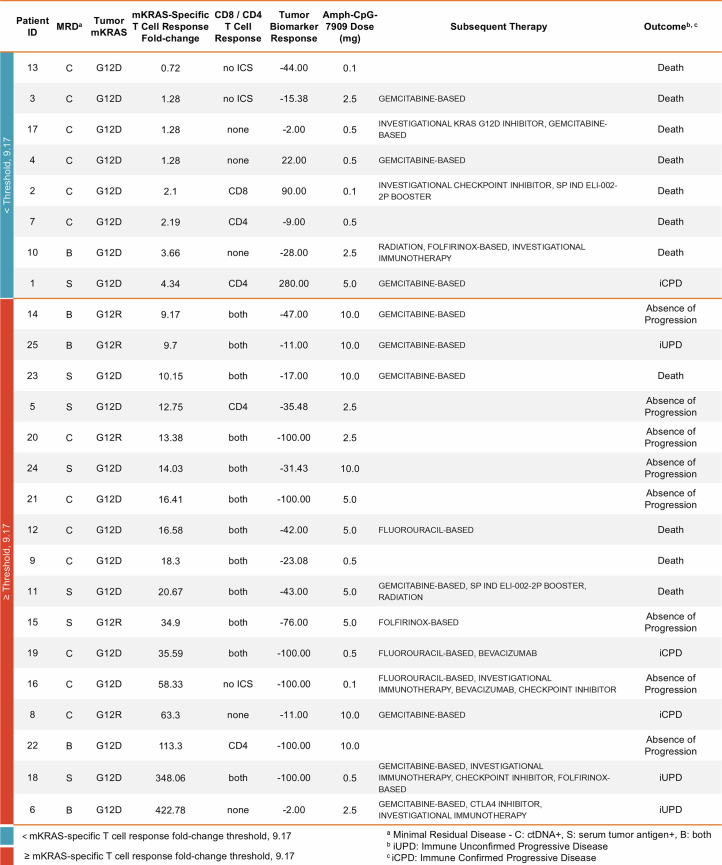
Patient-specific mKRAS-specific T cell responses, subsequent treatment and clinical outcomes as of data cutoff

**Extended Data Table 2:**
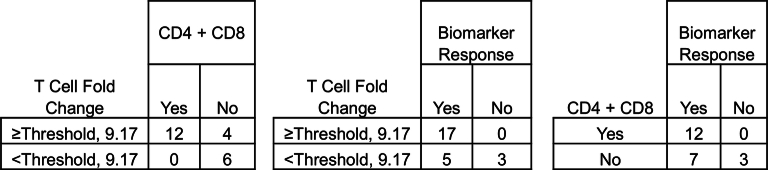
Tumor biomarker and T cell responses

Source data

**Extended Data Table 3:**
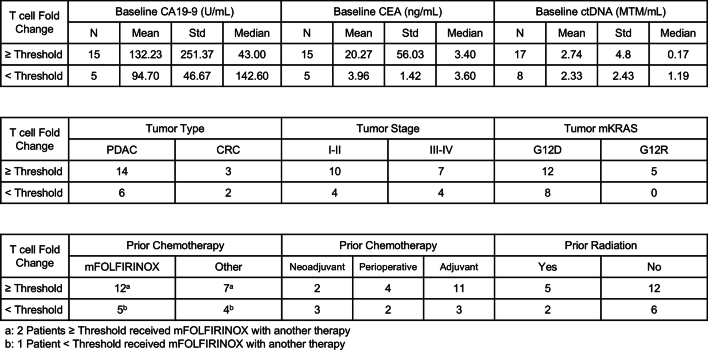
Patient characteristics

## Data Availability

Requests must be made to datarequest@elicio.com, with responses provided within 30 days of request. To ensure consistency with the underlying study consent, de-identified patient data that can be shared will be disclosed under data transfer agreements. Investigators and institutions who agree to the terms of the data transfer agreement, which will include, but will not be limited to, terms to address the use of these data for the purposes of a specific project and for research purposes only, to prohibit attempts to re-identify the data and to protect the confidentiality of the data, will be granted access to the data. Elicio Therapeutics will then facilitate the transfer of the requested de-identified data to the requestor using secure electronic data transmission. The data will then be available for up to 12 months. Source data are provided with this paper.
