# Supplementary material for: Lymph node-targeted, mKRAS-specific amphiphile vaccine in pancreatic and colorectal cancer: phase 1 AMPLIFY-201 trial final results
Source: Nat Med. 2025 Aug 11;31(11):3648–53. doi: 10.1038/s41591-025-03876-4 (PMC12618236; doi:10.1038/s41591-025-03876-4)
Supplement: Supplementary file 1 — Supplementary Fig. 1 and Table 1. [file 41591_2025_3876_MOESM1_ESM.pdf]

nature medicine

Supplementary information https://doi.org/10.1038/s41591-025-03876-4

Lymph node-targeted, mKRAS-specific amphiphile vaccine in pancreatic and colorectal cancer: phase 1 AMPLIFY-201 trial final results

In the format provided by the authors and unedited

Supplementary Information Table of Contents

Supplementary Figure 1: ICS and memory phenotype gating strategy . . . . Page 2

Supplementary Table 1: Antigen Spreading Neoantigen sequences. . . . . .Pages 3-8

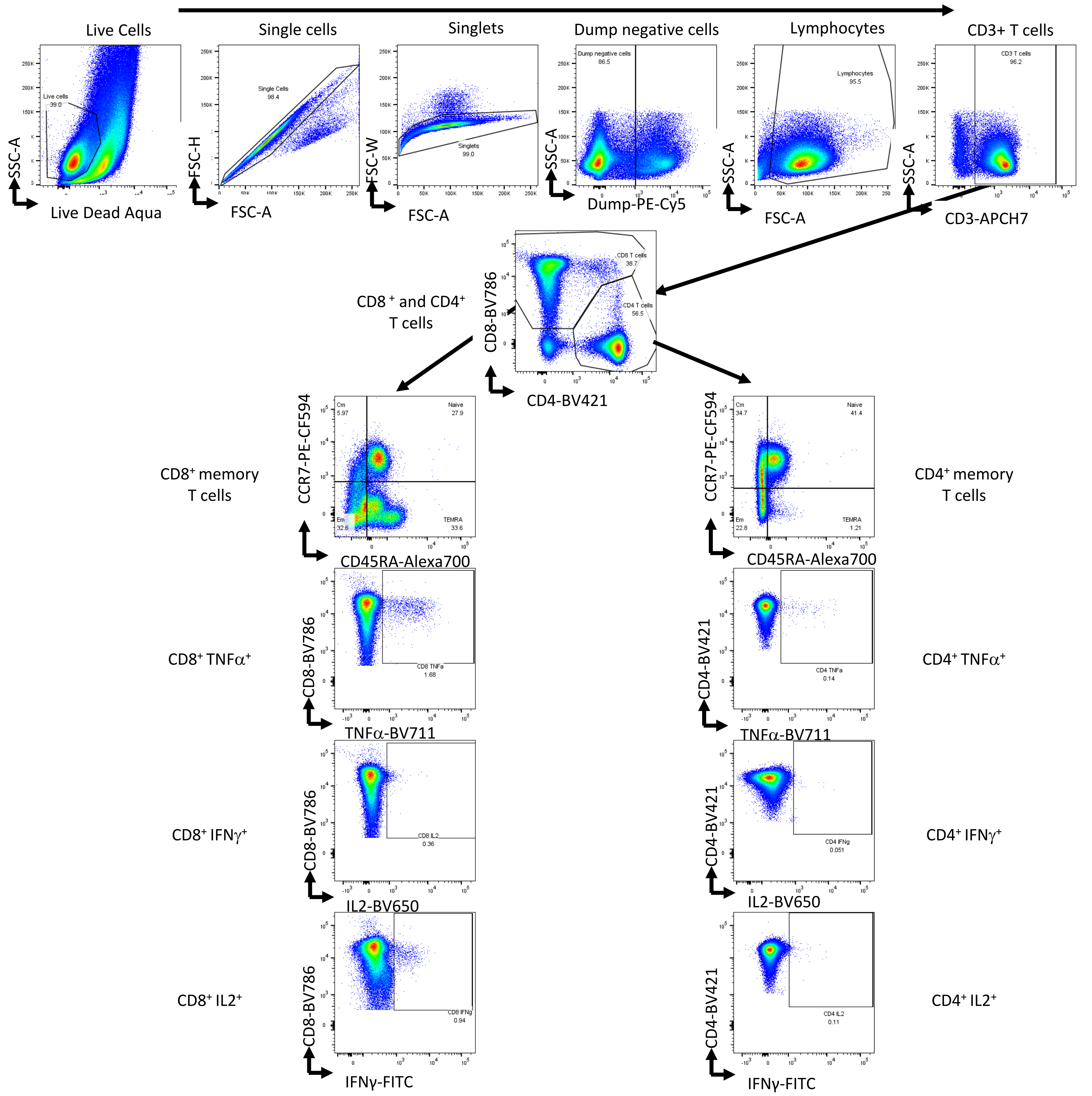

Supplementary Figure 1: ICS and memory phenotype gating strategy, example flow cytometry scatter plots, and example fluorospot well images.

Gating strategy and example scatter plots for ICS assay and T cell memory phenotype.

Supplementary Data Table 1
Antigen spreading Neoantigen sequences

Patient ID	Cohort	# neoantigens tested	# neoantigens positive	Neoantigen 1
1	4	10	0	MRPL16 (P57L) pklrfierallvpkvrre
2	1	7	3	THSD7B (K1584N) tsylvckkpnphqstppq
6	3	8	0	SEC24B (T465A) akpfgygypalqpgyqna
11	4	6	6	ANKFN1 (S485Y) qdflwftklycmwedirw
14	5	6	1	HIBADH (I122T) nnmllaismtgtaeamnl
15	4	8	2	ZNF232 (K159N) avtvledlenglepepqv
19	2	10	0	CAND2 (R448W) kalqrqlkdwsvrarqgc
21	4	10	1	KLH4 (Y306C) tellnvahkctmehfiev
24	5	10	1	TP53 V173fs kqsqhmtevrrcphherc

Supplementary Data Table 1

Antigen spreading Neoantigen sequences

Neoantigen 2	Neoantigen 3
CPB1 (M310I) ltihsysqmiiypysyay	AAK1 (S523C) qfpvvsqggcqqqlmqnf
PPL (S1521R) teqeiqrlkrsleeesrs	WDR81 (R1670P) dfflsgskdptvrlwply
ABCC10 (R242Q) largacgelqqpqdicrl	TMEM63A (D402A) skwtvtfaaapedicwkn
EBF1 (G171D) srccdkkscdnrnetpsd	MDFIC (G221R) cncpcdmdcrimdacces
LAMA5 (E1019K) vvllpsayykaallqlrv	OIT3 (H302N) scrgvsngtnvnilfslk
GRIN2B hsqlsdlygsfsfksdry	SEZ6 (I716N) ndtcpelpenpngwksps
PCLO (T5139S) rkrivnwhkllvsptqsh	CCDC39 (K639N) erlskieklnnryeiltv
PREB (H195D) lekvlefkadegeiedla	SYNJ1 (D1049V) edaevaadfvmegdvddy
CMYA5 (E2792K) kegfpskeskrtlarpfd	DUSP27 (R99H) qllvedlynhvrekmddt

Supplementary Data Table 1

Antigen spreading Neoantigen sequences

Neoantigen 4	Neoantigen 5
ZC3H4 (D741N) lfpehplepnsfseggpp	IMMP2L (R109W) ivrtighknwyvkvprgh
ATP2A1 (A517T) vgnkmfvkgtpegvidrc	ARR3 (D40N) epidgvvlvnpeylkcrk
ZFAND2A (Y39H) qdfckdhfphaahkcpfa	MMP25 (Q533R) tpvsetcdcrcelnqaag
HUWE1 (F2495Y) dreddliieydnmfssat	CALU (L267F) dkeetkdwifpsdydhae
SBNO1 (K544R) qlsftgvtfrieevllsq	NOTCH3 (D139Y) hgarcsvgpygrflcscp
NYAP2 (R197Q) kkipppkpkqnpntqlst	FAT3 (H1846Y) anldhetiayfhfhvhvr
MAGEC1 (S535R) thsplqivprlpewedsl	NEB (P7583H) kskghyhtihdnleqlhl
ITIH6 (S866Y) kpsapphqiytsislskp	SPTA1 (V17A) tvvessgpkaletaeeiq
EIF3L (E134K) davflilykklyyrhya	INSRR (R140H) lgavlrgavhveknqelc

Supplementary Data Table 1
Antigen spreading Neoantigen sequences

**Neoantigen 6**	**Neoantigen 7**
TP53 (G262V) tiitledssvnllgrnsf	SH3PXD2A (A937V) gfgktsgtpvvkmrngvr
TRAF3IP1 (G601Q) tlcksalplqkimdyiqe	GEN1 (I385T) lvllthydmterklgsrn
KMT2E (N1205S) gdgcassndsgeqvdhta	CADPS2 (D1223N) icvwltdrlnlqlhiyql
FLNA (V1124M) ldngdgtcsmsyvptepg	
FUK (H394Y) qhchlqgpiyigagclvt	
THBS2 (A88S) rqkegffltsqlkqdgks	CAPN13 (I269F) qyrrgweeifslwnpwgw
FZR1 (S35T) emrrtltpatspvsspsk	EVC (Q579H) gksnrfrrqhwklfqell
COL5A1 (P1043S) kegtkgdpgsaglpgkdg	MMP21 (S5F) mlaafifrptlllcwlaa
MAGEA12 (VR170DP) fgievvevdpighlyilv	NPTX1 (T333M) gvweayqdgmqggsgenl

Supplementary Data Table 1
Antigen spreading Neoantigen sequences

**Neoantigen 8**	**Neoantigen 9**
VWA8 (K1683E) qatgelddaeiidgltge	RNASE10 (R210H) khiiitcndmkhqlptgq
MIA3 (E890K) sqgsaaaepkddsfhwtp	
NDST4 (T475N) nsimvlprqncglfthti	
FIBCD1 (R303Q) nffrgwdayqdgfgrltg	RIMS2 (Q188K) kpklheqtkfqgpsgdl
DYSF (I1336N) ypppqreannymvpqnik	GRIN2A (N1076S) ratchrepdssknhktkd
POU4F2 (R247C) elfngaekkckrtsiaap	SORSCS1 (V781I) vvsnnctdgireqytakp

Neoantigen 10

RNF213 (D608N) lkkhvvplpngkstdflp

SOX17 (A108V) skmlgkswkvltlaekrp
MLTK (N21S) kfddlqffescgggsfgs
VWDE (C1540F) eciapsichfpsswegvr
