# Supplementary material for: Lymph node-targeted, mKRAS-specific amphiphile vaccine in pancreatic and colorectal cancer: phase 1 AMPLIFY-201 trial final results
Source: Nat Med. 2025 Aug 11;31(11):3648–53. doi: 10.1038/s41591-025-03876-4 (PMC12618236; doi:10.1038/s41591-025-03876-4)
Supplement: Supplementary file 2 — Reporting Summary [file 41591_2025_3876_MOESM2_ESM.pdf]

nature portfolio

Corresponding author(s): Chris Haqq, Zev Wainberg, Shubham Pant, Eileen O'Reilly

Last updated by author(s): June 25, 2025

# Reporting Summary

Nature Portfolio wishes to improve the reproducibility of the work that we publish. This form provides structure for consistency and transparency in reporting. For further information on Nature Portfolio policies, see our Editorial Policies and the Editorial Policy Checklist.

## Statistics

For all statistical analyses, confirm that the following items are present in the figure legend, table legend, main text, or Methods section.

| n/a | Confirmed | |
|---|---|---|
| ☐ | ☒ | The exact sample size (*n*) for each experimental group/condition, given as a discrete number and unit of measurement |
| ☐ | ☒ | A statement on whether measurements were taken from distinct samples or whether the same sample was measured repeatedly |
| ☐ | ☒ | The statistical test(s) used AND whether they are one- or two-sided<br>*Only common tests should be described solely by name; describe more complex techniques in the Methods section.* |
| ☐ | ☒ | A description of all covariates tested |
| ☐ | ☒ | A description of any assumptions or corrections, such as tests of normality and adjustment for multiple comparisons |
| ☐ | ☒ | A full description of the statistical parameters including central tendency (e.g. means) or other basic estimates (e.g. regression coefficient) AND variation (e.g. standard deviation) or associated estimates of uncertainty (e.g. confidence intervals) |
| ☐ | ☒ | For null hypothesis testing, the test statistic (e.g. *F*, *t*, *r*) with confidence intervals, effect sizes, degrees of freedom and *P* value noted<br>*Give P values as exact values whenever suitable.* |
| ☒ | ☐ | For Bayesian analysis, information on the choice of priors and Markov chain Monte Carlo settings |
| ☒ | ☐ | For hierarchical and complex designs, identification of the appropriate level for tests and full reporting of outcomes |
| ☒ | ☐ | Estimates of effect sizes (e.g. Cohen's *d*, Pearson's *r*), indicating how they were calculated |

*Our web collection on statistics for biologists contains articles on many of the points above.*

## Software and code

Policy information about availability of computer code

| | |
|---|---|
| Data collection | MediData RAVE 2018.2.4 |
| Data analysis | FlowJo v10<br>GraphPad Prism v9.4<br>SAS v9.4<br>R v4.4.3 |

For manuscripts utilizing custom algorithms or software that are central to the research but not yet described in published literature, software must be made available to editors and reviewers. We strongly encourage code deposition in a community repository (e.g. GitHub). See the Nature Portfolio guidelines for submitting code & software for further information.

# Data

Policy information about availability of data

All manuscripts must include a data availability statement. This statement should provide the following information, where applicable:

- Accession codes, unique identifiers, or web links for publicly available datasets
- A description of any restrictions on data availability
- For clinical datasets or third party data, please ensure that the statement adheres to our policy

Requests must be made to datarequest@elicio.com, with responses within 30 days of request. To ensure that data sharing is consistent with the underlying study consent, de-identified patient data that can be shared will be done under data transfer agreements. Investigators and institutions who agree to the terms of the data transfer agreement, which will incluce, but will not be limited to, terms to address the use of these data for the purposes of a specific project and for research purposes only, to prohibit attempts to re-identify the data and to protect the confidentiality of the data, will be granted access to the data. Elicio Therapeutics will then facilitate the transfer of the requested de-identified data to the requestor using secure electronic data transmission; the data will then be available for up to 12 months.

# Research involving human participants, their data, or biological material

Policy information about studies with human participants or human data. See also policy information about sex, gender (identity/presentation), and sexual orientation and race, ethnicity and racism.

| | |
|---|---|
| Reporting on sex and gender | Sex and gender is reported in the baseline demographics table in the original paper (Table 1) Pant 2024 Nature Medicine. |
| Reporting on race, ethnicity, or other socially relevant groupings | Race and ethnicity is reported in the baseline demographics table in the original paper (Table 1) Pant 2024 Nature Medicine. |
| Population characteristics | All patients had clinical characteristics typical for resectable pancreatic and colorectal cancer patients. Detailed patient characteristics are provided in the original paper (Table 1 and Extended Data Table 1), Pant 2024 Nature Medicine. |
| Recruitment | Complete eligibility and enrollment criteria are provided in the study protocol (Supplementary Materials of original paper, Pant 2024 Nature Medicine). Eligible subjects were recruited from 8 centers across diverse geographic regions of the United States. Key eligibility required ECOG performance status 0-1, resectable pancreatic or colorectal cancer, presence of a G12D or G12R somatic KRAS mutation, and positive laboratory evidence of minimal residual disease. Excluded patients had pancreatic neuroendocrine tumors, MSI+ colorectal tumors, other malignancies within the last 3 years anticipated to require treatment, were pregnant or lactating females, or refused to use acceptable methods of contraception. There was no appreciable bias in trial enrollment, and limitations of the small phase 1 population sample are included in the discussion. |
| Ethics oversight | At two institutions, City of Hope and University of Colorado School of Medicine, central institutional review board (IRB) approval was from WIRB Copernicus (WCG IRB). Local IRB approvals were provided for Memorial Sloan Kettering Cancer Center (MSKCC IRB), the University of Texas MD Anderson (University of Texas MD Anderson Office of Human Subject Protection), the University of Iowa (University of Iowa Human Subjects Office/IRB), Northwell Health (Feinstein Institutes for Medical Research, Northwell Health IRB), the University of California, Los Angeles (UCLA Office of the Human Research Protection Program) and Massachusetts General Hospital (Dana-Farber Cancer Institute Office for Human Research Studies). The US Food and Drug Administration (FDA) approved the study which was registered on ClinicalTrials.gov (NCT04853017). |

Note that full information on the approval of the study protocol must also be provided in the manuscript.

# Field-specific reporting

Please select the one below that is the best fit for your research. If you are not sure, read the appropriate sections before making your selection.

☒ Life sciences ☐ Behavioural & social sciences ☐ Ecological, evolutionary & environmental sciences

For a reference copy of the document with all sections, see nature.com/documents/nr-reporting-summary-flat.pdf

# Life sciences study design

All studies must disclose on these points even when the disclosure is negative.

| | |
|---|---|
| Sample size | We enrolled 25 patients using an empirical Phase 1 dose escalation design appropriate to evaluate the primary endpoint of safety and as detailed in the study protocol (Supplementary Materials of original paper, Pant 2024 Nature Medicine). |
| Data exclusions | No data were excluded from the analyses. |
| Replication | The study findings were reproducible. KRAS mutation status and circulating tumor DNA assays are CLIA validated and tested in a single independent assay. The T cell response data was only tested in a single independent experiment due to sample availability limitations. |

| | |
|---|---|
| Randomization | Not applicable, because this is a single arm phase 1 clinical trial. |
| Blinding | Not applicable, because this is an open-label phase 1 clinical trial. |

# Reporting for specific materials, systems and methods

We require information from authors about some types of materials, experimental systems and methods used in many studies. Here, indicate whether each material, system or method listed is relevant to your study. If you are not sure if a list item applies to your research, read the appropriate section before selecting a response.

## Materials & experimental systems

| n/a | Involved in the study |
|---|---|
| ☐ | ☒ Antibodies |
| ☒ | ☐ Eukaryotic cell lines |
| ☒ | ☐ Palaeontology and archaeology |
| ☒ | ☐ Animals and other organisms |
| ☐ | ☒ Clinical data |
| ☒ | ☐ Dual use research of concern |
| ☒ | ☐ Plants |

## Methods

| n/a | Involved in the study |
|---|---|
| ☒ | ☐ ChIP-seq |
| ☐ | ☒ Flow cytometry |
| ☒ | ☐ MRI-based neuroimaging |

## Antibodies

Antibodies used

ex vivo ICS assay: CD4 (BV421, clone: SK3, BD #566907, 2.5 uL/well), CD8 (BV786, clone: RPA-T8, BD #563823, 1:25), CD45RA (Alexa 700, clone: HI100, BioLegend #304120, 1:25), CCR7 (PE-CF594, clone: 15053, BD #562381, 1:12.5), Aqua Live/Dead marker (Thermo Fisher #L34966, 0.5 ul/well) and dump markers CD14 (PE-Cy5, clone: 61D3, Thermo Fisher #15-0149-42, 1:200), CD16 (PE-Cy5, clone: 3G8, BioLegend #302010, 1:200), and CD19 (PE-Cy5, clone: SJ25C1, BioLegend #363042, 1:100), CD3 (APC-H7, clone: SK7, BD #560176, 2.5 uL/well), IFNγ (FITC, clone: Mab11, BioLegend #506504, 1:200), TNFα (BV711, clone: B27, BioLegend #502940, 1:50), and IL2 (BV650, clone: MQ1-17H12, BioLegend # 500334, 1:50).

Extended ex vivo ICS assay: CD107a (Alexa Fluor 700, clone: H4A3, BD #561340, 1.25 ul/well), CD8 (BUV805, clone: SK1, BD #612889), CD45RA (PE-Cy7, clone: HI100, BD #560675), CCR7 (BUV615, clone: 3D12, BD #562381), Aqua Live/Dead marker (Thermo Fisher #L34966, 0.5 ul/well) and dump markers CD14 (PE-Cy5, clone: 61D3, Thermo Fisher #15-0149-42, 1:400), CD16 (PE-Cy5, clone: 3G8, BioLegend #302010, 1:100), and CD19 (PE-Cy5, clone: SJ25C1, BioLegend #363042, 1:100), CD3 (APC-H7, clone: SK7, BD #560176, 1:40), CD4 (BUV496, clone: SK3, BD #612936, 1:40), IFNγ (BB700, clone: B27, BD # 566394, 1:80), TNFα (BV750, clone: MAb11, BioLegend #502940, 1:80), IL2 (BV421, clone: MQ1-17H12, BD #564164, 1:40), Granzyme B (FITC, clone: GB11, BD #560211, 1:40), Perforin (PE, clone: B-D48, BioLegend #353304, 1;80), CD137 (BUV661, clone: 4B4-1, BD #741642, 1;80), CD154 (BUV563, clone: TRAP-1, BD #748984, 1:80), CD69 (BV711, clone: FN50, BD #563836, 1:160), Ki67 (BV650, clone: B56, BD #563757, 1;80), and FoxP3 (PE/Dazzle 594, clone: 206D, BioLegend #320126, 1:160).

Validation

The specificity of the antibodies purchased from commercial sources (BD Biosciences, BioLegend and Thermo Fisher) were validated by the manufacturer in house.
BD Bioscience:
https://www.bdbiosciences.com/en-us/products/reagents/flow-cytomtery-reagents/research-reagents/quality-and-reproducibility
1. The specificity is confirmed using multiple methodologies that may include a combination of flow cytometry, immunofluorescence, immunohistochemistry or western blot to test staining on a combination of primary cells, cell lines or transfectant models.
2. All flow cytometry reagents are titrated on the relevant positive or negative cells.
3. Quality control: Quality control testing of new, manufactured lots are performed side-by-side with a previously accepted lot as a control, helping to serve as a reference for comparison and assuring that performance of the new lot is both reliable and consistent.
4. Lot to lot consistency: Testing with prior batches as reference helps you obtain consistent results with the new batch relative to the previous batches.
BioLegend:
https://www.biolegend.com/en-us/quality/product development Flow cytometry reagents
Specificity testing of 1-3 target cell types with either single or multi-color analysis (including positive and negative cell types). Once specificity is confirmed, each new lot must perform with similar intensity to the in-date reference lot. Brightness (MFI) is evaluated from both positive and negative populations. Each lot product is validated by QC testing with a series of titration dilutions.
Thermo Fisher:
https://www.thermofisher.com/us/en/home/life-science/antibodies/invitrogen-antibody-validation/relative-expression-antibody-validation.html
1. Demonstration of primary antibody specificity by relative expression across cell models. Flow cytometry intrinsically facilitates the analysis of relative expression patterns in heterogeneous cell populations. Using gating techniques, verification of antibody binding can be determined by analyzing expression in unique cell types.
2. Independent validated antibodies. Utilizing two independent antibodies for the same protein target can be a useful tool when testing for antibody specificity. In the ideal scenario, two antibodies are used that target nonoverlapping epitopes of an antigen. By obtaining comparable results from antibodies that recognize independent regions of the same target protein, this allows for increased confidence that these antibodies are specific and suitable for the detection of their intended target. Independent antibody testing is one strategy we use to validate Invitrogen antibodies for research use. Common applications of independent antibody validation would be obtaining similar detection patterns in multi-lysate western blots, IHC arrays, immunofluorescence of multiple

cell lines, immunoprecipitation, flow cytometry, and other antibody applications.

## Clinical data

Policy information about clinical studies

All manuscripts should comply with the ICMJE guidelines for publication of clinical research and a completed CONSORT checklist must be included with all submissions.

| | |
|---|---|
| Clinical trial registration | Clinicaltrials.gov NCT04853017 |
| Study protocol | ELI-002-001 (AMPLIFY-201) |
| Data collection | The 8 enrolling academic sites were University of Colorado School of Medicine, City of Hope, Memorial Sloan Kettering Cancer Center, University of Texas MD Anderson, University of Iowa, Northwell Health, University of California Los Angeles, and Massachusetts General Hospital. Data were collected from 10 Oct 2021 through 24 September 2024 |
| Outcomes | Primary endpoints of the study were safety (adverse events were graded per Common Terminology Criteria for Adverse Events, version 5.0) and tolerability. The secondary outcomes of radiographic RFS defined as the time from first dose of ELI-002 2P until radiographic progression or death and OS defined as the time from first dose until death from any cause. |

## Flow Cytometry

### Plots

Confirm that:

☒ The axis labels state the marker and fluorochrome used (e.g. CD4-FITC).

☒ The axis scales are clearly visible. Include numbers along axes only for bottom left plot of group (a 'group' is an analysis of identical markers).

☒ All plots are contour plots with outliers or pseudocolor plots.

☒ A numerical value for number of cells or percentage (with statistics) is provided.

### Methodology

| | |
|---|---|
| Sample preparation | PBMCs were processed from leukapheresis using Ficoll-Hypaque gradients or whole blood collection using CPT tubes. PBMCs were cryopreserved and rested overnight before using. |
| Instrument | BD FACS Symphony |
| Software | Data was analyzed using FlowJo™ software. Graphs were compiled using GraphPad Prism. |
| Cell population abundance | No cell sorting was used for the analysis. |
| Gating strategy | First, a Live-Dead Aqua vs SSC-A gate to exclude non-viable cells, followed by a single cell gate FSC-H vs FSC-A to exclude doublets, then another single cell gate, FSC-A vs FSC-W to further exclude doublets, then a dump negative gate to exclude non T cells followed by FSC-A vs SSC-A to define lymphocytes and a CD3 vs SSC-A to gate on CD3+ T cells. CD4 vs CD8 gate was next used to separate CD8+ cells and CD4+ T cells. For ICS analysis cells, IFNg, TNFa and IL2 production was then assessed in CD8+ and CD4+ T cells using boolean gating. Memory populations were defined as CCR7 vs CD45RA. |

☒ Tick this box to confirm that a figure exemplifying the gating strategy is provided in the Supplementary Information.
