# Supplementary material for: Lymph node-targeted, mKRAS-specific amphiphile vaccine in pancreatic and colorectal cancer: phase 1 AMPLIFY-201 trial final results
Source: Nat Med. 2025 Aug 11;31(11):3648–53. doi: 10.1038/s41591-025-03876-4 (PMC12618236; doi:10.1038/s41591-025-03876-4)
Supplement: Supplementary file 3 — ELI-002-001 Protocol Version 8.0. [file 41591_2025_3876_MOESM3_ESM.pdf]

Protocol Number: ELI-002-001

Protocol Name: AMPLIFY-201

First in Human Phase 1 Trial of ELI-002 Immunotherapy as Treatment for Subjects with Kirsten Rat Sarcoma (KRAS) Mutated Pancreatic Ductal Adenocarcinoma and Other Solid Tumors

SPONSOR

Elicio Therapeutics, Inc.

Sponsor Contact: [redacted]
Phone: [redacted]
Email: [redacted]

Lead Medical Monitor: [redacted]
Phone: [redacted]
Email: [redacted]

Protocol Version: Version 8.0 Dated 07 August 2023

Supersedes: Version 7.0 Dated 25 January 2023

SIGNATURE PAGE

[redacted]	Date
[redacted]	Date
[redacted]	Date

INVESTIGATOR'S AGREEMENT

I have read and reviewed the ELI-002-001 Protocol, version 8.0, dated 07 August 2023, and agree to conduct the trial as outlined and with all applicable regulations including the current International Conference on Harmonization guidelines.

I agree to provide copies of the ELI-002-001 Protocol and the Investigator's Brochure for ELI-002, provided by the Sponsor (Elicio Therapeutics), to all members of the study team. I will ensure that all members of the study team are fully trained regarding the study drug and conduct of the study.

I agree to make available to Elicio Therapeutics, their representatives, and regulatory authorities, the study records of each subject in order to verify the data that Elicio Therapeutics has entered into the case report forms. I am aware of my responsibilities as a study investigator as provided by the Sponsor.

____

Printed Name of Investigator

____

Signature of Investigator

____

Date

PROCEDURES IN CASE OF EMERGENCY

Emergency contact information is provided in Table 1.

Table 1: Emergency Contact Information

Role in Trial	Name	Email and Telephone Number
Clinical Operations	[redacted]	[redacted]
Responsible Physician	[redacted]	[redacted]
24-Hour Emergency Contact	[redacted]	[redacted]

1. SYNOPSIS

**Name of Sponsor/Company:** Elicio Therapeutics, Inc. (hereafter, referred to as Elicio)		
**Name of Investigational Product:** ELI-002		
**Name of Active Ingredient:** ELI-002 is an 8-component product consisting of: up to 7 lipid-conjugated peptide-based antigens (amphiphilic peptides, referred to as 'Amph-Peptides') and a lipid-conjugated immune-stimulatory oligonucleotide ('Amph-CpG-7909') dosed after an admixing procedure of the respective Amph-Peptide and Amph-CpG-7909 drug products.<br>During the Phase 1 (dose escalation) study, the Amph-Peptide mixture (Amph-Peptides 2P) will be comprised of 1.4 mg of 2 Amph modified KRAS peptides, Amph-G12D, and Amph-G12R (0.7 mg/peptide). Elicio plans to investigate the use of an Amph-Peptide 7P drug product containing all 7 Amph-Peptides (G12D, G12R, G12V, G12A, G12C, G12S, G13D) in future clinical trials.		
**Protocol Number:** ELI-002-001	**Phase:** 1	**Country:** United States (of America)
**Title of Trial:** First in Human Phase 1 Trial of ELI-002 Immunotherapy as Treatment for Subjects with Kirsten Rat Sarcoma (KRAS) Mutated Pancreatic Ductal Adenocarcinoma (PDAC) and Other Solid Tumors		
**Trial centers:** Multicenter		
**Investigators:** Multicenter		
**Studied period (years):**<br>Date first subject enrolled: 4Q2021<br>Date last subject enrolled: 2Q2023		
**Hypothesis:**<br>ELI-002 is safe and the maximum tolerated dose (MTD) and/or recommended Phase 2 dose (RP2D) can be established at the end of the Phase 1 dose escalation.		
**Phase 1 Primary Objective:**<br>• To assess the safety and tolerability of ELI-002 as adjuvant therapy for subjects with KRAS mutated PDAC and other solid tumors who have minimal residual disease (MRD) identified using either circulating tumor DNA (ctDNA) or a serum tumor biomarker<br>• To define the MTD (in the event there is an MTD) and RP2D<br>**Phase 1 Secondary Objective:**<br>• To assess ctDNA reduction and clearance, defined as reduction or clearance in ctDNA compared to baseline, or if ctDNA was not detectable at baseline, serum tumor biomarker (such as carbohydrate antigen [CA]19-9, carcinoembryonic antigen [CEA], and CA-125) reduction and clearance compared to baseline<br>**Phase 1 Exploratory Objectives:**<br>• To report the median relapse-free survival (RFS) and median overall survival (OS)<br>• To assess the change relative to baseline in:		

- Serum cytokines interleukin (IL)-2, interferon gamma (IFNγ), IL-6, IL-10, and tumor necrosis factor alpha (TNFα)
- Patient reported outcomes (PROs): European Organization for Research and Treatment of Cancer (EORTC) Quality of Life Questionnaire [QLQ]-C30 and QLQ-PAN26, QLQ-CR29, QLQ-OV28, QLQ-BIL21, or QLQ-LC13
- To evaluate the immunogenicity of ELI-002. T cell responses to ELI-002 will be assessed using assays such as intracellular cytokine staining (ICS), Fluorospot, and/or dextramer+ T cells for subjects with human leukocyte antigen (HLA) alleles for which dextramer/s is/are available
- Biomarker levels (ctDNA, CA19-9, CEA, and CA-125)
- To evaluate tumor-infiltrating T cells and the tumor microenvironment after dosing with ELI-002

- To correlate biomarker data to clinical safety and efficacy (RFS, OS)

Primary Endpoints:

- To define the MTD of ELI-002 (in the event there is an MTD) and the RP2D
- To evaluate safety as assessed by the incidence of adverse events (AEs) and clinically significant changes in laboratory tests and vital signs

Secondary Endpoint:

- To report the proportion of subjects with ctDNA reduction and clearance, defined as reduction or clearance in ctDNA compared to baseline, or if ctDNA was not detectable at baseline, serum tumor biomarker (such as CA19-9, CEA, and CA-125) reduction and clearance compared to baseline

Exploratory Endpoints:

- Median RFS and median OS
- Duration of biomarker (ctDNA or serum tumor biomarker) clearance and reduction, defined as time from the date of the first negative and/or decreased biomarker to the earliest date of any of the following events:
  - Subsequent increased/positive biomarker
  - Subsequent disease relapse
  - Death due to any cause
- Change relative to baseline in:
  - Serum cytokines IL-2, IFNγ, IL-6, IL-10, and TNFα
  - PROs: EORTC QLQ-C30 and QLQ-PAN26, QLQ-CR29, QLQ-OV28, QLQ-BIL21 or QLQ-LC13
  - Immunogenicity of ELI-002 determined by magnitude of response and fold-change from baseline, using assays such as ICS, Fluorospot, and/or dextramer+ T cells for subjects with HLA alleles for which dextramer/s is/are available
  - Biomarker levels (ctDNA, CA19-9, CEA, and CA-125)
  - Immune cell infiltrate and tumor microenvironment if standard of care biopsies are obtained to confirm disease progression

Trial Design:

ELI-002-001 is an open-label, Phase 1 trial of ELI-002 immunotherapy as adjuvant treatment for subjects with KRAS/neuroblastoma ras viral oncogene homolog (NRAS) mutated PDAC and other solid tumors who are at high risk for relapse (ie, presence of isolated tumor cells as detected by ctDNA or elevated serum tumor biomarkers in the subject's body, in which the primary tumor has been removed and is currently without clinical signs of disease). For this protocol, high relapse risk is defined by either a ctDNA-positive result, or elevated serum tumor biomarker (such as CA19-9, CEA, or CA-125).

This Phase 1 (dose escalation) trial comprises an open-label, single arm, dose-escalation trial incorporating a 3+3 design in subjects with mutant KRAS (mKRAS)/mutant NRAS (mNRAS) solid tumors (PDAC, CRC, non-small cell lung cancer [NSCLC], ovarian, cholangiocarcinoma [CCA] and gallbladder carcinoma).

Approximately 18 subjects are planned to be enrolled in the dose escalation to evaluate the safety, tolerability, and exploratory pharmacodynamic efficacy of subcutaneous (SC) ELI-002 using the adjuvant at 3 planned dose levels (Amph-CpG-7909 0.1 mg SC, 0.5 mg SC, 2.5 mg SC) in combination with a fixed dose of Amph-Peptides (700 µg each). Additional cohorts may be added to explore intermediate or higher dose levels based on the cumulative safety review and preliminary review of pharmacodynamic responses. If additional cohorts are added, this will be documented in a study memo sent to all clinical sites and submitted to IRBs, per IRB guidance.

During the dose escalation study, ELI-002 will be comprised of 1.4 mg of two Amph modified KRAS peptides G12D and G12R admixed with Amph-CpG-7909 to provide ELI-002 2P. Elicio plans to investigate the use of an Amph-Peptide 7P drug product containing all 7 Amph-Peptides (G12D, G12R, G12V, G12A, G12C, G12S, G13D) admixed with Amph-CpG-7909 (ELI-002 7P) in future clinical trials.

Subjects in Phase 1 will receive the initial dose of ELI-002 2P and be monitored for ≥24 hours in order to assess for potential AEs such as cytokine release syndrome (CRS). In addition, there will also be a 1-week stagger between enrollment of the first subject in a cohort and the subsequent subjects. In the absence of toxicity during the dose-limiting toxicity (DLT) assessment window, the ELI-002 2P dose will be escalated. Planned cohort dose levels are listed below:

- Cohort 1: Dose Level 1: Amph-CpG-7909 0.1 mg with Amph-Peptides
- Cohort 2: Dose Level 2: Amph-CpG-7909 0.5 mg with Amph-Peptides
- Cohort 3: Dose Level 3: Amph-CpG-7909 2.5 mg with Amph-Peptides

Cohorts will consist of 3 to 6 subjects (the number will depend on whether DLTs are observed). In order to ensure an adequate number of evaluable subjects, up to 6 subjects may be enrolled per cohort even in the absence of a DLT. Once the RP2D is determined, the cohort evaluating the Phase 2 dose will be expanded as needed so that there is safety experience with at least 6 subjects at this dose level.

Dose escalation from one cohort to the next will be determined by the Safety Monitoring Committee (SMC), and will be based on treatment-emergent AEs, clinical laboratory data, physical examination (PE) findings including vital signs, after all subjects within a cohort have completed 28 days. At each SMC meeting, longer follow-up will be available on all but the final subject in each cohort. The SMC will review all available long-term safety information from prior cohorts (if any), so that the SMC will consider the totality of the safety information in their recommendation.

In any cohort, if none of the subjects experience a DLT during the first 28 days following first administration of ELI-002, dose escalation will occur, and 3 subjects will be enrolled in the cohort at the next dose level. However, if 1 of the 3 or 4 initial subjects in a cohort experiences a DLT, then 3 (or 2, if 4 were originally enrolled) additional subjects will be enrolled at the same dose level. If 2 or more of 3 subjects within a cohort experience a DLT, then this dose will be considered the toxic

dose. If only 1 of 6 subjects within a cohort experiences a DLT during the first 28 days of treatment, then the next cohort may begin enrollment. If 2 or more of 6 subjects within a cohort experience a DLT during the first 28 days, then this dose will be considered the toxic dose. The trial may proceed at a lower dose, at a less frequent schedule, or omitting the Amph-CpG-7909 adjuvant while continuing to administer the Amph-Peptides, based on emerging toxicity or pharmacodynamic data until the MTD is determined.

A trial memo will be sent to all sites following each SMC cohort review meeting summarizing the decision; if additional cohorts are to be enrolled, the dose level and schedule will be provided.

The RP2D will be defined in consideration of the MTD (if any), safety data, and pharmacodynamic data. The RP2D will be communicated to all sites in a trial memo following the last SMC cohort review.

Subject replacement: If a subject discontinues for any reason other than toxicity prior to completing 28 days of ELI-002 therapy, a replacement subject will be added to the cohort until 3 subjects per cohort complete 28 days of ELI-002 therapy.

DLT definition: A DLT will be defined as any event at least possibly related to ELI-002 as follows:

a. Any Grade ≥ 3 non-hematologic toxicity except asymptomatic laboratory abnormalities (see Section 12.2.1.1)
b. Any Grade ≥ 3 toxicity involving major organ systems for greater than 72 hours and occurring within 28 days of subcutaneous administration
c. Grade 3 CRS that does not resolve to ≤ Grade 2 within 7 days
d. Any Grade 4 CRS that does not improve to < Grade 2 within 72 hours
e. Any Grade ≥ 3 autoimmune disorder
f. Any Grade ≥ 3 rash that does not resolve to ≤ Grade 1 within 7 days with appropriate treatment

The DLT observation period will be 28 days.

MTD definition: The MTD is defined as the highest dose level with a DLT incidence <33% of cohort subjects.

The trial will consist of a Screening Period, an Immunization Period, a No Dosing 3-Month Period, a Booster Period, and a Follow-up Period.

A Booster Period is provided as further lymph node antigen exposure may be required to maintain effective levels of antigen-specific T-cells. All subjects will receive booster doses unless they have unresolved toxicity.

All subjects will be observed for safety and tolerability. CT imaging with contrast will occur at screening and 35 days post first dose and at additional timepoints until radiographic relapse is observed.

The central laboratory will confirm ≥1 of 2 mutant KRAS/NRAS alleles targeted by ELI-002 Amph-Peptides 2P (G12D, and G12R,) is present and central laboratory ctDNA must be positive or there must be serum tumor biomarker (such as CA19-9, CEA, and CA-125) elevation at ≥21 days post-surgery or after last administration of adjuvant therapy (whichever comes last in the specific subject treatment plan). These data are required for determination of eligibility and inclusion into the trial.

Immunologic biomarkers of response may include HLA typing, serum cytokines, ICS, Fluorospot, dextramer+ T cells for subjects with appropriate HLA alleles will be determined when matching dextramer/s is/are available.

Local laboratories will be utilized for serum cytokine testing, tumor biomarkers CA19-9/CEA/CA-125 (as appropriate per tumor type), tumor biopsy assays (such as biomarker sequencing and/or IHC assays) and all other safety laboratory tests.

Central laboratories will be utilized for ctDNA analysis, HLA typing, peripheral blood mononuclear cell (PBMC) isolation and immunogenicity assessment (such as Fluorospot, ICS, and/or T cell dextramer testing) as detailed in the central laboratory manual.

While HLA testing will be performed prior to first dose at Visit 3, cohorts will be enrolled regardless of the HLA subtype.

Discontinuation of Trial Treatment: Subjects with confirmed radiographic relapse (using iRECIST criteria; not judged as pseudoprogression) during treatment with ELI-002 will discontinue trial treatment but should continue other trial-related procedures in the follow-up period.

The design of the Phase 1 trial is outlined in the following figure.

ELI-002 Study Cohort Schematic

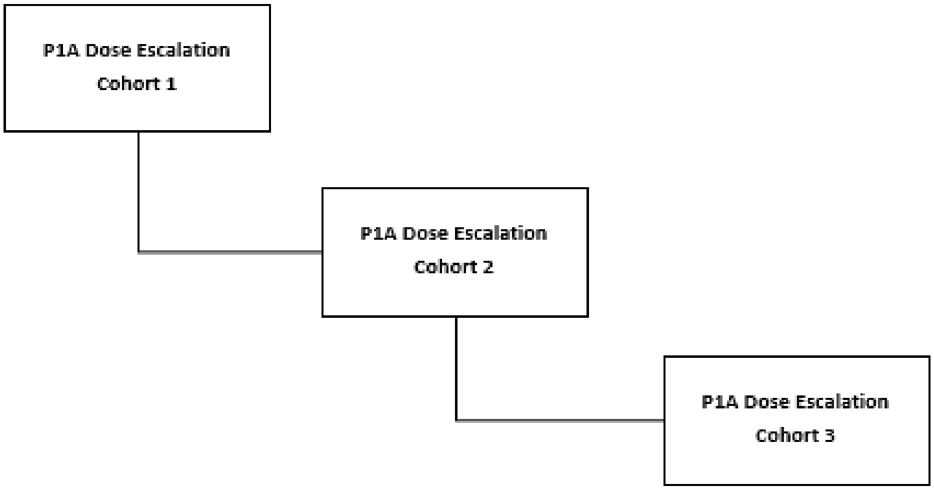

All cohorts are open to enrollment of subjects with RAS mutated PDAC, CRC, NSCLC, and other solid tumors (OST; including ovarian, bile duct and gallbladder carcinoma).

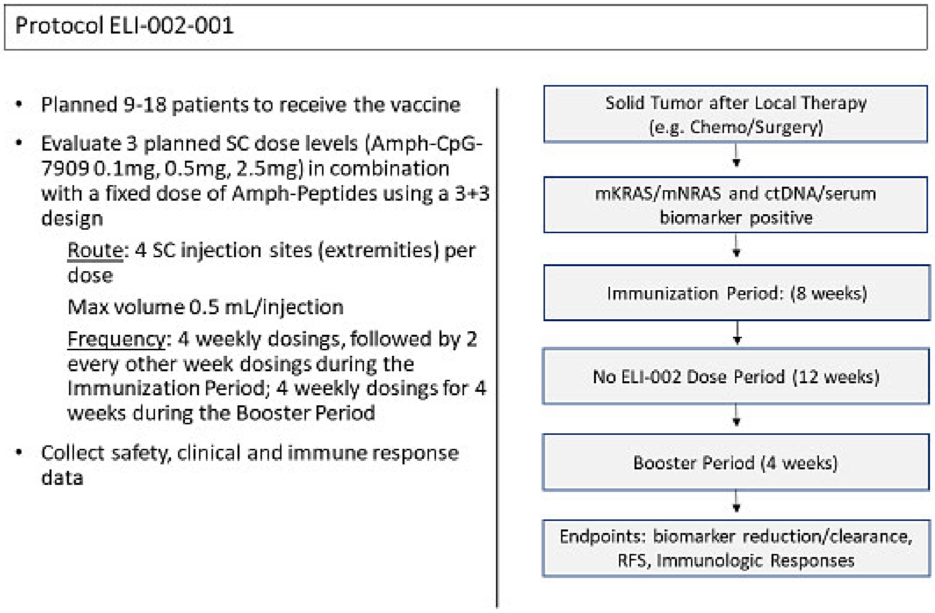

Sample Size Justification: The sample size for the Phase 1 dose escalation is not based on power calculations, but rather on clinical judgement and the expectation that the objectives of this part of the trial will be met with approximately 18 subjects enrolled in the 3+3 design.

Number of Subjects (planned): In the Phase 1 Dose Escalation approximately 18 subjects are planned to enroll: 3 to 6 subjects in Cohort 1; 3 to 6 subjects in Cohort 2; and 3 to 6 subjects in Cohort 3.

Diagnosis and Main Criteria for Inclusion: Males or females ≥18 years of age with histologically or cytologically confirmed diagnosis of solid tumor with central laboratory analysis confirming the presence of at least 1 of the 2 KRAS/NRAS mutations targeted by ELI-002 Amph-Peptides 2P.

Investigational Product, Dosage and Mode of Administration:

ELI-002 is an investigational product consisting of 2 lipid-conjugated peptide (G12R and G12D) based antigens (amphiphilic peptides, referred to as 'Amph-Peptides') and a lipid-conjugated immune-stimulatory oligonucleotide ('Amph-CpG-7909') dosed after an admixing procedure of the respective Amph-Peptides 2P and Amph-CpG-7909 drug products (referred to as ELI-002 in this protocol). In the event of toxicity after immunization doses, Amph-Peptides without Amph-CpG-7909 adjuvant may be administered during the booster period. Elicio plans to investigate the use of an Amph-Peptide 7P drug product containing all 7 Amph-Peptides (G12D, G12R, G12V, G12A, G12C, G12S, G13D) admixed with Amph-CpG-7909 (ELI-002 7P) in future clinical trials.

The doses planned to be evaluated in Phase 1 are as shown below:

Phase	Cohort	Tumor	Dose Level
**Phase 1 Dose Escalation**	Cohort 1	KRAS/NRAS mutated solid tumors	Amph-CpG-7909 0.1 mg with Amph-Peptides
	Cohort 2	KRAS/NRAS mutated solid tumors	Amph-CpG-7909 0.5 mg with Amph-Peptides
	Cohort 3	KRAS/NRAS mutated solid tumors	Amph-CpG-7909 2.5 mg with Amph-Peptides

CRC=colorectal cancer; KRAS=Kirsten Rat Sarcoma; NRAS=neuroblastoma ras viral oncogene homolog; NSCLC=non-small cell lung cancer; OST=other solid tumors; PDAC=pancreatic ductal adenocarcinoma

Duration of Treatment: The total duration of participation for each subject is approximately 2 years, and includes a Screening Period, an Immunization Period, a No Dosing 3-Month Period, a Booster Period, and a Follow-up Period.

Subjects who remain in the trial until the end of the required Follow-up Period may be eligible to enroll in an Extension Protocol.

Reference Therapy, Dosage and Mode of Administration: None

Criteria for Evaluation:

Safety: Adverse events will be recorded and categorized using CTCAE, Version 5. If observed, CRS/ICANS will be recorded and managed according to the American Society for Transplantation and Cellular Therapy (ASTCT) consensus criteria.

Standard clinical chemistry and hematology assays, PEs, vital sign measurements, electrocardiograms, and ECOG performance status will be assessed/performed at specified times during the trial.

A SMC comprised of the site investigators, sponsor medical monitor, drug safety and clinical operations, will meet for each dose escalation. The SMC will have the authority to assess and

recommend any dosing changes warranted due to safety concerns or as otherwise warranted to protect the safety of the subjects in the trial.<br>**Efficacy:** Median RFS and 1-year RFS rate will be assessed through imaging using iRECIST criteria; subjects will be followed for OS. In addition, biomarker (ctDNA or serum tumor biomarker) response rate will be based on the percentage of subjects achieving biomarker reduction or clearance, as measured by the reduction or absence of the biomarker, and the duration of biomarker reduction and/or clearance will be recorded.<br>**Immunogenicity:** Patients will have leukapheresis and blood samples drawn to evaluate the immunogenicity of ELI-002. T cell responses in PBMCs will be assessed by immune assays such as IFNγ, Fluorospot, ICS, and/or dextramers. Serum cytokine, CEA, CA-125 and CA 19-9 levels will also be examined. Immune responses will be correlated to clinical efficacy parameters.<br>**Standard of care tumor biopsies:** may be collected post-dose per iRECIST, and if there is sufficient tumor tissue collected, will be assayed at the local laboratory to examine tumor-infiltrating T cells and the tumor microenvironment. Potential assays could include in situ gene expression and/or IHC.
**Statistical Methods:** Descriptive statistics will be generated to summarize demographic, medical history, and safety data.<br>As a general approach, continuous variables will be summarized using mean, standard deviation, median, minimum value, maximum value. Categorical variables will be summarized using frequency counts and percentages. Time to event data will be summarized using the Kaplan-Meier method. Where appropriate, confidence intervals around point estimates will be presented, and estimates of the median and other quantiles, as well as individual time points (for time to event data) will be produced.<br>All statistical hypothesis testing within (for change from baseline) and between dose cohorts will be conducted at the 1-sided 0.05 level of significance. Nominal p-values may be computed for secondary or exploratory efficacy analyses as a descriptive measure of strength of results rather than for formal tests of hypotheses. Emphasis will be on RP2D, highest dose, and combined RP2D and all higher doses for change from baseline and comparison to lowest dose. No formal tests of hypotheses are planned for safety analyses.<br>Immunogenicity analyses: A positive immune response for each KRAS/NRAS peptide will be determined, for example, using a non-parametric distribution-free resampling method with the Fluorospot assay. T cell responses to ELI-002 will be summarized for each patient by magnitude of response and fold-change from baseline for each timepoint tested. Immune responses will be summarized for each Cohort.

2. TABLE OF CONTENTS, LIST OF TABLES, AND LIST OF FIGURES

TABLE OF CONTENTS

TITLE PAGE 1
INVESTIGATOR'S AGREEMENT 3
PROCEDURES IN CASE OF EMERGENCY 4
1. SYNOPSIS 5
2. TABLE OF CONTENTS, LIST OF TABLES, AND LIST OF FIGURES 12
3. LIST OF ABBREVIATIONS AND DEFINITIONS OF TERMS 19
4. INTRODUCTION 23
4.1. Summary of Pancreatic Cancer and Other Solid Tumors 25
4.1.1. Pancreatic Cancer 25
4.1.2. Colorectal Cancer 29
4.1.2.1. Introduction 29
4.1.2.2. Adjuvant Therapy in the Case of Localized CRC Disease 29
4.1.2.3. Rectal Cancer 30
4.1.2.4. Colorectal Liver Metastases 30
4.1.2.5. Frequency of KRAS and NRAS Mutations in CRC 31
4.1.2.6. Importance of ctDNA and CEA Assessment and Follow-up in CRC 32
4.1.2.7. Colorectal Cancer and Vaccines 33
4.1.3. Non-small Cell Lung Cancer 34
4.1.3.1. KRAS Mutations in NSCLC 34
4.1.3.2. Circulating Tumor DNA in NSCLC 35
4.1.4. Ovarian Carcinoma 35
4.1.4.1. Ovarian KRAS Mutations 36
4.1.4.2. Circulating Tumor DNA in Ovarian Cancer 37
4.1.5. Cholangiocarcinoma 37
4.1.5.1. Cholangiocarcinoma KRAS Mutations 38
4.1.6. Gallbladder Cancer 39
4.2. Brief Summary of Nonclinical Pharmacology 39
4.3. Brief Summary of Pharmacokinetics and Toxicokinetics 46
4.4. Brief Summary of Toxicology 46

4.4.1. Repeat-Dose Toxicity Studies 46
4.4.1.1. Study No. 2795-001: Amph-CpG-7909 (with and without Amph-Peptide-G12D): A 4-Week Subcutaneous Injection Tolerability Study in Male C57BL/6 Mice 46
4.4.1.2. Study 2795-002: A GLP 3-Month Study of ELI-002 by Subcutaneous Injection in C57BL/6 Mice with a 5-Week Recovery Period....47
4.5. Safety Pharmacology 47
4.6. ELI-002: An Investigational Treatment for KRAS/NRAS Mutated Solid Tumors....48
4.6.1. Mechanism of Action 48
4.6.2. Amphiphile Lymph Node Delivery Background 48
4.6.3. Known and Potential Risks and Benefits....49
4.6.4. Trial Rationale 51
4.6.5. Rationale for Endpoints 53
4.6.6. Rationale for Dose Selection 54
5. TRIAL OBJECTIVES 55
5.1. Primary Objectives 55
5.2. Secondary Objective 55
5.3. Exploratory Objectives 55
6. INVESTIGATIONAL PLAN....56
6.1. Overall Trial Design 56
6.1.1. Safety Monitoring....59
6.1.2. Criteria for Booster and Additional Booster Vaccines 60
6.1.3. Screening for Human Leukocyte Antigen Subtypes 60
6.1.4. Schedule of Assessments....60
6.1.5. Risk Determination for Investigational In Vitro Diagnostics....72
6.1.5.1. Description of the IVDs....73
6.1.5.2. Applying the IVD Results in the Clinical Trial 75
6.2. Endpoints 75
6.2.1. Primary Endpoints 75
6.2.2. Secondary Endpoint....75
6.2.3. Exploratory Endpoints....75
6.3. Number of Subjects 76

6.4. Treatment Assignment 76
6.5. Dose Adjustment Criteria 76
6.6. Safety Criteria for Temporarily or Permanently Stopping the Trial 76
6.7. Management of Study Drug-Related Events 77
6.7.1. Temporarily Suspending Treatment Administration 77
6.7.2. Permanent Stopping Treatment Administration 77
6.7.3. Dose-limiting Toxicity Guidelines 78
6.8. Management of Risks and Toxicities 78
6.8.1. Management of Cytokine Release Syndrome 79
6.8.2. Cytokine Release Syndrome Treatment Guidelines 81
6.8.3. ICANS Treatment Guidelines 83
6.8.4. Management of On-target Off-tumor Toxicity 85
6.8.5. Management of Maculo-Papular or Acneiform Rashes, Injection Site Reactions 85
7. SELECTION AND WITHDRAWAL OF SUBJECTS 86
7.1. Subject Inclusion Criteria 86
7.2. Subject Exclusion Criteria 89
7.3. Subject Withdrawal Criteria 90
7.3.1. Subject Replacement 91
7.3.2. Subjects Lost to Follow-up 91
8. TREATMENT OF SUBJECTS 92
8.1. Concomitant Medications 92
8.1.1. Prohibited Concomitant Medications 92
8.2. Treatment Compliance 92
8.3. Randomization and Blinding 92
9. TRIAL DRUG MATERIALS AND MANAGEMENT 93
9.1. Trial Drug 93
9.2. Trial Drug Packaging and Labeling 93
9.3. Trial Drug Storage 93
9.4. Trial Drug Preparation 93
9.5. Administration and Dosage 94
9.6. Trial Drug Accountability 94
9.7. Trial Drug Handling and Disposal 94

10. TIMING OF TRIAL PROCEDURES 95
10.1. Screening and Observation Period 95
10.1.1. Rescreening 96
10.2. Immunization Period 97
10.3. No Dosing 3-Month Period 101
10.4. Booster Period 102
10.5. Follow-up Period 106
11. CLINICAL ACTIVITY, IMMUNE ASSESSMENTS, AND SUBJECT REPORTED OUTCOMES 108
11.1. Immunogenicity 108
11.2. Tumor Tissue Collection at Surgery 109
11.3. Patient Reported Outcomes 109
11.3.1. EORTC QLQ-C30 109
11.3.2. Tumor type-specific PROs 109
11.4. ctDNA Levels 110
12. ASSESSMENT OF SAFETY 111
12.1. Safety Parameters 111
12.1.1. Demographics and Medical History 111
12.1.2. Vital Signs 111
12.1.3. Physical Examinations 111
12.1.4. Electrocardiogram 111
12.1.5. Eastern Cooperative Oncology Group 111
12.1.6. Laboratory Assessments 111
12.1.6.1. Hematology 112
12.1.6.2. Coagulation 112
12.1.6.3. Blood Chemistry 112
12.1.6.4. Serum Cytokines 113
12.1.6.5. Viral Laboratory Testing at Screening 113
12.1.6.6. Pregnancy Screen 113
12.2. Adverse and Serious Adverse Events 113
12.2.1. Definition of Adverse Events 113
12.2.1.1. Adverse Event 113
12.2.1.2. Serious Adverse Event 114

12.2.1.3. Reactogenicity to the Vaccine 114
12.2.1.4. Cytokine Release Syndrome 115
12.3. Relationship to Trial Drug 115
12.4. Severity of Adverse Events 116
12.5. Recording Adverse Events 116
12.6. Pregnancy 117
12.7. Reporting Adverse Events 117
12.8. Extension Protocol 118
13. STATISTICS 119
13.1. Sample Size 119
13.2. General Statistical Considerations and Definitions 119
13.2.1. General Statistical Methods 119
13.2.2. Analysis Population 120
13.2.2.1. Full Analysis Set (FAS) 120
13.2.2.2. Safety Analysis Set 120
13.2.2.3. Disease Response Evaluable Set 120
13.2.2.4. Per Protocol Set 121
13.2.2.5. PD/Biomarker Evaluable Set 121
13.2.3. Missing Data 121
13.3. Statistical Analyses 121
13.3.1. Demographic and Background Characteristics 121
13.3.2. Immunogenicity Analysis 121
13.3.3. Efficacy Analysis 121
13.3.3.1. Relapse-free survival 121
13.3.3.2. Analysis of Biomarker Response 122
13.3.3.3. Analysis of Duration of Biomarker Response 122
13.3.3.4. Analysis of 1-Year RFS 122
13.3.3.5. Analysis of OS 122
13.3.4. Safety Analysis 123
13.3.5. Pharmacokinetics 123
13.3.6. Pharmacodynamics 123
14. DATA INTEGRITY AND QUALITY ASSURANCE 124

14.1. Data Management and Case Report Forms 124
14.2. Source Documentation....124
14.3. Study Drug Accountability....124
14.4. Laboratory Assessments....125
14.5. Trial Monitoring....125
14.6. Protocol Deviations....125
14.7. Audits and Inspections....125
14.8. Institutional Review Board and Other Committees....126
14.8.1. Institutional Review Board....126
14.8.2. Safety Monitoring Committees....126
15. ETHICS....127
15.1. Ethical Conduct of the Trial....127
15.2. Written Informed Consent....127
16. DATA HANDLING AND RECORDKEEPING....128
16.1. Retention of Records....128
17. PUBLICATION POLICY....129
18. LIST OF REFERENCES....130
19. APPENDICES....141
19.1. ECOG Performance Status Scale....141

LIST OF TABLES

Table 1: Emergency Contact Information....4
Table 2: Abbreviations and Specialist Terms....19
Table 3: Peptide Elements of Amph-Peptides....24
Table 4: KRAS Mutation Spectrum in Pancreatic Cancer....28
Table 5: Colon and Rectal Cancer KRAS and NRAS Mutation Spectrum....31
Table 6: KRAS Mutation Spectrum in Non-small Cell Lung Cancer....34
Table 7: KRAS Mutation Spectrum in Ovarian Tumors....36
Table 8: KRAS Mutation Frequency in Ovarian Tumors....37
Table 9: KRAS Mutation Spectrum in Cholangiocarcinoma....38
Table 10: Nonclinical Pharmacology studies of mKRAS Vaccine in Support of ELI-002 (Model = Female C57BL/6 Mice, 5, 10 or 15/group)....41

Table 11: Schedule of Assessments- Screening and Observation 61
Table 12: Schedule of Assessments- Immunization Period 63
Table 13: Schedule of Assessments- No Dosing 3-Month Period 65
Table 14: Schedule of Assessments- Booster Period 67
Table 15: Schedule of Assessments- Follow-up Period (Year 1 to Year 2) 69
Table 16: Schedule of Assessments- Follow-up Period (Year 2 to Year 3) 71
Table 17: Clinical Symptoms of Cytokine Release Syndrome 80
Table 18: Suggested Treatment by Cytokine Release Syndrome Grade 81
Table 19: High-dose Vasopressors (All Doses are Required for ≥3 Hours) 81
Table 20: Encephalopathy Assessment Tool for Grading ICANS 83
Table 21: ASTCT ICANS Consensus Grading for Adults 84
Table 22: Neurotoxicity Suggested Treatment 85
Table 23: Phase 1 Cohorts and Doses 94
Table 24: ECOG Performance Status Scale 141

LIST OF FIGURES

Figure 1: Structure of Amph-CpG-7909 24
Figure 2: ELI-002 Trial Cohort Schematic 58
Figure 3: Protocol ELI-002-001: Phase 1 Trial Design 58
Figure 4: Flow Diagram of ELI-002-001 Eligibility Determination using the IVD assays 73

3. LIST OF ABBREVIATIONS AND DEFINITIONS OF TERMS

The following abbreviations and specialist terms are used in this trial protocol.

Table 2: Abbreviations and Specialist Terms

Abbreviation or Specialist Term	Explanation
ADL	Activities of daily life
AE	Adverse event
AIDS	Acquired immune deficiency syndrome
AJCC	American Joint Committee on Cancer
ALT	Alanine aminotransferase
APC	Antigen-presenting cell
AST	Aspartate aminotransferase
ASTCT	American Society for Transplantation and Cellular Therapy
BUN	Blood urea nitrogen
CA	Carbohydrate antigen (cancer antigen)
CAP	College of American Pathologist
CAR	Chimeric antigen receptor
CBC	Complete blood count
CCA	Cholangiocarcinoma
CEA	Carcinoembryonic antigen
CGP	Comprehensive Genomic Profiling
CI	Confidence interval
CLIA	Clinical Laboratory Improvement Amendment
CRC	Colorectal cancer
CRP	C-reactive protein
CRS	Cytokine release syndrome
CSF	Cerebrospinal fluid
CT	Computed tomography
CTCAE	Common Terminology Criteria for Adverse Events, Version 5
ctDNA	Circulating tumor deoxyribonucleic acid
Da	Daltons
dCCA	Distal cholangiocarcinoma
DLT	Dose-limiting toxicity

Abbreviation or Specialist Term	Explanation
DMC	Data Monitoring Committee
DFS	Disease-free survival
EDTA	Ethylenediaminetetraacetic acid
EEG	Electroencephalogram
ECG	Electrocardiogram
ECOG	Eastern Cooperative Oncology Group
eCRF	Electronic Case Report Form
EORTC	European Organization for Research and Treatment of Cancer
FAS	Full Analysis Set
FDA	Food and Drug Administration
5-FU	5-fluorouracil
GCP	Good Clinical Practice
GLP	Good Laboratory Practices
GM-CSF	Granulocyte-macrophage colony-stimulating factor
GMP	Good Manufacturing Practices
HbsAG	Hepatitis B virus surface antigen
HCV	Hepatitis C virus
HIV	Human immunodeficiency virus
HLA	Human leukocyte antigen
HR	Hazard ratio
ICANS	Immune effector cell-associated neurotoxicity syndrome
iCCA	Intrahepatic cholangiocarcinoma
ICE	Immune Effector Cell-Associated Encephalopathy
ICH	International Conference on Harmonization
ICS	Intracellular cytokine staining
IFNγ	Interferon gamma
IL	Interleukin
IND	Investigational New Drug
INR	International normalized ratio
IRAE	Immune related adverse event
IRB	Institutional Review Board

Abbreviation or Specialist Term	Explanation
iRECIST	Immune Response Evaluation Criteria in Solid Tumors
IV	Intravenous(ly)
IVD	In vitro diagnostic
KRAS	Kirsten rat sarcoma
LDH	Lactate dehydrogenase
MHC	Major histocompatibility complex
mKRAS	Mutant Kirsten rat sarcoma
mNRAS	Mutant neuroblastoma ras viral oncogene homolog
MRD	Minimal residual disease
MRI	Magnetic resonance imaging
MSI	Microsatellite instability
MTD	Maximum tolerated dose
MW	Molecular weight
NCCN	National Comprehensive Cancer Network
NED	No evidence of disease
NGS	Next-generation sequencing
NOAEL	No-observed-adverse-effect level
NRAS	Neuroblastoma ras viral oncogene homolog
NSCLC	Non-small cell lung cancer
ORR	Objective response rate
OS	Overall survival
PBMC	Peripheral blood mononuclear cell
pCCA	Perihilar cholangiocarcinoma
PCR	Polymerase chain reaction
PDAC	Pancreatic ductal adenocarcinoma
PE	Physical examination
PK	Pharmacokinetics
PI	Primary Investigator
Poly IC	Polyinosinic:polycytidylic acid
PRO	Patient reported outcome
PT	Prothrombin time

Abbreviation or Specialist Term	Explanation
PTT	Partial thromboplastin time
QLQ	Quality of life questionnaire
QoL	Quality of Life
ROA	Route of administration
RP2D	Recommended Phase 2 dose
RFS	Relapse-free survival
SAE	Serious adverse event
SARS	Severe acute respiratory syndrome
SC	Subcutaneous
SEER	Surveillance, Epidemiology, and End Results
SMC	Safety Monitoring Committee
SOC	Standard of care
SUSAR	Suspected Unexpected Serious Adverse Reaction
TEN	Toxic epidermal necrolysis
TNFα	Tumor necrosis factor alpha
TLR-9	Toll-like-receptor-9
ULN	Upper limit of normal
US	United States (of America)
WES	Whole exome sequencing
WFI	Water for injection
WT	Wild type
XRT	Radiation therapy

4. INTRODUCTION

ELI-002 is a structurally novel amphiphilic therapeutic vaccine being studied as post-surgical adjuvant treatment of minimal residual disease (MRD) in subjects with Kirsten rat sarcoma (KRAS) viral oncogene homolog mutated pancreatic ductal adenocarcinoma (PDAC) and other KRAS mutated solid tumors.

ELI-002 uses amphiphile lymph node targeting which was developed at the Massachusetts Institute of Technology and licensed to Elicio Therapeutics. In cancer subjects, visual identification of sentinel lymph nodes during surgery is accomplished by injections of dyes that bind to endogenous albumin, targeting these compounds to draining lymph nodes. Amphiphiles were developed as an approach to improve the potency and safety of vaccination by harnessing the ability of the lipid component to bind to albumin and deliver a payload to lymph node antigen-presenting cells (APCs) [Liu et al., 2014]. Initial studies showed that amphiphiles markedly increase lymph node accumulation and decrease systemic dissemination relative to their parent soluble compounds, leading to 30-fold increases in T-cell priming and enhanced anti-tumor efficacy with reduced systemic toxicity. Subsequently investigation of the mechanism of action of amphiphiles using Batf3 knockout mice determined that the pharmacology requires cross-presenting dendritic cells in lymph nodes, and that combinations of amphiphiles with tumor-antigen-targeting antibodies, cytokines, and checkpoint inhibition using PD1 antibodies were able to destroy large established transplanted and genetically induced tumors in mice curing a majority of cancers in experimental settings previously considered intractable [Moynihan et al., 2016]. Most recently, amphiphile combinations with chimeric antigen receptor (CAR) T cells demonstrated potent enhancement of their activity including modulation of their phenotype to increase the proportion of central memory T cells, to extend the longevity of these cells, and to confer the ability to induce antigen spreading, which together led to cures of the majority of tumors in the models studied [Ma et al., 2019; Singh and June, 2019].

For the first clinical translation of the amphiphile technology, Elicio is developing ELI-002, a mutant KRAS (mKRAS)/mutant neuroblastoma ras viral oncogene homolog (mNRAS) lymph node targeting immuno-oncology product candidate. ELI-002 is comprised of up to 7 lipid-conjugated peptide-based antigens (‘Amph-Peptides’) described in Table 3, and a lipid-conjugated immune-stimulatory oligonucleotide (‘Amph-CpG-7909’) depicted in Figure 1.

Table 3: Peptide Elements of Amph-Peptides

Peptide Name	Peptide Sequence[a]	Peptide MW (Da)[b]	Residues[b]	Amph-Peptide MW (Da)[b]
G12D 4-21	CYKLVVVGADGVGKSALTI	1790	18	5049
G12V 4-21	CYKLVVVGAVGVGKSALTI	1774	18	5033
G12R 4-21	CYKLVVVGARGVGKSALTI	1831	18	5090
G12A 4-21	CYKLVVVGAAGVGKSALTI	1746	18	5005
G12S 4-21	CYKLVVVGASGVGKSALTI	1762	18	5021
G12C 4-21	YKLVVVGACGVGKSALTI	1778	18	4934
G13D 4-21	CYKLVVVGAGDVGKSALTI	1790	18	5049

[a] Variable residues: aspartic acid (DG and GD); valine (V); arginine (R); alanine (A); serine (S); cysteine (C)
[b] Peptide MW and Residues exclude *N*-terminal cysteine (C) required for Amphiphile sulfhydryl-maleimide coupling; Da=Daltons; MW=molecular weight

Figure 1: Structure of Amph-CpG-7909

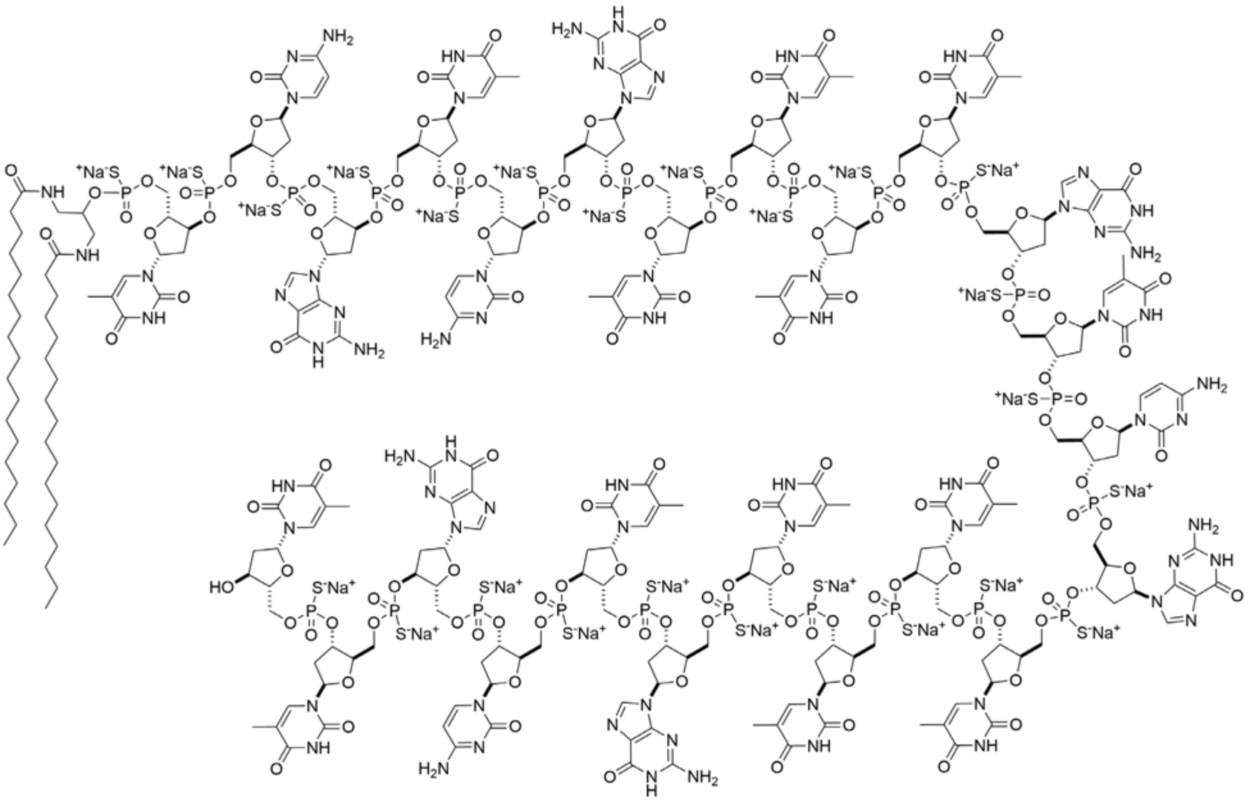

Structurally, each amphiphile (Amph) component is a fusion comprised of 3 domains:

- Two 18-carbon acyl lipids that mediates association with albumin to facilitate trafficking to the lymph nodes and permits cell membrane insertion;
- A flexible linker that improves pharmaceutical properties in Amph-Peptides; the flexible linker is omitted in Amph-CpG-7909;
- A bioactive peptide or oligonucleotide.

Results of in vivo preclinical pharmacology studies showed that the greatest T cell responses to mKRAS peptides were achieved when the animals were administered Amph-Peptide(s) in combination with Amph-CpG adjuvant. The 7 peptide antigens included in ELI-002 have been previously evaluated in humans (without amphiphile modification) and were observed to be safe and well tolerated after subcutaneous (SC) administration in studies sponsored by Targovax ASA [Ericksen et al., 2017; Palmer et al., 2020].

ELI-002 targets mKRAS-driven malignancies expressing the most common oncogenic mutant forms of KRAS. Upon SC injection, the components of the vaccine are drained from the peripheral tissues into the lymph nodes. Here mKRAS Amph-Peptides are taken up by antigen-presenting immune cells for processing through the proteasomal pathway. Moynihan et al reported that the immunogenicity of peptide vaccines could be increased by maximizing delivery to lymph nodes, where T-cell responses generated using the amphiphile approach were increased compared to soluble controls [Moynihan et al, 2018] (see Section 4.6.2).

This protocol, ELI-002-001, describes a First-in-Human trial to evaluate safety and tolerability of ELI-002 as adjuvant therapy for subjects with KRAS/NRAS mutated solid tumors. Subjects will be treated and evaluated for safety and efficacy of ELI-002 comprised of 1.4 mg Amph modified KRAS peptides (0.7 mg per Amph-Peptide) admixed with escalating doses of the Amph modified CpG oligonucleotide adjuvant Amph-CpG-7909. The ELI-002 admixture is prepared by combining the separately provided products in a diluent prior to dosing. During the Phase 1 (dose escalation) study, ELI-002 will be comprised of 1.4 mg of 2 Amph modified KRAS peptides G12D and G12R admixed with Amph-CpG-7909 to provide ELI-002 2P. Elicio plans to investigate the use of an Amph-Peptide 7P drug product containing all 7 Amph-Peptides (G12D, G12R, G12V, G12A, G12C, G12S, G13D) admixed with Amph-CpG-7909 (ELI-002 7P) in future clinical trials.

The trial will be monitored in accordance with the Sponsor’s procedures, which comply with the ethical principles of Good Clinical Practice (GCP) as required by the major regulatory authorities, and in accordance with the Declaration of Helsinki.

4.1. Summary of Pancreatic Cancer and Other Solid Tumors

4.1.1. Pancreatic Cancer

With an estimated number of 60,430 new cases in the United States (US) in 2021, PDAC is among the most common malignancies. Moreover, it is one of the most lethal cancers, as indicated by mortality estimated at 48,220 (~80%) [Siegel et al, 2020]. PDAC is the third leading cause of cancer death [Siegel et al, 2021], with 133,300 estimated deaths for 2018 in the US and Europe [Malvezzi et al, 2018].

Surgery (pancreatectomy) is the only curative option in PDAC. Even with surgery, prognosis remains poor due to the high propensity of the tumor for loco-regional and hepatic recurrence. In the National Cancer Database population, the 5-year survival in subjects undergoing pancreatectomy was only 23.3% [Sener et al, 1999]. Therefore, surgery alone is clearly an inadequate approach to achieve long-term disease control in subjects with resectable PDAC. Historical use of adjuvant therapy with gemcitabine, or FOLFIRINOX combination chemotherapy improved the disease-free survival (DFS) and overall survival (OS) outcomes for subjects with resectable disease, but the majority of these subjects relapsed and died from recurrent disease [Conroy et al, 2018; Oettle et al, 2007]. Contemporary neoadjuvant therapy with FOLFIRINOX-based neoadjuvant therapy is a standard approach for locally advanced pancreatic cancer since it has been shown to improve the surgical resection rates and clinical outcomes in locally advanced PDAC [Chawla and Ferrone, 2019], and promising phase 2 data with neoadjuvant gemcitabine/nab-paclitaxel [Philip et al, 2020] indicate that an alternative regimen has activity. Fluoropyrimidine-based chemoradiation may be used as well [NCCN PDAC guidelines Version 1.2022]. Despite this, there is a need for improved adjuvant therapies for subjects who have undergone pancreatic resection surgery as survival rates for pancreatic cancer have mostly remained unchanged since the 1970s [Ansari et al, 2016; Macdonald and Mair, 2016].

PDAC is a disease that is almost entirely related to *KRAS* mutations. The *KRAS* gene encodes the KRAS protein, which is a small (21 kDA) guanosine triphosphatase (GTPase) that functions as a simple binary ON–OFF molecular switch, cycling between active GTP-bound and inactive guanosine diphosphate (GDP)-bound states [Vigil et al, 2010]. Cancer-associated *RAS* genes contain missense mutations that encode single amino acid substitutions primarily (98%) at 1 of 3 mutational hot spots: glycine-12 (G12), glycine-13 (G13), or glutamine-61 (Q61) [Waters and Der, 2018]. These mutations render KRAS persistently GTP-bound and constitutively active, resulting in overstimulation of effector signaling pathways to drive cancer growth.

The majority of KRAS mutations occur at codon 12. A conversion of the amino acid glycine (G) to aspartic acid (D) at this site (KRAS G12D) is the most frequent KRAS mutant in human gastrointestinal cancers and has been identified in approximately 33% to 52% of pancreatic cancers [Biankin et al 2012; Hendifar et al 2020; Guo et al 2020] and 34% to 37% of CRCs [Gong et al 2017; Prior et al, 2012; Price et al, 2020]. KRAS G12D plays a vital role in the control of tumor metabolism [Vigil et al, 2010].

In addition to G12D, there are 5 other clinically relevant but less frequently identified mutants with amino acid substitutions at the same site (G12V, G12R, G12A, G12S, and G12C) and 1 peptide with a mutation at codon 13 (G13D). After G12D, G12V mutation is the next most frequent mutation in PDAC, identified in about 36% of these tumors, followed by G12R (about 12% of mutations) [Biankin et al, 2012]. The remaining codon 12 mutations (G12A, G12C, and G12S) each account for about 3% to 4% of PDAC-associated codon 12 KRAS mutations [Prior et al, 2012]. G13D is the most frequent codon 13 mutation but is only identified in <1% of PDAC tumors.

Administration of a combination of these peptides provides coverage for multiple potential mutations that might be present.

Circulating tumor deoxyribonucleic acid DNA (ctDNA) is a promising biomarker in the management of many cancers, including breast, colorectal [Bettegowda et al, 2014; Reinert et al, 2019; Tie et al, 2016; Tie et al, 2019], non-small cell lung cancer (NSCLC) [Abbosh et al, 2017], bile duct [Jensen et al, 2020], and pancreatic cancers [Diaz and Bardelli, 2014; Lee, Lipton, et al, 2019].

In the metastatic setting, PDAC subjects with positive MRD, as measured by mKRAS ctDNA exhibit shorter OS [Perets et al, 2018]. Presence of MRD pre-surgery predicted for poor outcomes. Similarly, measurement of mKRAS ctDNA identifies PDAC subjects who have undergone pancreatectomy but are at high risk of recurrence. For example, persistence of ctDNA immediately following the postoperative period was associated with a high rate of recurrence and shorter median recurrence-free survival (5 months) than those without detectable postoperative ctDNA (15 months) [Groot et al, 2019]. Similar data were obtained by analysis of a prospective cohort in Australasia who underwent surgery and adjuvant gemcitabine-based chemotherapy; ctDNA 4-8 weeks post-surgery was assessed [Lee, Lipton, et al., 2019]. In this study, ctDNA detection post-surgery had 100% positive predictive value at a median follow-up of 38.4 months; ctDNA predicted relapse free (hazard ratio [HR]: 5.4; 95% confidence interval [CI]: 1.9-15.2; $p<0.0001$). Likewise, high pre-operative ctDNA levels associated with shorter overall survival (HR: 2.8, 95% CI: 1.8-4.6, $p = 0.001$) and were an independent prognostic factor compared to pathologic features of pancreatic cancer in multivariate analysis of an operable pancreatic cancer patient cohort [Singh et al., 2015]. Subjects who remain ctDNA positive despite chemotherapy and successful resection are therefore a high-risk population.

Likewise, multiple studies have shown the prognostic value of postsurgical serum carbohydrate antigen 19-9 (CA19-9), with failure to normalize correlating with early recurrence. Most patients are assessable for CA19-9, with the exception of 5-10% of the population who lack Lewis blood antigen expression and cannot synthesize the sialylated carbohydrate. For example, in a retrospective analysis of 525 patients, postsurgical elevations in CA19-9 predicted subsequent radiographic relapse [Rieser et al., 2018]. In a Japanese cohort of 119 patients who completed adjuvant chemotherapy, median relapse-free survival (RFS) was significantly shorter in patients with CA19-9 above the upper limit of normal (ULN) versus those with lower levels (10.4 vs 29.6 months, $p<0.001$), median overall survival was also shorter (24.7 vs 92.1 months; $p<0.001$), and CA19-9 was an independent prognostic factor (HR: 2.72) associated with short RFS [Imaoka et al., 2016]. Prospective studies have also found that postoperative CA19-9 predicts overall survival: among 385 RTOG9704 patients, those with a CA19-9 ≥90 U/mL had a 3-year survival of 2%, whereas those with CA19-9 <90 U/mL had a 3-year survival of 32% [Berger et al., 2008]. In Protocol ELI-002-201, subjects in the Phase 1 cohort will be enrolled based on high risk ctDNA or serum tumor biomarkers.

Activating KRAS mutations turn on canonical cancer-related pathways but also contribute to extensive metabolic reprogramming of pancreatic cancer cells. KRAS is mutated in >90% of PDACs and is a well-validated driver of not only initiation but also PDAC growth and maintenance [Eser et al, 2014]. Recently, FDA approval of small molecules targeting $KRAS^{G12C}$ such as Sotorasib [Hong et al., 2020] suggest it may be possible to target cysteine containing KRAS alleles, however G12D and G12V are predominate in pancreatic cancer, and non G12C alleles are also common in colorectal and NSCLC, making it clear that additional immunotherapy strategies are urgently needed.

In a small trial from Norway, Palmer et al utilized a vaccine containing the same mKRAS peptides present in ELI-002. Prolonged survival was observed in one quarter (8 of 32) of the pancreatic cancer subjects that underwent resection surgery and were immunized; this trial also provided evidence that humans can mount T-cell responses against KRAS peptides and established a dosing schedule that was the basis for the design of the ELI-002-001 trial [Palmer et al, 2020]. Thus, KRAS directed vaccines represent a promising strategy to target this lethal cancer.

ELI-002 is well suited to pancreatic cancer since it covers common mutations in KRAS that are present in the majority of pancreatic adenocarcinoma cases. A large comprehensive trial of 3,594 pancreatic tumor samples submitted for analysis at Foundation Medicine (Cambridge, MA) has recently been published [Singhi et al, 2019]. In this analysis 3,150 of the 3,594 (88%) tumors harbored an mKRAS allele. Similar high prevalence of mKRAS was evident in the international consortium trial of US, Australian, and Canadian pancreatic tumors where 93% of 142 tumors had exon 2 mutations (Table 4); 46% were G12D, 36% were G12V, and 12% were G12R [Biankin et al, 2012]. The proportion and specific mKRAS alleles identified are consistent across studies, as confirmed by the Cancer Genome Atlas Network, where 93% (140 of 150) tumors harbored mKRAS including 44% that were G12D, 29% that were G12V, and 20% that were G12R [Cancer Genome Atlas Network et al, 2017]. Likewise, the Johns Hopkin's cohort identified KRAS mutations in 96% (49 of 51 pancreatic tumors), with 42% G12D, 27% G12V, 21% G12R, 6% G13D and 2% G12C [Sausen et al., 2015] and a German trial identified KRAS mutations in 79% (141 of 179 pancreatic tumors); 49% were G12D, 34% G12V, 14% G12R and 2% G12C [Haas et al, 2017].

Therefore, the majority of pancreatic cancer subjects are anticipated to have a KRAS mutation appropriate for ELI-002-001 trial participation. KRAS is a validated oncogene that when mutated is required for PDAC growth and maintenance so that target loss is not anticipated. Enhancing the T-cell immune response to PDAC by utilizing the targeted KRAS ELI-002 product candidate has the potential to improve the outcomes of subjects who have undergone pancreatic resection surgery.

Table 4: KRAS Mutation Spectrum in Pancreatic Cancer

	**G12D** (%)	**G12V** (%)	**G12R** (%)	**G13D** (%)	**G12C** (%)	**G12A** (%)	**G12S** (%)	**Reference:**
Pancreatic	46	36	12	0	0	0	0	Biankin et al, 2012
Pancreatic	42	27	21	6	2	0	2	Sausen et al, 2015
Pancreatic	49	34	14	nr	2	nr	nr	Haas et al, 2017
Pancreatic	44	29	20	nr	nr	nr	nr	Cancer Genome Atlas Network, 2017
Pancreatic	33	23	11	nr	1	nr	nr	Hendifar et al, 2020
Pancreatic	52	28	12	4	4	0	0	Guo et al, 2020

4.1.2. Colorectal Cancer

4.1.2.1. Introduction

Colorectal cancer (CRC) is the fourth most frequently diagnosed cancer and the second leading cause of cancer death in the US. In 2018, 97,220 new cases of colon cancer and approximately 43,030 cases of rectal cancer have been diagnosed. During the same year, an estimated 50,630 people died of colon and rectal cancers combined [Miller et al; 2019; Siegel et al, 2020].

Non metastatic colon cancer is generally treated with curative intent by colectomy and, in many cases, adjuvant chemotherapy. The prognosis for patients with CRC depends on the stage at diagnosis: the 5-year survival rate is up to 90% for stage I, but only <15% for advanced stages. Over half of the cases are diagnosed at a higher stage of disease (III and IV). Treatment usually involves the complete primary tumor resection and appropriate chemotherapy.

4.1.2.2. Adjuvant Therapy in the Case of Localized CRC Disease

Adjuvant therapy confers a survival advantage in patients with stage III or high-risk stage II resected colon cancer. High risk stage II colon cancer are defined as stage T4 tumor, poor differentiation, cancer embolus, lymph node harvested number less than 12 and obstruction or perforation, baseline carcinoembryonic antigen (CEA) >5 ng/L, large vessel invasion, perineural invasion, and extramural vascular invasion [Amri et al, 2016].

For patients with high-risk stage II or stage III colon cancer, adjuvant chemotherapy with fluoropyrimidine and oxaliplatin reduces the risk of recurrence and death by approximately 30%. Oxaliplatin is associated with peripheral neurotoxicity. Consideration of disease stage and pathologic features, microsatellite instability (MSI) status, toxicity profiles, and patient age, comorbidities, aid in decision-making regarding the use of adjuvant therapy.

The National Comprehensive Cancer Network (NCCN) guidelines are based on the IDEA study, published in 2018, an academic collaboration of clinicians and statisticians who were involved in 6 randomized, Phase 3 clinical trials enrolling in 12,834 patients with stage III colon cancer in 12 countries. The 3-year rates of DFS were 74.6% in the 3-month adjuvant therapy group as compared with 75.5% in the 6-month therapy group. The 3-year rates of DFS were 64.4% in high-risk patients: T4 (61.8%), or N2 (61.4%) [Grothey et al, 2018].

Tumor (T) and nodal (N) stage were grouped together to make a pragmatic choice between low-risk (T1–T3,N1) and high-risk (T4 and/or N2). A difference of approximately 20% at 3 years was shown between these cancers.

According to the NCCN Guidelines for patients with low-risk stage III disease (T1–3,N1), the preferred regimen is CAPEOX for 3 months or FOLFOX for 3 to 6 months. For patients with high-risk stage III disease (T4,N1–N2 or any T and N2), the preferred regimen is CAPEOX for 3 to 6 months or FOLFOX for 6 months. Other options include capecitabine (6 months) and 5-fluorouracil (5-FU) for 6 months [Grothey et al, 2018].

After adjuvant cytotoxic therapy, no further treatment is recommended. Circulating tumor DNA is positive in 28.6% defining a subgroup of high-risk Stage II (T4N0) and a 39.4% subgroup of high-risk Stage III (T4, N1 or T Any, N2) patients [Kasi et al, 2020].

4.1.2.3. Rectal Cancer

Patients with rectal cancer were specifically excluded from these studies because of potential toxicity and the confounding impact of radiotherapy.

Although conclusive data on the benefits of adjuvant therapy in patients with stage II/III rectal cancer are lacking, the NCCN panel recommends its use. Choice of regimen depends on initial clinical staging and predicted circumferential resection margin status during excision surgery (such as total mesorectal excision). Pathologic evaluation of the resection margin on the excised rectum has been considered important for determining the risk of local recurrence. A margin of ≤1 mm is considered by some to be a negative prognostic factor for local recurrence [Benson et al, 2018].

FOLFOX or CAPEOX are preferred adjuvant regimens, with 5-FU/leucovorin or capecitabine used in some circumstances, such as when strong neoadjuvant responses to 5-FU or capecitabine were previously obtained during therapy. Neoadjuvant or adjuvant 5-FU or capecitabine-based chemoradiation, or total neoadjuvant therapy are all standard options for rectal cancer [NCCN rectal cancer guidelines V1.2022].

4.1.2.4. Colorectal Liver Metastases

Aside from localized CRC, up to 25% of patients with CRC present with simultaneous metastases and the liver is frequently the only metastatic site.

Approximately 35% to 55% of patients will develop hepatic metastases during the course of their disease. In some cases, colorectal liver metastases are resectable, ie, an R0 resection can be performed, leaving at least a 20% to 25% of total liver volume as future liver remnant. Surgical resection represents the only chance of long-term survival [Akgul et al, 2014].

Preoperative factors found to be independent predictors of poor survival are a primary tumor at stage T4, ≥4 liver metastases, the largest liver metastasis ≥5 cm in diameter, and a serum CEA level ≥5 ng/mL. According to these factors, resectable patients could be divided in high-risk patients (3 or more factors) and low-risk patients (less than 3 factors) [Adam, et al, 2012].

The optimal sequencing of systemic therapy and resection in colorectal liver metastases remain unclear. Most studies report on perioperative (neoadjuvant plus postoperative) systemic therapy [Krell et al, 2019], with some series reporting on patients with resectable disease who undergo resection first, followed by postoperative adjuvant chemotherapy.

A 2012 meta-analysis identified 3 randomized clinical trials comparing surgery alone to surgery plus systemic therapy. The analysis showed a benefit of chemotherapy in progression-free survival and DFS but not in OS. Another meta-analysis combined data on 1,896 patients and also found that perioperative chemotherapy improved DFS but not OS [Ciliberto et al, 2012].

In high-risk patients, perioperative combination chemotherapy should be administered. Those who had received neoadjuvant chemotherapy had a better median OS (38.9 months versus 28.4 months) than those had not received it, and 5 years OS rate of 39% versus 33% (p=0.028). In low-risk patients, no significant difference in median survival (60.0 m versus 60.0 m) was observed.

Colorectal metastatic disease sometimes occurs in the lung. Complete resection based on the anatomic location and extent of disease with maintenance of adequate function is required. Most of the treatment recommendations for metastatic colorectal liver disease also apply to the treatment of colorectal pulmonary metastases.

Colon, rectal, and oligometastatic CRC patients who show minimal residual DNA on ctDNA analysis with no radiographic evidence of disease following prior surgery and neoadjuvant/adjuvant chemotherapy, will be eligible to participate in this protocol.

4.1.2.5. Frequency of KRAS and NRAS Mutations in CRC

KRAS genotyping is done for every CRC patient because of its impact on the treatment and patients are classified as KRAS wild type (WT) or KRAS mutated.

Since 2008, anti-epidermal growth factor receptor (EGFR) therapy is restricted to KRAS WT patients as it has been shown that KRAS mutated tumors do not respond [Boeckx et al, 2015; Modest et al, 2016].

KRAS mutations occur in approximately 40% of the cases, especially in exon 2, codons 12 (70%-80%) and 13 (15%-20%) (Table 5) [Gong et al, 2017; Zhang et al, 2015; Prior et al, 2012; Price et al, 2020]. In exon 2, mutations are common in codons 12: G12D, G12V, G12C and G12A. Much less frequent KRAS mutations are mainly located in exon 3, codons 59–61, and in exon 4, codons 117 and 146 [Afrasanie et al, 2019].

Since the amino acid sequence surrounding positions 12 and 13 are identical in KRAS and NRAS, T cells generated to these mKRAS epitopes cross-react and are effective in targeting mNRAS at the same amino acids [Chatani and Yang, 2020]. NRAS mutations also occur in CRC in approximately 4% of cases, also in exon 2, codons 12 and 13 [Douillard et al, 2013; Summers et al, 2017; Zhang et al, 2015]. Therefore, ELI-002 can additionally target NRAS mutations at positions 12 and 13.

Table 5: Colon and Rectal Cancer KRAS and NRAS Mutation Spectrum

	G12D (%)	G12V (%)	G12R (%)	G13D (%)	G12C (%)	G12A (%)	G12S (%)	Reference
**KRAS**								
Colon	34	12	0	7	0	1.4	5.1	Gong et al, 2017
Colon	41	20	0.7	20	6	3.6	8	Zhang et al, 2015
Colon	35	20	0.9	19	10	6	7	Prior et al, 2012
Colon	37	23.6	Nr	13.3	9.6	10.7	4.4	Price et al, 2020
Rectal	33	23	0.7	17	8	7	6	Prior et al, 2012
**NRAS**								
Colon	45	14	nr	nr	14	4	nr	Douillard et al, 2013
Colon	25	13	nr	nr	62.5	nr	nr	Summers et al, 2017
Colon	52	4	nr	9	13	nr	nr	Zhang et al, 2015

nr=not reported

The full analysis of the RAS gene exons 2, 3, and 4 revealed that these mutations correlate with certain typical clinical, pathological and molecular features. The mutations of exon 2 and codon 12 are associated with the well/ moderately differentiated adenocarcinoma subtype and the mucinous subtype [Dobre et al, 2015].

Limited data is available evaluating the possible prognostic role of mKRAS at the initiation of the treatment. One study showed that patients with KRAS mutation who presented with stage IV had a higher mortality rate (34% versus 18.5%) and reduced OS (23.5 months versus 14 months) compared to patients without this mutation. Also, the presence of the KRAS mutation was found to be an independent risk factor for reduced survival [Heinemann et al, 2009].

4.1.2.6. Importance of ctDNA and CEA Assessment and Follow-up in CRC

Different authors reported that the detection of ctDNA in CRC patients with stage I, II, or III after treatment was associated with a shorter survival and disease recurrence. Patients with higher levels of mutant ctDNA and higher mutation loads for the detected mutations in *KRAS* or *BRAF* genes evinced shorter OS. Diehl et al observed that CRC patients with detectable ctDNA after surgery generally relapsed within 1 year and considered ctDNA as a highly specific biomarker of tumor dynamics. This might indicate that ctDNA levels in plasma after surgery represent a reliable marker of residual disease [Diehl et al, 2008]. Subsequent studies have now confirmed the utility of serial ctDNA sampling for relapse prediction. Reinert et al observed a 2 to 15 (mean 10) months lead time on detection of metastatic recurrence compared to conventional follow-up [Reinert et al, 2016].

These all support the further development of ctDNA as a biomarker for the detection of early diseases and MRD following curative resection, relapse assessment, treatment response, and the development of chemoresistance [Vymetalkova et al, 2018]. Fan et al systematically reviewed comprehensive data from published studies and showed that a ctDNA-positive status after treatment was associated with a worse prognosis [Fan et al, 2017].

In addition, ctDNA assessment using a personalized ctDNA method for 535 colon cancer patients in a real-world sample have confirmed the association with relapse and allow precise definition of the risk subgroups. High risk stage 2 (T4N0 with MRD rate 28.6%), high risk stage 3 (T4N1-2; TanyN2 with MRD rate 39.4%) and surgically resected oligometastatic patients (MRD rate 49%) have high rates of ctDNA detection indicating their unmet need for effective therapy [Kasi et al 2020]. In a prospective, multicohort study [Tarazona et al, 2020] with 84 patients where MRD+ status after adjuvant chemotherapy was determined using a personalized ctDNA assay, all MRD+ patients relapsed and the association was strongly statistically correlated (HR: 27.9; 95% CI: 9.2-85.1 months; p<0.0001). Another study in a cohort of 230 patients with Stage II colon cancer further confirmed that a plasma-based ctDNA analysis predicts recurrence [Tie et al, 2016]. Patients who were ctDNA positive postoperatively were at extremely high risk of radiologic recurrence (HR 18) and ctDNA positivity immediately after completion of chemotherapy was associated with poorer RFS (HR 11). Postsurgical ctDNA analysis using a plasma-based ctDNA assay in 100 patients with Stage III colon cancer demonstrated that MRD+ status was associated with inferior RFS [Tie et al, 2019].

Likewise, the association between elevations in serum CEA with relapse following surgery and adjuvant chemotherapy in colorectal cancer was examined in a large patient series. The

CEAwatch study, consisting of 3223 patients with Stage I-III R0 resected CRC in the Netherlands, evaluated recurrence outcomes in patients followed with usual care versus patients with intensified follow-up, including CEA monitoring every 8 weeks, with more frequent monitoring if the CEA value was >2.5 ng/mL or if there was a 20% increase compared with the previous value. The results showed that with the intensified follow-up protocol a significantly higher proportion of radiographic relapse was detected compared with usual care (odds ratio: 1.8; 95% CI: 1.33-2.50; p=0.0004), indicating earlier detection [Verberne et al., 2015; Verberne et al., 2017]. A Memorial Sloan Kettering review of 728 R0 resected patients showed that individual CEA measurements above a threshold of 15 ng/mL had a low (2%) false positive rate, and no elevations above 35 ng/mL were false positive [Litvak et al., 2014].

ELI-002 will be evaluated in such MRD^+ patients and those with successive rises in CEA/elevated CEA above 15 ng/mL.

4.1.2.7. Colorectal Cancer and Vaccines

Approaches under investigation to further improving therapy are vaccinations with peptides alone, peptide-expressing viruses, peptide-loaded APCs or application of peptide-specific T cells.

In the process of cancer development mutations resulting in neoantigens can emerge in every coding region of the DNA. Neoantigens caused by KRAS are one example for point mutations in CRC. Early approaches could prove that peptides derived from mKRAS can stimulate cytotoxic T-lymphocytes in vitro [Gedde-Dahl, 1994] as well as in CRC [Shono et al, 2003]. A subsequent study also investigated the cytotoxic activity of mKRAS peptide-induced cytotoxic T lymphocytes [Khleif et al, 1999]. Only 2 of 10 CRC patients showed induction of peptide-specific $CD8^+$ but in addition, these cytotoxic T cells were able to lyse HLA-A2-positive target cells incubated with the 10-mer mutant peptide. Similar results were obtained in a xenograft study, where peptide-specific T cells were able to delay the growth of KRAS-mutant pancreatic tumors [Wang, et al, 2016].

In vaccination trials with peptides derived from mKRAS, there were early indications of potential clinical benefits for patients. Two of 7 CRC patients had an immune response to a mKRAS peptide vaccine, and vaccination correlated to DFS [Toubaji et al, 2008]; the treatment was well tolerated with no serious systemic effects and Grade 1 to 2 reactogenicity. One of 4 GI tumor patients, an individual with CRC, exhibited induction of G12D-specific T cells in response to mRNA vaccine administration [Cafri, et al., 2020]. In a case report, 1 CRC patient was treated with activated T cells recognizing G12D KRAS [Tran, et al, 2016]. After a single infusion, all 7 lung metastases regressed until 1 metastatic lesion progressed at 9 months. This patient underwent surgical resection for the single relapsed site and has now been in ongoing remission for >4 years [Chatani and Yang, 2020].

To further enhance the immunological response in CRC patients, Rahma et al combined the peptide vaccination of mKRAS with IL-2 or granulocyte-macrophage colony-stimulating factor (GM-CSF) [Rahma, 2014]. The strongest immune response could be detected in the group with GM-CSF as adjuvant; all CRC patients had an increase in interferon gamma (IFNγ) producing, specifically-activated T cells. Despite the high immune response rate, none of these advanced metastatic patients showed clinical response and disease progressed in all cases.

4.1.3. Non-small Cell Lung Cancer

NSCLC is the leading cause of cancer mortality in the United States, with an estimated incidence in 2021 of over 235,000 and estimated deaths of over 131,000 [Siegel et al., 2021]. While surgically treated patients with Stage 1A enjoy long-term disease-free survival, relapse is common following surgery for Stage 1B, II, or IIIA disease, with 30%, 60%, and 75% mortality [Ravdin 2006]. However, neither adjuvant chemotherapy [Strauss et al., 2008] nor radiation therapy [PORT Meta-analysis Trialists Group, 2005] improves overall survival for IB tumors. For Stage II tumors, meta-analysis of multiple trials [Pignon et al., 2008] supports a modest adjuvant chemotherapy benefit (HR 0.89, 95% CI, 0.82–0.96; p= 0.005); similar benefit was observed in neoadjuvant meta-analysis [Burdett et al., 2007], and radiation appears to shorten rather than benefit survival outcome [PORT Meta-analysis Trialists Group, 2005]. For Stage IIIA tumors, both neoadjuvant [Burdett et al., 2007] and adjuvant [Pignon et al., 2008] chemotherapy confer modest benefit. Immunotherapy with checkpoint inhibition using durvalumab was associated with longer progression-free survival in NSCLC patients following chemoradiation, with a median 16.8 versus 5.6 months (HR 0.52, 95% CI 0.42-0.65, p <0.001) [Antonia et al., 2017]. Similarly, in a Phase 3 trial of 1005 NSCLC patients following complete surgical resection and adjuvant platinum-based chemotherapy whose tumors expressed PD-L1 on 1% or more of tumor cells, patients randomized to atezolizumab had not reached the median disease-free survival compared with best supportive care where the median was 35.3 months (HR: 0.66; 95% CI 0.50-0.88; p=0.0039) [Felip, et al., 2021]. In addition, nivolumab was approved by the FDA for neoadjuvant therapy of NSCLC based on the randomized Checkmate-816 trial which compared 3 cycles of combined nivolumab/platinum-based chemotherapy versus platinum based chemotherapy alone; the median event-free survival was 31.6 months versus 20.8 months (HR: 0.63; p=0.0052) [FDA Press Release]. Since immunotherapy appears to be more active in NSCLC, novel agents like ELI-002 with an immune mechanism of action hold promise to improve outcomes in NSCLC.

Therefore, ELI-002 will be studied in patients with KRAS-mutated NSCLC who have completed neoadjuvant or adjuvant chemotherapy, chemoradiation, or checkpoint inhibitor therapy, as appropriate for their stage of tumor, when they remain ctDNA positive indicating a high risk for relapse.

4.1.3.1. KRAS Mutations in NSCLC

Tumor genetics is assessed as standard of care to detect actionable mutations in ALK, ROS1, EGFR, NTRK and BRAF, and KRAS is frequently included in the DNA sequence methods. KRAS mutations (Table 6) are present in approximately 26% of NSCLC patients [Dogan et al., 2012], with a high proportion of G12C alleles (Table 6); NRAS mutations are very rare [Illei et al., 2017].

Table 6: KRAS Mutation Spectrum in Non-small Cell Lung Cancer

	G12D (%)	G12V (%)	G12R (%)	G13D (%)	G12C (%)	G12A (%)	G12S (%)	Reference
NSCLC	17	21	nr	nr	39	11	nr	Dogan et al., 2012
NSCLC	17	21	2	2	41	8	4	Prior et al., 2012

4.1.3.2. Circulating Tumor DNA in NSCLC

The TRACERx (Tracking Non-Small-Cell Lung Cancer Evolution Through Therapy [Rx]) study demonstrated the ability of a personalized ctDNA assay to detect MRD post-operatively in patients with non-small cell lung cancer [Abbosh et al., 2017]. Additionally, ctDNA kinetics reflected the tumor response to treatment, and predicted a patient's early cancer recurrence before relapse could be detected via standard imaging studies. Consistent results were observed in a smaller single-center study of 37 NSCLC patients, where detection of ctDNA within 4 months of completion of locoregional therapy for Stage 1B-III tumors was associated with poor freedom-from-progression and disease-specific survival [Chaudhuri, et al., 2017]. Reduction from baseline of <50% in ctDNA level was significantly associated with disease progression (HR: 2.28; p= 0.013) in a cohort of 99 metastatic NSCLC patients undergoing PD-L1 checkpoint inhibitor therapy [Nabet, et al., 2020]. Therefore, additional effective therapies are needed for ctDNA positive NSCLC patients who have a high risk of relapse following surgery and adjuvant/neoadjuvant therapy; post-baseline reductions in ctDNA holds promise as a potential early endpoint.

4.1.4. Ovarian Carcinoma

Ovarian cancer, associated with primary peritoneal cancer and cancer of the Fallopian tube, is the leading cause of gynecologic cancer death in developed countries and most often presents at an advanced stage. At diagnosis, 80% of patients present with stage 3 and 10% with stage 4. The 5-year survival rate is approximately 30%.

During 2021, the American Cancer Society estimates that approximately 21,410 women will be diagnosed with ovarian carcinoma, and approximately 13,770 patients will die as a result of the disease [Siegel et al, 2021]. Worldwide, 295,414 new patients per year (2018), and 184,799 deaths [Bray et al, 2018]. Ovarian cancer is the second most common cancer of the female genital tract. In the US, it is responsible for more cancer deaths than all other gynecologic tumors.

The prevalence is approximately 10 times its incidence, with Surveillance, Epidemiology, and End Results (SEER) data estimating 233,364 women in the US living with the disease in 2017 [SEER, 2020]. A paucity of symptoms indicative of early disease, coupled with the absence of validated screening tools, makes ovarian carcinoma the deadliest of the gynecologic malignancies, with 10-year DFS rate less than 10%.

Malignant epithelial tumors are the most common type of ovarian cancer and comprise almost 90% of cases, with the remainder comprised of low-grade borderline tumors. The treatment of ovarian cancer has been based mainly on tumor grade and stage, but the histologic subtype is very important in-patient management.

Most patients have advanced disease at initial diagnosis: primarily stage III (75-80%), and fewer stage IV (5-10%). Regardless of tumor type, the estimated 5-year OS according to the 26th International Federation of Gynecology and Obstetrics annual report is 50% to 65% for stage III and 20% to 25% for stage IV.

The current standard-of-care for the treatment of this majority of patients involves cytoreductive surgery and platinum-based chemotherapy. The NCCN Ovarian Cancer guidelines (Version 1.2022) include as preferred chemotherapy regimens for ovarian cancer. These are paclitaxel

175/carboplatin, as well as 5-FU/leucovorin/oxaliplatin with or without bevacizumab, or capecitabine/oxaliplatin with or without bevacizumab.

Although approximately 80% of patients achieve complete clinical response through extensive cytoreductive surgery and platinum- and taxane-based chemotherapy, most ultimately develop recurrent chemo-resistant disease.

Therapeutic options for patients in complete response following combined modality therapy depend on the primary chemotherapy regimen used, and the *BRCA1/2* mutation status. Poly (ADP-ribose) polymerase inhibitors are effective in the *BRCA1/2* mutated subpopulation of tumors [Armstrong et al, 2020; Boussios et al, 2019].

- Patients who are in clinical remission, defined as no evidence of disease, after primary chemotherapy without bevacizumab have several options:
  1. Investigational therapy such as ELI-002 in the context of a clinical trial
  2. Observation
  3. Olaparib or niraparib for those with a *BRCA1* or *BRCA2* mutation

Therefore, ELI-002 will be evaluated in the population whose tumors lack mutation in BRCA1/2 who are in clinical remission after primary chemotherapy.

4.1.4.1. Ovarian KRAS Mutations

Tumor molecular testing is frequently performed in ovarian cancer to assess for BRCA1/2 mutation, and KRAS is often included. KRAS mutations (Table 8) are present in primary tumors at 9.6% to 12.5% of serous, 11.76% to 33% of endometrioid, 43.6% to 61% of mucinous, and 14% to 26.3% of clear cell histologies [Auner et al, 2009; Morikawa et al, 2018; Perren, 2016; Rechsteiner et al, 2013; Dobrzycka et al, 2011]. Borderline tumors may harbor KRAS mutations but have a favorable prognosis [Prat et al, 2018; Rambau et al, 2017]. The spectrum of mutations in ovarian cancer is similar to gastrointestinal tumors with the most frequent mutation being the G12D allele (Table 7). NRAS mutations are rare in ovarian tumors.

Table 7: KRAS Mutation Spectrum in Ovarian Tumors

	G12D (%)	G12V (%)	G12R (%)	G13D (%)	G12C (%)	G12A (%)	G12S (%)	Reference:
Ovarian	40	23	3	7	13	7	0	Auner et al, 2009

Table 8: KRAS Mutation Frequency in Ovarian Tumors

Subtype of Ovarian Carcinoma	KRAS Mutation Frequency (%)
High-grade serous	9.6-12.5
Low-grade serous	29.5
Mucinous	43.6-61
Clear Cell	14-26.3
Endometrioid	11.76-33
Mucinous Borderline	33-78

4.1.4.2. Circulating Tumor DNA in Ovarian Cancer

Promising data using ctDNA methods have been observed that demonstrate that MRD+ status following chemotherapy predicts short time to disease progression [Kim et al, 2019; Giannopoulou et al, 2018]. In addition, biochemical relapse of ovarian cancer (as determined by CA-125 serum concentrations >35 U/mL) predated radiographic progression by approximately 4.5 months in a cohort of 98 Dutch patients [van der Burg et al., 1990]. Therefore, ELI-002 will be evaluated in ovarian cancer patients with KRAS mutations who are ctDNA positive following therapy and those who are at high relapse risk due to serum CA-125 tumor biomarker above the normal range.

4.1.5. Cholangiocarcinoma

Cholangiocarcinomas (CCAs) are rare and aggressive tumors. About 8,000 patients are diagnosed with CCA each year in the US. In 2016, there were 7,000 new patients in the US and 5,600 deaths [Yao et al, 2016]. An increasing incidence has been reported worldwide since the last 10 years, especially the intrahepatic subtype: the number of intrahepatic cholangiocarcinoma (iCCA) has more than doubled and the global mortality rate has increased to 36% [Kirstein et al, 2016].

CCAs are one of the most fatal cancers: although 1-year mortality has improved over time, the 5-year survival is still as low as 10%. The only curative option for patients with CCA is surgical resection, but many patients are diagnosed with distant disease, and 10% to 45% of those considered appropriate for surgery are found to be unresectable at laparotomy [Blechacz et al, 2017]. At advanced stage, CCA has a devastating prognosis with a median OS of only 12 to 15 months.

CCAs are diverse biliary epithelial tumors involving the intrahepatic, perihilar, and distal biliary tree. They are categorized according to anatomical location as iCCA, perihilar cholangiocarcinoma (pCCA), or distal cholangiocarcinoma (dCCA). The 5-year survival rates are: intrahepatic 21% to 40%, perihilar 10% to 37%, distal 23% to 45% [Blechacz et al, 2017].

For patients with advanced-stage or unresectable CCA, first-line systemic therapy with cisplatin and gemcitabine provides a survival advantage over gemcitabine alone [Valle et al, 2010]; however, there is limited effectiveness: median OS with the standard regimen is less than 1 year [Rizvi et al, 2018], and there is no standard for later-line therapy.

In the adjuvant setting, there was no adjuvant benefit observed in R0 resection, however with R1 resection a modest benefit to a 6-month course of oral capecitabine chemotherapy was observed in the BILCAP trial for patients with iCCA and dCCA [Primrose et al, 2019; Shroff et al, 2019]. The NCCN Hepatobiliary Cancer guidelines (Version 5.2021) recommend adjuvant capecitabine, alternative fluoropyrimidine-based, gemcitabine-based, or chemoradiation for R1 resected tumors, but leave the choice of therapy or observation to the treating physician's discretion for R0 resected tumors.

4.1.5.1. Cholangiocarcinoma KRAS Mutations

The frequency of KRAS mutations has been reported higher at 36% to 40% of extrahepatic CCA compared with 8% to 24% in iCCA in US-based series [Ettrich et al, 2019; Churi et al, 2014]. Likewise, in an Italian series, the KRAS mutation frequency was higher at 47.4% of extrahepatic CCA versus 15.7% in iCCA [Simbolo et al, 2014]. A limited number of manuscripts have reported the spectrum of KRAS mutations (Table 9), where G12D is the most common observed allele in iCCA but G12C is more common in extrahepatic CCA [Zou et al, 2014; Churi et al, 2014].

Table 9: KRAS Mutation Spectrum in Cholangiocarcinoma

	G12D %	G12V %	G12R %	G13D %	G12C %	G12A %	G12S %	Reference:
Cholangiocarcinoma (iCCA)	41	35	nr	nr	12	12	nr	Zou et al, 2014
Cholangiocarcinoma (iCCA)	50	17	8	nr	nr	17	8	Churi et al, 2014
Cholangiocarcinoma (extrahepatic)	13	13	nr	25	38	13	nr	Churi et al, 2014
Cholangiocarcinoma (not specified)	nr	20	60	20	nr	nr	nr	Ettrich et al, 2019

iCCA=intrahepatic cholangiocarcinomas; nr=not reported

Circulating tumor DNA (ctDNA) has been investigated for potential use in detecting actionable mutations such mKRAS [Ettrich et al, 2019]; however, studies of relapse prediction in an adjuvant setting have not yet been reported. Small studies of serum tumor biomarkers have been performed in this rare tumor type. For example, Kato and colleagues [Kato et al., 2016] studied 143 patients with extrahepatic CCA, finding that those whose initial postoperative CA19-9 was >37 U/mL had increased recurrences within 1 year (69.4% vs 42.3%, p=0.012) and shorter survival (24.0 vs 42.9 months, p<0.001). Similarly, elevated postoperative CA19-9 >37 U/mL predicted short survival for iCCA patients [Kondo et al., 2014].

4.1.6. Gallbladder Cancer

Cancers of the gallbladder are nearly all adenocarcinomas, which arise from the secretory cells. The gallbladder is the most common primary cancer site among the biliary tracts.

According to GLOBOCAN 2018 data, gallbladder cancer is the 22nd most incident but 17th most deadly cancer worldwide [Bray et al, 2018]. About 3,700 people are diagnosed with gallbladder cancer and 2,000 people die from the disease in the US each year. In 2018, about 219,000 people worldwide were estimated to have been diagnosed with gallbladder cancer. This constitutes 1.2% of all cancer diagnoses.

Gallbladder cancer is disproportionately deadly because it is rarely found before it has advanced or metastasized. In fact, in the US, only about 1 in 5 gallbladder cancers are diagnosed in the early stages, and median survival for advanced stage cancer is no more than about a year [Rawla et al, 2019].

The optimal treatment strategy for patients with resected cancer has not been determined. A phase 3 trial confirmed that gallbladder carcinoma patients treated with adjuvant mitomycin C and 5-FU had a higher 5-year survival rate of 26% compared with 14% in the control group (p=0.0367) [Takada et al, 2002]. For R0 or R1 resected gallbladder cancer patients, the NCCN Hepatobiliary Cancer guidelines [Version 5.2020] recommend capecitabine as the preferred adjuvant regimen, with a list of acceptable neoadjuvant regimens (BIL-C). Some institutions use fluoropyrimidine based chemoradiation for patients with R1 positive margins or regional nodes.

In a small study of patients with gallbladder cancer, KRAS mutations were detected in 8 primary tumors cases, with a limited spectrum 75% G12R and 25% G12C [Kim et al, 2000]. Circulating tumor DNA assessment has not been widely studied in gallbladder cancer. ELI-002 will be evaluated in patients with successfully surgically resected gallbladder cancer who have mKRAS.

4.2. Brief Summary of Nonclinical Pharmacology

A subset of nonclinical studies evaluated ELI-002a, an investigational product consisting of 8 components: 7 lipid-conjugated KRAS peptide-based antigens (Amph-Peptides, Table 3) and a lipid-conjugated immunostimulatory oligonucleotide adjuvant (Amph-CpG-7909, Figure 1). Nonclinical studies with ELI-002a or components of ELI-002a have been performed to support the clinical investigation of the multivalent product.

ELI-002 2P is an investigational therapeutic vaccine consisting of a subset of 2 of the 7 Amph-Peptides (G12D and G12R) and Amph-CpG-7909. Elicio plans to investigate the use of an Amph-Peptide 7P drug product containing all 7 Amph-Peptides (G12D, G12R, G12V, G12A, G12C, G12S, G13D) admixed with Amph-CpG-7909 (ELI-002 7P) in future clinical trials. Initial clinical data with ELI-002 2P, as well as the nonclinical data described in the Investigator's Brochure, are expected to support continued development of the multivalent product. ELI-002 is proposed as post-surgical adjuvant treatments of MRD in patients with KRAS viral oncogene homolog mutated solid tumors.

The objective of the nonclinical pharmacology program for ELI-002 was to demonstrate, in a C57BL/6 mouse model, the improved potency of the amphiphile-conjugated subunits (both antigens and adjuvant) in eliciting cellular immune responses to KRAS peptides. The C57BL/6 model was selected because there is no readily available immunocompetent animal model of

cancer related to KRAS mutations and because the primary objective of these studies was to justify the components of the vaccine by demonstrating differential immunogenic responses between amphiphile-conjugated and nonconjugated vaccines. The CpG-1826 adjuvant was used in place of the clinical ELI-002 CpG-7909 adjuvant in most mouse pharmacology studies because it is more potent to agonize mouse Toll-like-receptor 9 (TLR-9). The nonclinical pharmacology program for ELI-002 is outlined in Table 10.

Table 10: Nonclinical Pharmacology studies of mKRAS Vaccine in Support of ELI-002 (Model = Female C57BL/6 Mice, 5, 10 or 15/group)

Study Title	Groups and Test Article<br>Test Article Manufacturing Site/Batch #	Doses/Dose Volume	ROA/Treatment Regimen
KRAS Imgen #4	7 groups:<br>sKRAS-WT + CPG<br>sKRAS G12D + Amph-CpG;<br>sKRAS G13D + Amph-CpG;<br>Amph-KRAS G12D + Amph-CpG;<br>Amph-KRAS G13D + Amph-CpG;<br>KRAS G12D + pIC;<br>No Treatment (PBS)	KRAS peptide (Amphiphilic or soluble): 20 µg[1]; CpG-1826 (Amphiphilic or soluble) at 5 nmol and pIC at 50 µg.<br>Dose volume: 100 µL administered divided as a SC injection on either side of the tail head (50 µL per injection site)	ROA: SC<br>Prime: Day 0<br>Boost: Day 14
KRAS Imgen #7	12 groups:<br>sKRAS-WT + CPG<br>sKRAS G12D + CpG-1826;<br>sKRAS G12A + CpG-1826;<br>sKRAS G12R + CpG-1826;<br>sKRAS G12V + CpG-1826<br>sKRAS G12R + pIC;<br>sKRAS G12V + pIC;<br>Amph-KRAS G12D + Amph-CpG-1826;<br>Amph-KRAS G12A + Amph-CpG-1826;<br>Amph-KRAS G12R + Amph-CpG-1826;<br>Amph-KRAS G12V + Amph-CpG-1826;<br>No Treatment (PBS)	KRAS peptide (Amphiphilic or soluble): 20 µg[1]; CpG-1826 (Amphiphilic or soluble) at 5 nmol and pIC at 50 µg.<br>Dose volume: 100 µL administered divided as a SC injection on either side of the tail head (50 µL per injection site)	ROA: SC<br>Prime: Day 0<br>Boost: Day 14

Study Title	Groups and Test Article<br>Test Article Manufacturing Site/Batch #	Doses/Dose Volume	ROA/Treatment Regimen
KRAS Imgen #8	6 groups:<br>sKRAS WT + CpG-1826<br>sKRAS G12D + CpG-1826<br>sKRAS G12R/V + CpG-1826<br>Amph-KRAS G12D + Amph-CpG-1826<br>Amph-KRAS G12R/V + Amph-CpG-1826<br>No Treatment (PBS)	KRAS peptide (Amphiphilic or soluble): 20 µg[1]; CpG-1826 (Amphiphilic or soluble) at 5 nmol.<br>Dose volume: 100 µL administered divided as a SC injection on either side of the tail head (50 µL per injection site)	ROA: SC<br>Prime: Day 0<br>Boost: Day 14, Day 23
KRAS Imgen #9	7 groups:<br>sKRAS WT + CpG-1826 (bw)<br>sKRAS WT + CpG-1826 (w)<br>sKRAS G12D + CpG-1826 (bw);<br>sKRAS G12D + CpG-1826 (w);<br>Amph-KRAS G12D + Amph-CpG-1826 (bw);<br>Amph-KRAS G12D + Amph-CpG-1826 (w);<br>No Treatment (PBS)	KRAS peptide (Amphiphilic or soluble): 50 µg[1]; CpG-1826 (Amphiphilic or soluble) at 5 nmol.<br>Dose volume: 100 µL administered divided as a SC injection on either side of the tail head (50 µL per injection site)	ROA: SC<br>Prime: Day 0 (w and bw)<br>Boost: Day 7 (w)<br>Boost: Day 14 (w and bw)
KRAS Imgen #12	7 groups:<br>sKRAS G12D + sol CpG-1826<br>sKRAS G12D + Amph-CpG-1826<br>sKRAS G12D + pIC<br>Amph-KRAS G12D + Amph-CpG-1826<br>sKRAS WT + sCpG-1826<br>Amph-CpG-1826 (No antigen)<br>No Treatment (PBS)	KRAS peptide (Amphiphilic): 18.75 µg[1] (50 µg based on total construct mass); KRAS peptide (soluble): 50 µg[1]; CpG-1826 (Amphiphilic or soluble) at 5 nmol; pIC 50 µg.<br>Dose volume: 100 µL administered divided as a SC injection on either side of the tail head (50 µL per injection site)	ROA: SC<br>Prime: Day 0<br>Boost: Day 14, Day 27, Day 41, Day 55, and Day 66

Study Title	Groups and Test Article<br>Test Article Manufacturing Site/Batch #	Doses/Dose Volume	ROA/Treatment Regimen
KRAS Imgen #14	7 groups:<br>sKRAS G12D + sol CpG-1826<br>sKRAS G12D + Amph-CpG-1826<br>sKRAS G12D + pIC<br>Amph-KRAS G12D + Amph-CpG-1826<br>sKRAS WT + sol CpG-1826<br>Amph-CpG-1826<br>PBS (No treatment control group)	KRAS peptide (Amphiphilic): 7.5 µg[1] (20 µg based on total construction mass); KRAS peptide (soluble): 20 µg[1]; CpG-1826 (Amphiphilic or soluble) at 5 nmol and pIC at 50 µg.<br>Dose volume: 100 µL administered divided as a SC injection on either side of the tail head (50 µL per injection site)	ROA: SC<br>Prime: Day 0<br>Boost: Day 14, Day 28, Day 42
KRAS Imgen #14.2	5 groups:<br>Vehicle (5% EtOH, 3.3 mM HCl)<br>Adjuvant (Amph-CpG-7909) alone<br>Amph-KRAS mutant pool (7 peptides) + 0.6 nmol Amph-CpG-7909<br>Amph-KRAS mutant pool (7 peptides) + 3 nmol Amph-CpG-7909<br>Amph-KRAS mutant pool (7 peptides) + 15 nmol Amph-CpG-7909	KRAS peptide pool: 30 µg/dose of Amph-KRAS G12D; 3 µg/dose of all others: Amph-KRAS G12A, Amph-KRAS G12C, Amph-KRAS G12S, Amph-KRAS G12R, Amph-KRAS G12V, Amph-KRAS G13D for a total of 48µg of Amph-Peptide per dose<br>Amph-CpG-7909 alone at 15 nmol (125 µg/100 µL)<br>Amph-CpG-7909 in combination with Amph-KRAS peptide pool at 0.6, 3 and 15 nmol (5, 25 and 125 µg/100 µL)<br>Dose volume: 100 µL administered divided as a SC injection on either side of the tail head (50 µL per injection site)	ROA: SC<br>Prime: Day 0<br>Boost: Days 10, 17, 24, and 39
KRAS Imgen #15	9 groups:<br>Untreated<br>Amph-KRAS G12D + 0.1nmol Amph-CpG-1826<br>Amph-KRAS G12D + 1nmol Amph-CpG-1826<br>Amph-KRAS G12D + 5nmol Amph-CpG-1826<br>Amph-KRAS G12D + 5nmol sCpG-1826<br>sKRAS G12D + 0.1nmol Amph-CpG-1826<br>sKRAS G12D + 1nmol Amph-CpG-1826<br>sKRAS G12D + 5nmol Amph-CpG-1826<br>sKRAS G12D + 5nmol sCpG-1826	KRAS peptides (Amphiphilic): 7.5 µg[1] (20 µg based on total construct mass); KRAS peptides (soluble): 20 µg[1]; Amph-CpG-1826 was dosed at 0.1, 1 and 5 nmol and soluble CpG-1826 was dosed at 5 nmol<br>Dose volume: 100 µL administered divided as a SC injection on either side of the tail head (50 µL per injection site)	ROA: SC<br>Prime: Day 0<br>Boost: Day 14, Day 28, Day 74

Study Title	Groups and Test Article<br>Test Article Manufacturing Site/Batch #	Doses/Dose Volume	ROA/Treatment Regimen
KRAS Imgen #26	PBS (vehicle)<br>Amph-KRAS G12D (fresh preparation) + 5 nmol Amph-CpG-1826<br>Amph-KRAS G12D (aged preparation) + 5 nmol Amph-CpG-1826	Amphiphilic KRAS peptides (fresh or aged) were dosed at 30 µg based on total construct mass; Amph-CpG-1826 was dosed at 5 nmol<br>Dose volume: 100 µL administered divided as a SC injection on either side of the tail head (50 µL per injection site)	ROA: SC<br>Prime: Day 0<br>Boost: Day 13, Day 27
BLAST P Report	In silico BLAST search for Amph-Peptide sequences	N/A	N/A

KRAS= Kirsten rat sarcoma; mKRAS = mutant Kirsten rat sarcoma; PBS = phosphate-buffered saline, pIC = polyinosinic:polycytidylic acid; ROA = route of administration; SC = subcutaneous; w = weekly; bw = biweekly; Amph-G#X = Amph-KRAS G#X, eg, Amph-G12D = Amph-KRAS G12D
[1]Based on peptide mass alone

These nonclinical pharmacology studies demonstrate that despite the historically poor immunologic responses of mice to mKRAS peptides, they consistently mounted potent immune responses to the amphiphile conjugated vaccine that were superior to control soluble vaccines lacking the amphiphile modification.

- The weight of evidence of in vivo data indicates that immunization with amph-mKRAS peptides and amph-CpG1826 or soluble mKRAS and Amph-CpG-1826 stimulated a stronger memory immune response, as indicated by cytokine release, to the corresponding short and long peptides than did immunization with soluble mKRAS and soluble CpG-1826 or soluble mKRAS + pIC. (KRAS Imgen #7; KRAS Imgen #12, KRAS Imgen #8, KRAS Imgen #9).
- The immunologic response was characterized by a strong Th1 response and cytolytic function against KRAS-specific targets, both in vitro and in vivo. Very low amounts of Th2 cytokines like IL4 and IL10 were generated in vitro (KRAS Imgen #12, KRAS Imgen #14).
- The most robust responses were generated when both peptide and adjuvant were amph-conjugated (KRAS Imgen #4).
- Restimulation with short peptides elicited a weaker response than restimulation with long peptides (KRAS Imgen #14, KRAS Imgen #7, KRAS Imgen #8).
- Restimulation with long wide type (WT) peptide did elicit responses in cells from animals vaccinated with mutant peptide but responses were lower than those induced by the corresponding mutant peptide (Imgen #4, Imgen #14). During toxicity evaluation WT Ras tissues were unaffected, suggesting that low level responses to WT peptide may not present a safety concern.
- Increasing the frequency of dosing did not affect the immune response towards long peptide stimulation. However, it did seem to improve responses to short-peptide stimulation (KRAS Imgen #9).
- Amphiphilic CpG-7909 (Amph-CpG-7909) dose-dependently increased the immune response to KRAS antigen. No significant immune response was detected at the lowest concentration of CpG; however, medium and high concentrations generated robust immune responses (KRAS Imgen #14.2).
- The minimum adjuvant (aCpG) dose needed to achieve a robust immune response was 5 nmol (KRAS Imgen #15, KRAS Imgen #14.2).
- The minimum effective dose (MED) identified for immunogenicity of ELI-002 in mice, based on available data, was 48 µg total peptide (30 µg 12D and 3 µg/peptide of the other 6 peptides; 5 nmol or 25 µg aCpG7909) (KRAS Imgen #14.2).
- There was no effect of the vehicle formulation change on immunogenicity (KRAS Imgen #14.2).
- There was no effect of the formation of the Amph-TM-G12D thiomorpholinone isomer on immunogenicity of Amph-G12D (KRAS Imgen #26).

- Despite the lower dosage of Amph-KRAS peptide used when based on peptide mass alone, the T-cell responses in mice treated with the Amph-KRAS peptide vaccines surpassed the responses of mice treated with soluble equivalents (KRAS Imgen #12, KRAS Imgen #14, KRAS Imgen #15).
- BLAST searches using either the wild type or mutant sequence demonstrated identity with sequences in over 300 human proteins, and particularly those that are other products of human RAS genes [BLAST Results Summary], however there has been no evidence of autoimmune phenomena described in other KRAS vaccine trials using the same peptide sequences. There was no non-RAS potential cross-reactivity identified by BLAST-P alignment.

Since antitumor activity of mKRAS specific T cells can lead to durable clinical response [Tran et al, 2016, Chatani and Yang, 2020], the ELI-002 in vivo pharmacology observations demonstrating increased antigenicity and potent CD4 and CD8 T cell responses suggest potential for antitumor activity and clinical benefit.

4.3. Brief Summary of Pharmacokinetics and Toxicokinetics

Pharmacokinetics/toxicokinetics (PK/TK) are not assessed for vaccinations since systemic exposure is not observed. Therefore, PK/TK will not be assessed in the nonclinical development program for ELI-002.

4.4. Brief Summary of Toxicology

ELI-002a is an investigational version of ELI-002 used in nonclinical toxicology testing. ELI-002a consists of all 7 Amph modified KRAS peptides (Amph-Peptides, Table 3) and the lipid-conjugated immunostimulatory oligonucleotide adjuvant (Amph-CpG-7909, Figure 1). Nonclinical studies with ELI-002a or components of ELI-002a have been performed to support the clinical investigation of the multivalent product.

ELI-002 2P is an investigational therapeutic vaccine consisting of a subset of 2 of the 7 Amph-Peptides (G12D and G12R) and Amph-CpG-7909. Elicio plans to investigate the use of an Amph-Peptide 7P drug product containing all 7 Amph-Peptides (G12D, G12R, G12V, G12A, G12C, G12S, G13D) admixed with Amph-CpG-7909 (ELI-002 7P) in future clinical trials. Initial clinical data with ELI-002 2P, as well as the nonclinical data described in the Investigator's Brochure, are expected to support continued development of a multivalent product. ELI-002 is proposed as post-surgical adjuvant treatment of MRD in patients with KRAS/NRAS viral oncogene homolog mutated solid tumors including CRC and PDAC.

4.4.1. Repeat-Dose Toxicity Studies

4.4.1.1. Study No. 2795-001: Amph-CpG-7909 (with and without Amph-Peptide-G12D): A 4-Week Subcutaneous Injection Tolerability Study in Male C57BL/6 Mice

Subcutaneous administration of the adjuvant (Amph-CpG-7909) with and without peptide (Amph-G12D) to male C57BL/6 mice once weekly for 4 weeks was well tolerated. Moderate to severe erythema noted at 200/80 µg adjuvant/peptide (0.2 mL total dose volume) was deemed to be adverse.

Based on the available data, Amph-CpG-7909 alone was tolerable up to 100 µg (0.2 or 0.4 mL total dose volume) and Amph-CpG-7909 + Amph-Peptide was tolerable at 100/80 µg adjuvant/peptide (0.2 or 0.4 mL total dose volume) as well as 200/80 µg adjuvant/peptide at a higher dose volume (0.4 mL total dose volume). Based on these data, the high dose of adjuvant was set at 125 µg in a total injection volume of 300 µL.

4.4.1.2. Study 2795-002: A GLP 3-Month Study of ELI-002 by Subcutaneous Injection in C57BL/6 Mice with a 5-Week Recovery Period

Two control groups (a phosphate-buffered saline control and an adjuvant only control) were included. Groups of mice (15-20/sex/group) were dosed on Days 1, 8, 15, 22, 29, 36 and 64 and euthanized on Days 8, 43, 71, and 99. Three dose levels were employed, with the same dose of Amph-Peptide (48 µg of the aggregate of 7 peptides) at each dose level; only the adjuvant dose varied.

The administration of 48 µg Amph-Peptides with either 5 µg or 25 µg Adjuvant was well tolerated with a dose-dependent reduced (relative to the high dose) incidence and severity of local irritation and limited associated effects. However, high dose adjuvant (125 µg Adjuvant alone and 48 µg Amph-Peptides/125 µg Adjuvant) was not well tolerated, with 3 of 90 animals in each group (with and without peptide) animals euthanized in extremis as a result of increased severity of clinical observations around the injection site. There were also positive dermal scores; clinical pathology and organ weight including increased lymph node size, effects indicative of an immune/inflammatory response; and histopathology consisting of injection site ulceration, inflammation and osseous proliferation, as well as the spread of extensive abdominal/peritoneal and regional inflammation. These effects were all considered subsequent to exacerbated local toxicity at the injection site and were considered adverse. Therefore, the no-observed-adverse-effect level (NOAEL) was 48 µg Amph-Peptides/25 µg Adjuvant.

The mouse toxicology data indicate that the adjuvant, rather than the peptide, was primarily responsible for the observed toxicity in these studies. During toxicity evaluation WT RAS tissues were unaffected, suggesting that low level responses to WT peptide may not present a safety concern. The dose multiples from the highest dose of adjuvant that was well tolerated in mice (25 µg) to the proposed adjuvant doses in humans of 0.1, 0.5, and 2.5 mg are 599x, 120x, and 24x, respectively (based on body surface area). As a large molecule expected to undergo intracellular rather than hepatic or renal clearance, a fixed dose of ELI-002 will be administered in this study.

4.5. Safety Pharmacology

Safety pharmacology studies were not conducted since soluble mKRAS peptides identical in amino acid sequence to those in ELI-002 have been well tolerated when previously evaluated in clinical studies in cancer subjects [Gjertsen et al, 2001; Wedén et al, 2011; Abou-Alfa et al, 2011; Rahma et al, 2014; Toubaji et al, 2008; Palmer et al, 2020] and Amph-mKRAS peptides are not expected to have any effects on core physiological systems that are different from those of soluble mKRAS peptides.

Similarly, soluble CpG-7909 has been previously evaluated and found to be well tolerated in clinical studies in normal volunteers and cancer subjects [Cooper et al, 2004; Krieg et al, 2004]

with an MTD observed in one oncology study of myalgia and constitutional effects [Zent et al, 2012] and Amph-CpG-7909 is not expected to have any effects on core physiologic systems that are different from those of soluble CpG-7909.

4.6. ELI-002: An Investigational Treatment for KRAS/NRAS Mutated Solid Tumors

4.6.1. Mechanism of Action

Therapeutic vaccines for cancer commonly seek to stimulate the expansion of cytotoxic T cells specific to antigens from malignant cells. Following activation, these antigen-specific cells infiltrate tumor sites, recognize tumor cells expressing antigen through major histocompatibility complex (MHC) presentation, and execute cytotoxic effector functions to kill target cells. ELI-002 employs this general paradigm to target mKRAS and mNRAS-driven malignancies expressing mutant forms of KRAS/NRAS. The sequence of amino acids is identical between KRAS and NRAS surrounding positions 12 and 13, and the National Cancer Institute has demonstrated that RAS-specific T cells cross recognize both mKRAS and NRAS tumors [Chatani and Yang, 2020]. Upon SC injection, the components of the vaccine are drained from the peripheral tissues into the lymph nodes. KRAS/NRAS Amph-Peptides are then taken up by antigen-presenting immune cells for processing through the proteasomal pathway. Peptide fragments produced through proteasomal degradation are loaded onto MHC molecules and presented on the cell surface for recognition by T lymphocytes through T-cell receptor interactions. In parallel, Amph-CpG-7909 is taken up by lymph node resident immune cells for endocytic processing. Immune cells expressing TLR-9 are activated by CpG, a process which leads to expression and secretion of pro-inflammatory factors supportive of adaptive immunity including T lymphocyte activation. Within this pro-inflammatory context, T-cells capable of specific recognition of antigen engage with APCs and are clonally expanded, activated, and licensed for killing of antigen-bearing target cells [Yang et al, 2016a; Yang et al, 2016b; Yang et al, 2017].

4.6.2. Amphiphile Lymph Node Delivery Background

Subunit vaccines are an attractive strategy for eliciting therapeutic immunity given the ease, speed, and low cost of manufacturing for small molecules and biopolymers such as peptides and oligonucleotides [Liu et al, 2014]. However, subunit vaccines consisting of linear peptides and molecular adjuvants often elicit only minimal immunological responses in animals and humans. Previous studies have demonstrated that poor lymphatic drainage, and thus inefficient delivery to immune cells, is a significant factor leading to the observed lack of potency for these vaccination strategies.

Empirical studies have established 2 competing mechanisms for solute drainage from peripheral tissues: (1) passage through the endothelial basement membranes into systemic blood circulation and (2) drainage from the tissue into lymphatic vessels and circulation through the lymph system. The predominant pathway taken for any individual molecule following SC injection is determined by molecular size. Specifically, solutes <3 nm in size easily pass through the endothelial basement membranes and are rapidly cleared from the tissues into the blood before elimination in the kidneys and liver. Importantly, molecules of this size are not delivered to

draining lymph nodes. Conversely, for solutes >3 nm in size, passage into blood vessels is inefficient, and molecules >5 nm in size are excluded completely via physiological barriers from clearance into the blood. Rather molecules of this size are drained through convective transport through the tissues into lymphatic vessels and perfuse the lymph nodes prior to re-entry into the blood via the thoracic duct through the subclavian vein. Thus, the minimal potency observed for subunit vaccines can be attributed, in substantial part, to their lack of delivery into the lymph nodes based on their size of <3 nm. Consequently, improved delivery of these subunit vaccine components to lymph nodes holds promise for greatly enhancing overall immunological responses and therapeutic efficacy.

Correlative to the established size-dependent biodistribution paradigm, clinical studies have demonstrated that compounds <3 nm in size which bind avidly to albumin after injection are effectively concentrated in draining lymph nodes rather than being cleared through the systemic circulation. Further, these albumin-binding compounds efficiently accumulate in antigen-presenting immune cells once delivered into the draining lymph nodes. The basis for this paradoxical observation is the innate biodistribution pattern of endogenous albumin. Albumin, a 66.5 kDa protein, is present at high concentrations throughout the body, and is exclusively drained from the peripheral tissues into the lymphatics due to its high molecular weight (MW). Therefore, compounds which otherwise would be cleared into the blood due to their small molecular size take on the biodistribution properties of albumin by binding to the larger molecule.

Vaccine responses have been boosted by the use of altered peptide ligands that enhance T-cell responses against tumor antigens. Moynihan et al found that vaccines that were modified with albumin-binding domains helped to transport these molecules throughout the lymphatic system and increased T-cell response [Moynihan et al, 2018]. Liu et al reported this lymph-node targeting as “albumin hitchhiking.” In mice, structurally optimized CpG-DNA/peptide-amph-vaccines increased lymph node accumulation and resulted in a 30-fold increase in T-cell priming and enhanced anti-tumor efficacy with reduced systemic toxicity [Liu et al, 2014].

Building on these observations, the amphiphile approach seeks to improve vaccine potency by targeting conventional vaccine components for specific delivery to the lymph nodes through simple chemical modifications which impart affinity for albumin. Specifically, peptide antigens or molecular adjuvants are chemically conjugated to fatty-acid moieties which take advantage of the natural function of albumin as a fatty-acid transporter. Antigens and adjuvants modified in this way reversibly and persistently associate with albumin at the injection site and are carried by albumin into lymph nodes where they efficiently engage immune cells to promote the expansion of antigen-specific immunity.

4.6.3. Known and Potential Risks and Benefits

Elicio will evaluate safety and tolerability of ELI-002 as adjuvant therapy for subjects with KRAS/NRAS mutated PDAC and other solid tumors who have MRD to determine whether the potential for clinical benefit is sufficient to warrant further testing while minimizing risk. ELI-002 is an investigational therapy and this is the first trial to evaluate ELI-002 in humans, therefore, not all aspects pertaining to potential risks and benefits from it are completely known at this time. However, there is an extensive body of preclinical studies as well as human studies

conducted by other institutions on soluble versions of the peptide and CpG components used in ELI-002 which are reassuring.

In addition, there are considerable data available on the risks and benefits from cancer vaccines as a class of therapy. The technologies for cancer vaccination have been extensively reviewed by Kudrin [Kudrin, 2012]. In brief, therapeutic cancer vaccines can be available off-the-shelf (recombinant antigen cocktails, recombinant microorganisms, whole tumor cell derived (allogeneic), oncolytic viruses, anti-idiotypic antibodies, DNA and gene-therapy based products) which could be manufactured and distributed worldwide and personalized cancer vaccines (autologous cells and antigens, adoptive cell transfer) which are heavily dependent on specialized centers of expertise and manufacturing. With extensive prior efforts and trials, one cancer vaccine: Dendreon's PROVENGE® (sipuleucel-T), a dendritic cell for metastatic castration-resistant prostate cancer, was approved by the Food and Drug Administration (FDA) in 2010 which has established the clinical application of cancer vaccines.

These cancer vaccines were generally well tolerated and most infusion-related side effects lasted 1 or 2 days. These are the potential risks which have been associated with PROVENGE cancer vaccine: acute infusion reaction, chills, fever, and fatigue back pain, joint ache, fatigue, nausea, headache and fever [PROVENGE® USPI].

As reported by Wedén et al, no signs of toxicity were reported and no serious adverse events (SAEs) attributable to the vaccine treatment were observed in any of the subjects following soluble mKRAS peptide vaccination, including long-term survivors who received up to 30 injections each [Wedén et al, 2011].

Likewise, 5 subjects with advanced pancreatic cancer were treated with synthetic RAS peptides used as a cancer vaccine. All subjects were vaccinated at Day 0, and booster vaccinations were performed similarly on Days 7-10 and at 1 month. Further booster vaccinations were performed at 4-6–week intervals or as long as the subject's immunological response and overall performance status permitted over a period of 2 to10 months. The treatment was well tolerated and could be repeated multiple times in the same subject. No sign of toxicity and no adverse events (AEs) were observed [Gjertsen et al, 1996].

ELI-002 is expected to elicit common side effects often associated with vaccines such as local skin reaction, fever, fatigue, pain, among others which generally subside within a day or two. Reactogenicity refers to a subset of reactions that occur soon after vaccination and are a physical manifestation of the inflammatory response to vaccination. In clinical trials, information on expected signs and symptoms after vaccination is actively sought (or 'solicited'). These symptoms may include pain, redness, swelling or induration for injected vaccines, and systemic symptoms, such as fever, myalgia, headache, or rash. The broader term "safety" profile refers to all AEs that could potentially be caused/triggered or worsened at any time after vaccination, and includes AEs, such as anaphylactic reactions, diseases diagnosed after vaccination and autoimmune events.

The administration of 48 µg Peptide with either 5 or 25 µg Adjuvant was well tolerated in the rodent Study No. 2795-002 with a dose-dependent reduced incidence and severity of local irritation and limited associated effects. Therefore, the NOAEL was 48 µg Amph-Peptides/25 µg Adjuvant. Local reactions were considered adverse at the high adjuvant Amph-CpG-7909 dose in the GLP toxicity study which confirms the importance of reactogenicity monitoring. Local

reactions and some flu-like symptoms are expected in subjects who receive peptide injections, and there is always a possibility of hypersensitivity reactions. Injection site reaction is the only AE known to be attributed to ELI-002. Reactogenicity monitoring will occur throughout the ELI-002-001 trial (see Section 12.2.1.3)

Since ELI-002 has increased potency compared to prior soluble vaccines in T-cell production and because dose-dependent increases in cytokines were observed, there will be a 7-day enrollment stagger between treatment of the first subject in a given cohort and the treatment of subsequent subjects and laboratory cytokine monitoring will be included for all subjects.

It is expected that ELI-002 based upon its demonstrated mechanism of action, will likely evoke immunological response leading to clinical benefit among protocol subjects.

4.6.4. Trial Rationale

KRAS is mutated in one quarter of human solid tumors, including the vast majority of pancreatic cancers, and substantial portions of colorectal, non-small cell lung, ovarian, gallbladder and bile duct cancers. KRAS is a validated oncogene that when mutated drives tumor growth and maintenance. Enhancing the T-cell immune response to tumors by utilizing the targeted KRAS ELI-002 product candidate has the potential to improve the outcomes of subjects who have undergone resection surgery and/or who have MRD following locoregional therapy, a clinical setting with the highest ratio of ELI-002 induced T cells to target tumor cells. For example, the pancreatic population selected for this trial is comprised of subjects who have already received chemotherapy/ chemoradiation (in the neoadjuvant and/or adjuvant setting) as well as pancreatectomy with an R0 (no residual tumor cells observed) or R1 (microscopic residual tumor cells observed) outcome, yet who have poor prognosis with persistent ctDNA post-surgery indicating an expected median relapse-free survival (RFS) of only 5 months [Groot et al, 2019]. The selection of cancer patients with MRD for evaluation of a cancer immunotherapy product candidate has several potential advantages:

- Maximization of the effector T-cell:target tumor cell ratio by minimization of tumor burden by successful prior therapy
- Maximization of T-cell trafficking potential because the fibrous stromal present in advanced tumors that can exclude T-cells has not yet formed
- Clinical settings where cytotoxic chemotherapy is not utilized, preserving the full potential of induced T-cells to persist and traffic

The Phase 1 study is an open-label, single-arm, dose-escalation trial incorporating a 3+3 design in subjects with KRAS/NRAS mutated PDAC and other solid tumors who have MRD. Approximately18 subjects are planned to be enrolled in Phase 1 to evaluate the safety, tolerability, and exploratory pharmacodynamic efficacy of SC ELI-002 using the adjuvant at 3 planned dose levels (Amph-CpG-7909 0.1 mg SC, 0.5 mg SC, 2.5 mg SC) in combination with a fixed dose of Amph-Peptides (700 µg each). Additional cohorts may be added to explore intermediate or higher dose levels. This will be based on the cumulative safety review and preliminary review of efficacy. If additional cohorts are added, this will be documented in a study memo sent to all clinical sites and submitted to IRBs, per IRB guidance.

During the study, ELI-002 will be comprised of 1.4 mg of 2 Amph modified KRAS peptides, Amph-G12D, and Amph-G12R (0.7 mg/peptide) admixed with Amph-CpG-7909 to provide ELI-002 2P. Elicio plans to investigate the use of an Amph-Peptide 7P drug product containing all 7 Amph-Peptides (G12D, G12R, G12V, G12A, G12C, G12S, G13D) admixed with Amph-CpG-7909 (ELI-002 7P) in future clinical trials.

Since ELI-002 has increased potency compared to prior soluble vaccines in T-cell production and because dose-dependent increases in cytokines were observed, there will be a 1-week stagger between enrollment of the first subject in a cohort and the subsequent subjects and laboratory cytokine monitoring will be included for all subjects. In addition, all patients will be asked to stay within 1 hour of the clinic following the first dose of ELI-002. In the absence of toxicity during the dose-limiting toxicity (DLT) assessment window, ELI-002 will be escalated. Planned cohort dose levels are listed below:

- Cohort 1 Dose Level 1: Amph-CpG-7909 0.1 mg with Amph-Peptides
- Cohort 2 Dose Level 2: Amph-CpG-7909 0.5 mg with Amph-Peptides
- Cohort 3 Dose Level 3: Amph-CpG-7909 2.5 mg with Amph-Peptides

Cohorts will consist of 3 to 6 subjects (the number will depend on whether DLTs are observed). In order to ensure an adequate number of evaluable subjects, up to 6 subjects may be enrolled per cohort even in the absence of a DLT.

Dose escalation from one cohort to the next will be determined by the Safety Monitoring Committee (SMC), and will be based on treatment-emergent AEs, clinical laboratory data, physical examination (PE) findings including vital signs, after all subjects within a cohort have completed 28 days.

In any cohort, if none of the subjects experience a DLT during the first 28 days following first administration of ELI-002, the dose escalation will occur, and 3 subjects will be enrolled in the cohort at the next dose level. However, if 1 of the 3 or 4 initial subjects in a cohort experiences a DLT, then 3 (or 2, if 4 were originally enrolled) additional subjects will be enrolled at the same dose level. If 2 or more of 3 subjects within a cohort experience a DLT, then this dose will be considered the toxic dose. If only 1 of 6 subjects within a cohort experiences a DLT during the first 28 days of treatment, then the next cohort may begin enrollment. If 2 or more of 6 subjects within a cohort experience a DLT during the first 28 days, then this dose will be considered the toxic dose. The trial may proceed at a lower dose, at a less frequent schedule, or omitting the Amph-CpG-7909 adjuvant while continuing to administer the peptides, based on emerging toxicity or pharmacodynamic data until the maximum tolerated dose (MTD) is determined.

A trial memo will be sent to all sites following each SMC meeting summarizing the decisions; if additional cohorts are to be enrolled, the dose level and schedule will be provided.

The recommended Phase 2 dose (RP2D) will be defined in consideration of the MTD (if any), safety data, and pharmacodynamic data. The RP2D will be communicated to all sites in a trial memo following the last SMC cohort review. Once the RP2D is determined, the cohort evaluating the Phase 2 dose will be expanded as needed so that there is safety experience with at least 6 subjects at this dose level.

Subject replacement: If a subject discontinues for any reason other than toxicity prior to completing 28 days of ELI-002 therapy, a replacement subject will be added to the cohort until 3 subjects per cohort complete 28 days of ELI-002 therapy.

4.6.5. Rationale for Endpoints

The safety and tolerability endpoints to define the MTD (in the event there is an MTD), to define the RP2D, and to characterize the incidence of AEs and clinically significant changes laboratory tests are standard endpoints that are used in many trials of immune-therapy agents including with CAR T cells that exhibit similar mechanism of action to ELI-002. For example, CD19 CAR T cells were initially evaluated with the standard 3+3 design [Lee et al, 2015] as were BCMA CAR T cells [Raje, 2019]. Important immune related adverse events (IRAEs) may occur late with immune-oncology products. For example, the median time to onset of IRAEs when the checkpoint inhibitor nivolumab was administered in 576 subjects with advanced metastatic melanoma was 5.0 weeks for skin toxicities and 15.0 weeks for renal toxicities [Weber et al, 2017]. Therefore, the safety evaluation committee will take into account not only the MTD evaluation but rather the totality of all safety observations obtained to that point in the conduct of the trial including any serious toxicities occurring beyond the DLT evaluation window when making dose escalation decisions and when considering the definition of the RP2D.

Median RFS and median OS are exploratory endpoints that will provide preliminary data that may show a trend for a dose response in an outcome measure suitable to support the design of pivotal trials in the adjuvant setting. For example, DFS has been used as the primary endpoint supporting the approval of adjuvant breast cancer hormonal therapy, adjuvant chemotherapy for CRC, and adjuvant chemotherapy for breast cancer [Guidance for Industry, Clinical Trial Endpoints, 2018], and OS may be suitable in populations where there is a short interval between progression and death.

Exploratory objectives are intended to confirm the preliminary efficacy of ELI-002, and to provide evidence of a dose response in pharmacodynamic activities that may show when the optimal biologic dose has been achieved. Minimal residual disease response can provide a preliminary proof of concept for efficacy if ELI-002 results in reduction/clearance of ctDNA and/or serum tumor biomarkers. Emerging data using the ctDNA assay have shown a high median lead time for ctDNA changes relative to radiographic relapse and very high positive predictive value for in colorectal and NSCLC [Abbosh et al, 2017; Reinert et al, 2019] resulting in an FDA Breakthrough Device Designations in 2019 and 2021. Efficacy biomarkers CA-125, CEA, and CA19-9 will also be characterized where appropriate. In addition, exploratory objectives are included to document that ELI-002 induces the intended immune responses. T cell responses in PBMCs will be assessed by immune assays such as IFNγ, Fluorospot, intracellular cytokine staining (ICS), and/or dextramers. Patient reported outcomes (PROs) will be characterized to explore the potential symptomatic benefit of immunotherapy in protocol population. The PROs used are European Organization for Research and Treatment of Cancer (EORTC) Quality of Life Questionnaire (QLQ)-C30 for general health, and for specific tumor types, QLQ-PAN26 for pancreatic, QLQ-CR29 for colorectal, QLQ-OV28 for ovarian carcinoma, QLQ-BIL21 for biliary cancer, or QLQ-LC13 for NSCLC.

4.6.6. Rationale for Dose Selection

The clinical starting dose is based on prior clinical experience with both KRAS peptides and CpG-7909 and is supported by nonclinical data. In prior vaccine trials with mKRAS peptides, doses of peptides ranging from 100 to 5000 µg have been used, both as single peptides and in combination. Therefore the 700 µg per Amph-Peptide, 1400 µg total Amph-Peptide dose in this study is within the range previously evaluated in other clinical trials. Although other adjuvants were used in the previously conducted KRAS vaccine clinical trials, soluble CpG-7909 has been administered SC in other trials in cancer subjects at total doses up to at least 40 mg and on a body weight basis as high as 0.48 mg/kg [Melisi et al, 2014]. The dose-ranging Study No. 2795-001 (Section 4.4.1.1) also strongly supports the starting dose being proposed in this trial.

The administration of 48 µg Amph-Peptide with either 5 or 25 µg Adjuvant was well tolerated in the rodent Study No. 2795-002 (Section 4.4.1.2) with a dose-dependent reduced incidence and severity of local irritation and limited associated effects. Therefore, the NOAEL was 48 µg Amph-Peptide/25 µg Adjuvant.

The 3 planned dose levels for this trial are Amph-CpG-7909 0.1 mg SC, 0.5 mg SC, and 2.5 mg SC in combination with a fixed dose of 2 Amph-Peptides (700 µg each).

5. TRIAL OBJECTIVES

5.1. Primary Objectives

The primary objectives of Phase 1 are:

- To assess the safety and tolerability of ELI-002 as adjuvant therapy for subjects with KRAS mutated PDAC and other solid tumors who have MRD identified using either ctDNA or a serum tumor biomarker
- To define the MTD (in the event there is an MTD) and the RP2D

5.2. Secondary Objective

The secondary objective of Phase 1 is:

- To assess ctDNA reduction and clearance, defined as the reduction or clearance of ctDNA compared to baseline, or if ctDNA was not detectable at baseline, serum tumor biomarker (such as CA19-9, CEA, and CA-125) reduction and clearance compared to baseline.

5.3. Exploratory Objectives

The exploratory objectives of Phase 1 are:

- To report the median RFS and median OS
- To assess the change relative to baseline in:
  - Serum cytokines interleukin (IL)-2, IFNγ, IL-6, IL-10, and tumor necrosis factor alpha (TNFα)
  - PROs: EORTC QLQ-C30 and QLQ-PAN26, QLQ-CR29, QLQ-OV28, QLQ-BIL21, or QLQ-LC13
  - To evaluate the immunogenicity of ELI-002. T cell responses to ELI-002 will be assessed using assays such as ICS, Fluorospot, and/or dextramer+ T cells for subjects with HLA alleles for which dextramer/s is/are available
  - Biomarker levels (ctDNA, CA19-9, CEA, and CA-125)
  - To evaluate tumor-infiltrating T cells and the tumor microenvironment after dosing with ELI-002
- To correlate biomarker data to clinical safety and efficacy (RFS, OS)

6. INVESTIGATIONAL PLAN

6.1. Overall Trial Design

ELI-002-001 is an open-label, Phase 1 trial of ELI-002 immunotherapy as adjuvant treatment for subjects with KRAS/NRAS mutated PDAC and other solid tumors who are at high risk for relapse (ie, presence of isolated tumor cells as detected by ctDNA or elevated serum tumor biomarkers in the subject's body, in which the primary tumor has been removed and is currently without clinical signs of disease). High relapse risk is defined by either a ctDNA-positive result or an elevated serum tumor biomarker (such as CA19-9, CEA, or CA-125).

This Phase 1 study comprises an open label, single arm, dose-escalation trial incorporating a 3+3 design in subjects with mKRAS/mNRAS solid tumors (PDAC, CRC, NSCLC, ovarian, CCA, and gallbladder carcinoma).

Approximately 18 subjects are planned to be enrolled in the study to evaluate the safety, tolerability and exploratory pharmacodynamic efficacy of SC ELI-002 using the adjuvant at 3 planned dose levels (Amph-CpG-7909 0.1 mg SC, 0.5 mg SC, 2.5 mg SC) in combination with a fixed dose of Amph-Peptides (700 µg each). Additional cohorts may be added to explore intermediate or higher dose levels. This will be based on the cumulative safety review and preliminary review of efficacy. If additional cohorts are added, this will be documented in a study memo sent to all clinical sites and submitted to IRBs, per IRB guidance.

During the study, ELI-002 will be comprised of 1.4 mg of 2 Amph modified KRAS peptides, Amph-G12D, and Amph-G12R (0.7 mg/peptide) admixed with Amph-CpG-7909 to provide ELI-002 2P. Elicio plans to investigate the use of an Amph-Peptide 7P drug product containing all 7 Amph-Peptides (G12D, G12R, G12V, G12A, G12C, G12S, G13D) admixed with Amph-CpG-7909 (ELI-002 7P) in future clinical trials.

The ELI-002-001 protocol is an open label, single arm, dose-escalation trial incorporating a 3+3 design. Subjects will receive the initial dose of ELI-002 and be monitored for ≥24 hours in order to monitor for potential AEs such as cytokine release syndrome (CRS). In addition, there will also be a 1-week stagger between enrollment of the first subject in a cohort and the subsequent subjects. In the absence of toxicity during the DLT assessment window, the ELI-002 dose will be escalated. Planned cohort dose levels are listed below:

- Cohort 1 Dose Level 1: Amph-CpG-7909 0.1 mg with Amph-Peptides
- Cohort 2 Dose Level 2: Amph-CpG-7909 0.5 mg with Amph-Peptides
- Cohort 3 Dose Level 3: Amph-CpG-7909 2.5 mg with Amph-Peptides

Phase 1 cohorts will consist of 3 to 6 subjects (the number will depend on whether DLTs are observed). In order to ensure an adequate number of evaluable subjects, up to 6 subjects may be enrolled per cohort even in the absence of a DLT. Once the RP2D is determined, the cohort evaluating the RP2D will be expanded as needed so that there is safety experience with at least 6 subjects at this dose level.

Dose escalation from one cohort to the next will be determined by the SMC, and will be based on treatment-emergent AEs, clinical laboratory data, PE findings including vital signs, after all subjects within a cohort have completed 28 days. At each SMC meeting, longer follow-up will

be available on all but the final subject in each cohort. The SMC will review all available long-term safety information from prior cohorts (if any), so that the SMC will consider the totality of the safety information in their recommendation.

The trial will consist of:

- A Screening Period in which study specific procedures, including eligibility determination, will begin at Visit 1, following surgical resection.
- An Immunization Period which will consist of 6 SC injections: 4 weekly injections (Visit 3 to Visit 6) followed by 2 injections every two weeks at Visit 7 and Visit 8.
- A No Dosing 3-Month Period in which subjects will continue to be observed and evaluated.
- A Booster Period, consisting of 4 weekly injections (Visit 12 to Visit 15), in which eligible subjects will receive further lymph node antigen exposure.
- A Follow-up Period which will provide continued safety and efficacy data.

All subjects will be observed for safety and tolerability. Computed tomography (CT) with contrast or magnetic resonance (MRI) imaging will occur at screening and at additional timepoints indicated in the Schedule of Assessments, Table 13 and Table 15, until confirmed radiographic relapse is observed.

The central laboratory will confirm that at least 1 of the 2 mKRAS/NRAS alleles targeted by ELI-002 2P (G12D and G12R) is present and either ctDNA is positive or there are successive rising values of serum biomarker (such as CA19-9, CEA, or CA-125) from samples taken at ≥21 days post-surgery or after the last administration of adjuvant treatment (whichever comes last in the specific subject treatment plan). These data are required for determination of eligibility and inclusion into the trial.

Immunologic biomarkers of response may include HLA typing, serum cytokines, ICS, Fluorospot, and/or dextramer+ T cells for subjects with appropriate HLA alleles will be determined when matching dextramer/s is/are available.

Local laboratories will be utilized for serum cytokine testing, tumor biomarkers CA19-9/CEA/CA-125, tumor biopsy assays (such as biomarker sequencing and/or IHC assays) and all other safety laboratory tests.

Central laboratories will be utilized for ctDNA analysis, HLA typing, PBMC isolation and immunogenicity assessment, such as Fluorospot, ICS, and/or T cell dextramer testing as detailed in the central laboratory manual.

While HLA testing will be performed prior to first dose at Visit 3, cohorts will be enrolled regardless of the subject HLA subtype.

Discontinuation of trial treatment: Subjects with confirmed radiographic relapse (using iRECIST criteria; not judged as pseudoprogression) during treatment with ELI-002 will discontinue trial treatment but should continue other trial-related procedures in the follow-up period. The ORR using iRECIST criteria will be characterized for any cross-over subjects.

The design of the trial is outlined in Figure 2 and Figure 3.

Figure 2: ELI-002 Trial Cohort Schematic

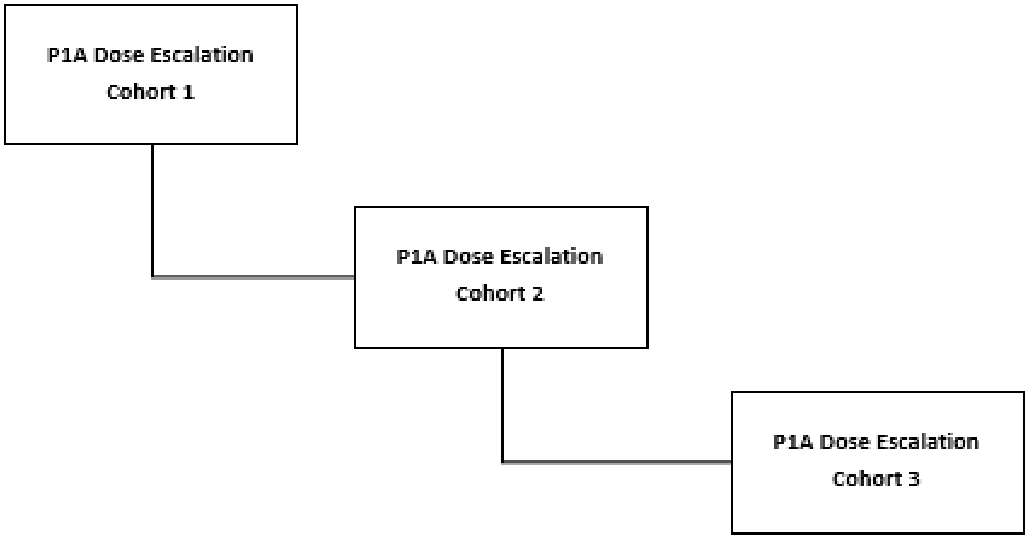

All cohorts will enroll PDAC, CRC, NSCLC and OST (including ovarian, bile duct and gallbladder carcinoma.

Figure 3: Protocol ELI-002-001: Phase 1 Trial Design

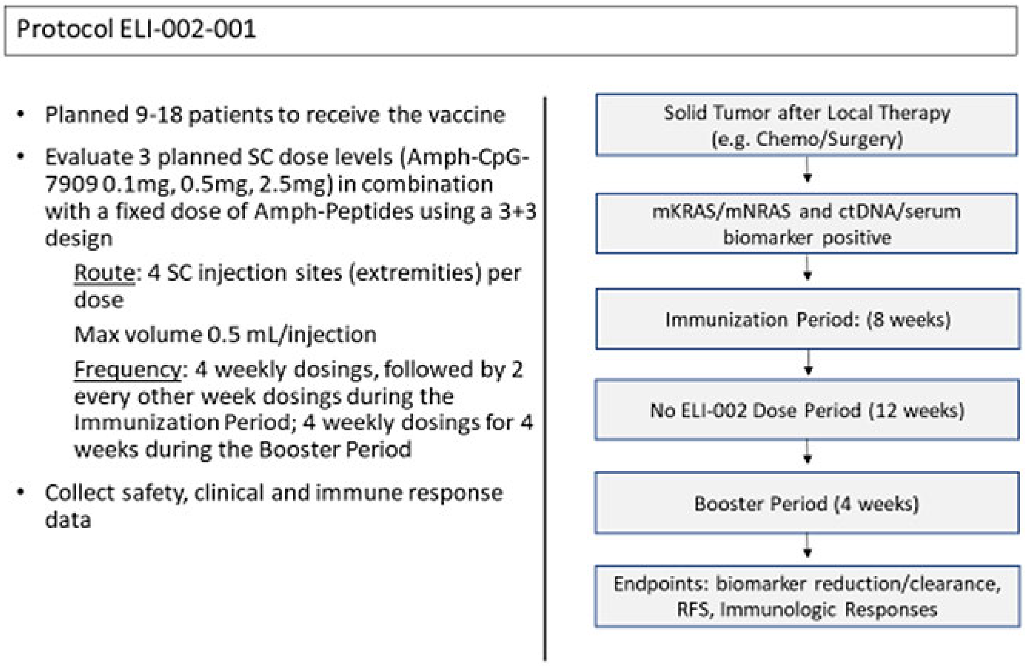

ctDNA=circulating tumor deoxyribonucleic acid; KRAS=Kirsten rat sarcoma; RFS=relapse-free survival; SC=subcutaneous

6.1.1. Safety Monitoring

This is an open label, single arm, dose-escalation trial incorporating a 3+3 design. Approximately 18 subjects are planned to be enrolled to evaluate the safety, tolerability and exploratory pharmacodynamic efficacy of ELI-002 at 3 planned dose levels (0.1 mg SC, 0.5 mg SC, 2.5 mg SC) in combination with a fixed dose of Amph-Peptides (700 µg each). Additional cohorts may be added to explore intermediate or higher dose levels. This will be based on the cumulative safety review and preliminary review of efficacy. If additional cohorts are added, this will be documented in a study memo sent to all clinical sites and submitted to IRBs, per IRB guidance.

During the study, ELI-002 will be comprised of 1.4 mg of 2 Amph modified KRAS peptides, Amph-G12D, and Amph-G12R (0.7 mg/peptide) admixed with Amph-CpG-7909 to provide ELI-002 2P. Elicio plans to investigate the use of an Amph-Peptide 7P drug product containing all 7 Amph-Peptides (G12D, G12R, G12V, G12A, G12C, G12S, G13D) admixed with Amph-CpG-7909 (ELI-002 7P) in future clinical trials.

Subjects will receive the initial dose of ELI-002 and be monitored for ≥24 hours in order to monitor for potential AEs such as CRS. In addition, there will be a 1-week stagger between enrollment of the first subject in a cohort and the subsequent subjects. In the absence of toxicity during the dose-limiting toxicity (DLT) assessment window, ELI-002 will be escalated.

Cohorts will consist of 3 to 6 subjects (the number will depend on whether DLTs are observed). In order to ensure an adequate number of evaluable subjects, up to 6 subjects may be enrolled per cohort even in the absence of a DLT.

Dose escalation from one cohort to the next will be determined by the SMC, and will be based on treatment-emergent AEs, clinical laboratory data, PE findings including vital signs, after all subjects within a cohort have completed 28 days.

In any cohort, if none of the subjects experience a DLT during the first 28 days following first administration of ELI-002, the dose escalation will occur, and 3 subjects will be enrolled in the cohort at the next dose level. However, if 1 of the 3 or 4 initial subjects in a cohort experiences a DLT, then 3 (or 2, if 4 were originally enrolled) additional subjects will be enrolled at the same dose level. If 2 or more of 3 subjects within a cohort experience a DLT, then this dose will be considered the toxic dose. If only 1 of 6 subjects within a cohort experiences a DLT during the first 28 days of treatment, then the next cohort may begin enrollment. If 2 or more of 6 subjects within a cohort experience a DLT during the first 28 days, then this dose will be considered the toxic dose. The trial may proceed at a lower dose, at a less frequent schedule, or omitting the Amph-CpG-7909 adjuvant while continuing to administer the Amph-Peptides, based on emerging toxicity or pharmacodynamic data until the MTD is determined.

A trial memo will be sent to all sites following each SMC meeting summarizing the decision; if additional cohorts are to be enrolled, the dose level and schedule will be provided.

Safety monitoring is further detailed in the SMC Charter.

The RP2D will be defined in consideration of the MTD (if any), safety data, and pharmacodynamic data. The RP2D will be communicated to all sites in a trial memo following the last SMC cohort review. Once the RP2D is determined, the cohort evaluating the Phase 2 dose will be expanded as needed so that there is safety experience with at least 6 subjects at this dose level.

Please refer to Section 6.7 for management of study drug-related events. DLT Guidelines are specifically detailed in Section 6.7.3.

6.1.2. Criteria for Booster and Additional Booster Vaccines

Accumulating clinical evidence indicates some subjects treated with an immunotherapy, such as ELI-002, may be assessed as having disease progression by conventional response criteria before demonstrating clinical objective responses and/or stable disease [Wolchok et al, 2009]. This pseudoprogression has been observed in the ELI-002-001 trial. Two hypotheses have been suggested to explain this phenomenon. The first is that enhanced inflammation within the lesions could lead to an increase in the lesion size which would appear as enlarged index lesions and therefore as newly visible small non-index lesions. Over time, both the malignant and inflammatory portions of the lesion(s) may then decrease leading to overt signs of clinical improvement. The second hypothesis is that, in some subjects, the speed of the lesion growth may initially outpace the anti-tumor immune activity of the therapy. With sufficient time, the anti-tumor activity will dominate and become clinically apparent. Therefore, subjects participating on ELI-002-001 will be allowed to continue ELI-002 treatment, as specified in this protocol, after initial investigator assessed progression (iUPD via iRECIST) if the subject is deriving clinical benefit and tolerating the study treatment well in the judgement of the investigator. Subjects must discontinue treatment upon confirmed disease progression.

All subjects free of confirmed radiographic progression will receive booster doses unless they have unresolved toxicity. For subjects which have encountered signs of toxicity, the investigator must consult with the sponsor's medical monitor who must agree with resuming the subsequent booster vaccinations (see Section 6.7.1).

Subjects who remain in the trial until the end of the required Follow-up Period may be eligible to enroll in a separate extension protocol to continue to receive further lymph node antigen exposure to maintain effective levels of antigen-specific T-cells.

6.1.3. Screening for Human Leukocyte Antigen Subtypes

High-resolution HLA information can be helpful in analyzing immune responses. Therefore, high-resolution (ie, DNA based) HLA typing will be collected via blood sample for all subjects at pre-dose Visit 3 using the central laboratory. Cohorts will be enrolled regardless of the subject's HLA type.

6.1.4. Schedule of Assessments

The schedules of assessments are provided in Table 11 through Table 16.

Table 11: Schedule of Assessments- Screening and Observation

Assessment	Screening and Observation Period		
**Visit**	**Surgery/Last Dose of Adjuvant Treatment**	**Visit 1**	**Visit 2**
**Day (± days)**	**0**	**Day 10 (Days 1-31)**	**Day 26 (±5 days)**
Written informed consent		X[1]	
Tumor tissue and EDTA blood sample for whole exome sequencing (central laboratory)[2]		X[2]	
Pregnancy testing (urine)			X
Patient reported outcomes			X[3]
Demographics (including sex, age, race and ethnicity)			X
Height/weight			X
Record medical and medication history, including cancer history/prior treatments and current medications.			X
Review of eligibility criteria			X[4]
Physical examination			X[5]
Vital signs			X
ECG			X
ECOG			X[6]
CT with contrast			X[7]
Clinical laboratory (local laboratory)			X[8]
Serum CA-125, CEA, CA19-9 (local laboratory)			X[9]
Covid-19 viral test (local laboratory)			X[10]
ctDNA (central laboratory)[11]			X[11]
Leukapheresis-PBMC collection for immunogenicity testing (central laboratory)			X[12]
Adverse events			X

CA=carbohydrate antigen; CBC=complete blood count; CEA=carcinoembryonic antigen; CRC=colorectal cancer; CT=computed tomography; ctDNA=circulating tumor deoxyribonucleic acid; DNA=deoxyribonucleic acid; ECG=electrocardiogram; ECOG=Eastern Cooperative Oncology Group; EDTA=ethylenediaminetetraacetic acid; IV=intravenous; mKRAS=mutant Kirsten rat sarcoma; mNRAS=mutant neuroblastoma; MRI=magnetic resonance imaging; PDAC=pancreatic ductal adenocarcinoma; PBMC=peripheral blood mononuclear cell

1. The ICF has no expiration and can be signed at any time during or after the prior treatment (including surgery/resection). The screening window (56 days) begins when the first screening procedure is performed.
2. Depending on the clinical site location, a tumor and blood sample will be collected for KRAS mutation status. The tumor sample, if required, will be used for WES to determine KRAS mutation and to develop the tumor-informed personalized ctDNA test. Details are provided in the Central Laboratory Manual. Once a subject has consented, the trial coordinator will requisition a retrospective tumor tissue sample taken during surgical resection (if needed per clinical site location). Prior to shipment, a fresh 6-mL EDTA blood tube will also be collected. Tumor DNA will be sequenced for baseline tumor gene expression. The somatic DNA from the EDTA tube will serve as a control to ensure only tumor-specific DNA is included in the selection for ctDNA testing.
3. The patient reported outcomes (PROs) will include the general QLQ-C30, as well as a specific PRO for the subject's tumor type (see Section 11.3). PROs must be completed prior to other study visit procedures.
4. See Inclusion and Exclusion Criteria in Section 7.1 and Section 7.2, respectively. While mKRAS and ctDNA analyses will not be available at Visit 2, the expectation is that these will be reviewed, and full eligibility determined, prior to Visit 3.
5. Physical examinations are defined in Section 12.1.3.
6. To be eligible, ECOG performance status must be 0 to 1 (see Section 12.1.5)
7. To be eligible, post-operative CT must be negative for radiographic recurrent disease. CT imaging assessments will occur within 90 days of first ELI-002 dose, following resection or last dose of adjuvant treatment (whichever comes last in the specific subject treatment plan). For subjects with IV contrast allergy/intolerance, MRI may be performed.
8. See Inclusion Criterion #9 regarding laboratory values in Section 7.1. Safety laboratories should include CBC with differential: chemistry to include parameters listed in Section 12.1.6.3; hematology to include parameters listed in Section 12.1.6.1; coagulation to include parameters listed in Section 12.1.6.2; cytokine laboratories IL-2, IFNγ, IL-6, IL-10, and TNFα; viral testing to include parameters listed in Section 12.1.6.5. Details for central laboratory sample collections are provided in the Central Laboratory Manual.
9. See Inclusion #4 regarding serum biomarker values in Section 7.1. Biomarker values must be obtained ≥21 days post-surgery or last administration of adjuvant therapy, whichever comes last in the specific subject's treatment plan.
10. Samples such as swabs, saliva, or others as appropriate may be used for Covid-19 viral testing according to the locally available assay.
11. Blood will be collected for ctDNA analysis at a central laboratory. Eligibility requires that subject's must be MRD positive, as measured by either a ctDNA blood test persisting ≥21 days post-surgery or after last administration of adjuvant treatment (whichever is last in the specific subject treatment plan) or serum biomarker, with 1 of the 2 ELI-002-specific mKRAS/mNRAS alleles. Details for blood collection are provided in the Central Laboratory Manual.
12. The leukapheresis sample must be collected once eligibility has been confirmed and prior to the first dose of ELI-002. The PBMCs may be used for immunogenicity and/or biomarker genetic sequencing. Details for central laboratory sample collections are provided in the Central Laboratory Manual.

Table 12: Schedule of Assessments- Immunization Period

Assessment	Immunization Period						
Visit	Visit 3		Visit 4	Visit 5	Visit 6	Visit 7	Visit 8
Day (± days)	Day 57 Baseline (prior to dosing)	Day 57 (dosing) (±3 days)	Day 64 (±3 days)	Day 71 (±3 days)	Day 78 (±3 days)	Day 92 (±3 days)	Day 106 (±3 days)
Dose #	-	Dose 1	Dose 2	Dose 3	Dose 4	Dose 5	Dose 6
Patient reported outcomes	X[1]		X[1]	X[1]	X[1]	X[1]	X[1]
Review medical history	X[2]						
Review cancer history/prior treatments	X						
Physical Exam	X[3]		X[3]	X[3]	X[3]	X[3]	X[3]
Vital signs	X	X	X	X	X	X	X
Weight	X		X	X	X	X	X
ECG	X						
ECOG	X		X	X	X	X	X
Concomitant medications	X		X	X	X	X	X
Clinical laboratory (local laboratory)	X[4]		X[4]	X[4]	X[4]	X[4]	X[4]
ctDNA (central laboratory)[5]	X[5]				X[5]		
Whole blood-PBMC collection for immunogenicity testing (central laboratory)				X[4]		X[4]	
Serum collection of cytokine assay (local laboratory)	X[4]		X[4]	X[4]	X[4]	X[4]	X[4]
High Resolution HLA typing (central laboratory)	X[6]						
Trial drug (ELI-002) administration		X[7]	X[7]	X[7]	X[7]	X[7]	X[7]
Adverse events	X[8]	X[8]	X[8]	X[8]	X[8]	X[8]	X[8]
Reactogenicity diary[9]		X	X	X	X	X	X

CA=carbohydrate antigen; CBC=complete blood count; CEA=carcinoembryonic antigen; CRC=colorectal cancer; CRS=cytokine release syndrome; CT=computed tomography; ctDNA=circulating tumor deoxyribonucleic acid; ECG=electrocardiogram; ECOG=Eastern Cooperative Oncology Group: HLA=human leukocyte antigen; IFNγ=interferon gamma; IL=interleukin; mKRAS=mutant Kirsten rat sarcoma; mNRAS=mutant neuroblastoma ras viral oncogene homolog; MRD=minimal residual disease; PBMC=peripheral blood mononuclear cell; PDAC=pancreatic ductal adenocarcinoma; PE=physical examination; TNFα=tumor necrosis factor alpha

1. The patient reported outcomes (PROs) will include the general QLQ-C30, as well as a specific PRO for the subject's tumor type (see Section 11.3). PROs must be completed prior to other study visit procedures.
2. Subjects must have recovered from surgery without any ongoing medical/surgical issues. See Inclusion and Exclusion Criteria in Section 7.1 and Section 7.2, respectively.
3. Physical examinations are defined in Section 12.1.3. In addition, a neurological examination for baseline ICANS/ICE assessment will be performed at Visit 3, prior to

dosing.

4. Safety laboratories should include CBC with differential: chemistry to include parameters listed in Section 12.1.6.3; hematology to include parameters listed in Section 12.1.6.1; coagulation to include parameters listed in Section 12.1.6.2. Cytokine laboratories IL-2, IFNγ, IL-6, IL-10, and TNFα; this testing should be performed and reviewed within 7 days prior to dosing. The PBMCs may be used for immunogenicity and/or biomarker genetic sequencing. Details for central laboratory sample collections are provided in the Central Laboratory Manual.
5. ctDNA blood testing will occur at a central laboratory. Details for blood collection are provided in the Central Laboratory Manual
6. High resolution HLA will be performed for all subjects, prior to first dose at Visit 3. Cohorts will be enrolled regardless of the HLA subtype (see Section 6.1.3 for details). Details for blood collection are provided in the Central Laboratory Manual.
7. ELI-002 administration: Subjects will receive the initial dose of ELI-002 and be monitored for ≥24 hours in order to monitor for potential adverse events such as CRS. For subsequent doses, subjects must be observed for safety for at least 1-hour postdose.
8. Check for signs of toxicity. See Section 6.7 for instructions regarding signs of toxicity and for criteria for stopping treatment administration.
9. Subjects will be provided with a paper reactogenicity diary at the end of the visit. They will be instructed to fill out the diary each day, for 7 days, at the same time per the instructions on the study template. Subjects will return the completed diary at the next scheduled visit. The site staff will review the diary with the subject and file in the subject study records. This completed diary will be used as source data for eCRF data entry.

Table 13: Schedule of Assessments- No Dosing 3-Month Period

Assessment	No Dosing 3-Month Period		
Visit	Visit 9	Visit 10	Visit 11
Day (± days)	Day 120 (±3 days)	Day 148 (±3 days)	Day 176 (±3 days)
Patient reported outcomes	X[1]	X[1]	X[1]
Physical examination	X[2]	X[2]	X[2]
Vital signs	X	X	X
Weight	X	X	X
Changes in concomitant medications	X	X	X
CT with contrast		X[3]	
Clinical laboratory (local laboratories)	X[4]	X[4]	X[4]
ctDNA (central laboratory)[5]			X[5]
Leukapheresis-PBMC collection for immunogenicity testing (central laboratory)	X[6]		
Whole blood-PBMC collection for immunogenicity testing (central laboratory)			X[7]
Serum collection for cytokine (local laboratory)			X[4]
Serum CA-125/CEA/CA 19-9 (local laboratories)		X[4]	
Standard of care biopsy (local laboratory)		X[8]	
Adverse events	X	X	X

CA=carbohydrate antigen; CBC=complete blood count; CEA=carcinoembryonic antigen; CRC=colorectal cancer; CRP=C-reactive protein; CT=computed tomography; ctDNA=circulating tumor deoxyribonucleic acid; IFNγ=interferon gamma; IL=interleukin; IV=intravenous; mKRAS=mutant Kirsten rat sarcoma; mNRAS=mutant neuroblastoma ras viral oncogene homolog; MRI=magnetic resonance imaging; PBMC=peripheral blood mononuclear cell TNFα =tumor necrosis factor alpha

1. The patient reported outcomes (PROs) will include the general QLQ-C30, as well as a specific PRO for the subject's tumor type (see Section 11.3). PROs must be completed prior to other study visit procedures.
2. Physical examinations are defined in Section 12.1.3.
3. A visit window of ±14 days is permitted for imaging assessments. For subjects with IV contrast allergy/intolerance, MRI may be performed. Unscheduled CT or MRI imaging may be performed at any time, if the investigator determines that there are clinical signs of disease progression.
4. Safety laboratories should include CBC with differential: chemistry to include parameters listed in Section 12.1.6.3; hematology to include parameters listed in Section 12.1.6.1; coagulation to include parameters listed in Section 12.1.6.2. Cytokine laboratories IL-2, IFNγ, IL-6, IL-10, and TNFα. Serum biomarkers CA-125 [ovarian], CA19-9 [PDAC] or CEA [CRC]. Details for central laboratory sample collections are provided in the Central Laboratory Manual
5. ctDNA blood testing will occur at a central laboratory. Details for blood collection are provided in the Central Laboratory Manual
6. The leukapheresis sample may be collected -7 days or +3 days from Visit 9. The PBMCs may be used for immunogenicity and/or biomarker genetic sequencing. Details for central laboratory sample collections are provided in the Central Laboratory Manual.

7. The PBMCs may be used for immunogenicity and/or biomarker genetic sequencing. Details for central laboratory sample collections are provided in the Central Laboratory Manual.
8. If new lesions are observed on radiographic imaging, a standard of care biopsy may be performed per iRECIST criteria to confirm disease progression and when the investigator judges that tissue can be safely obtained. If there is sufficient tissue obtained from the biopsy, the pathology lab should perform in situ gene expression and/or IHC for evaluation of tumor infiltrating T cells and the tumor microenvironment.

Table 14: Schedule of Assessments- Booster Period

Assessment	Booster Period			
**Visit**	**Visit 12**	**Visit 13**	**Visit 14**	**Visit 15/ End of Treatment**
**Day (± days)**	**Day 196 (±3 days)**	**Day 203 (±3 days)**	**Day 210 (±3 days)**	**Day 217 (±3 days)**
**Dose #**	**Dose 7**	**Dose 8**	**Dose 9**	**Dose 10**
Patient reported outcomes	X[1]	X[1]	X[1]	X[1]
Physical examination	X[2]	X[2]	X[2]	X[2]
Vital signs	X	X	X	X
Weight	X	X	X	X
ECOG	X	X	X	X
Concomitant medications	X	X	X	X
Clinical laboratory (local laboratory)	X[3]	X[3]	X[3]	X[3]
ctDNA (central laboratory)[4]	X[4]			
Whole blood-PBMC collection for immunogenicity testing (central laboratory)			X[5]	
Serum collection for cytokine assay (local laboratory)	X[3]	X[3]	X[3]	X[3]
Trial drug (ELI-002) administration	X[6]	X[6]	X[6]	X[6]
Adverse events	X[7]	X[7]	X[7]	X[7]
Reactogenicity diary[8]	X	X	X	X

CA=carbohydrate antigen; CBC=complete blood count; CEA=carcinoembryonic antigen; CRC=colorectal cancer; ctDNA=circulating tumor deoxyribonucleic acid; ECOG=Eastern Cooperative Oncology Group; IFNγ=interferon gamma; IL=interleukin; IV=intravenous; mKRAS=mutant Kirsten rat sarcoma; mNRAS=mutant neuroblastoma ras viral oncogene homolog; MRI=magnetic resonance imaging; PBMC=peripheral blood mononuclear cell; PDAC=pancreatic ductal adenocarcinoma; TNFα =tumor necrosis factor alpha

1. The patient reported outcomes (PROs) will include the general QLQ-C30, as well as a specific PRO for the subject's tumor type (see Section 11.3). PROs must be completed prior to other study visit procedures.
2. Physical examinations are defined in Section 12.1.3.
3. Safety laboratories should include CBC with differential: chemistry to include parameters listed in Section 12.1.6.3; hematology to include parameters listed in Section 12.1.6.1; coagulation to include parameters listed in Section 12.1.6.2. Cytokine laboratories TNFα =tumor necrosis factor alpha IL-2, IFNγ, IL-6, IL-10, and TNFα; this testing should be performed and reviewed within 7 days prior to dosing. Details for central laboratory sample collections are provided in the Central Laboratory Manual
4. ctDNA blood testing will occur at a central laboratory. Details for blood collection are provided in the Central Laboratory Manual
5. The PBMCs may be used for immunogenicity and/or biomarker genetic sequencing. Details for central laboratory sample collections are provided in the Central Laboratory Manual

6. ELI-002 administration for those subjects in Phase 1. Subjects must be observed for safety for at least 1-hour postdose.
7. Check for signs of toxicity. See Section 6.7 for instructions regarding signs of toxicity and for criteria for stopping treatment administration.
8. Subjects will be provided with a paper reactogenicity diary at the end of the visit. They will be instructed to fill out the diary each day, for 7 days, at the same time per the instructions on the study template. Subjects will return the completed diary at the next scheduled visit. The site staff will review the diary with the subject and file in the subject study records. This completed diary will be used as source data for eCRF data entry.

Table 15: Schedule of Assessments- Follow-up Period (Year 1 to Year 2)

Assessment	Follow-up Period								
Visit	Visit 16	Visit 17	Visit 18	Visit 19	Visit 20	Visit 21	Visit 22	Visit 23	Visit 24
Day (± days)	Day 231 (±3 days)	Day 259 (±3 days)	Day 315 (±3 days)	Day 399 (±3 days)	Day 483 (±3 days)	Day 567 (±3 days)	Day 651 (±3 days)	Day 735 (±3 days)	Day 819 (±3 days)
Patient reported outcomes	X[1]	X[1]	X[1]	X[1]	X[1]	X[1]	X[1]	X[1]	X[1]
Physical Exam					X[2]				
Vital signs	X	X	X	X	X	X	X	X	X
Weight	X	X	X	X	X	X	X	X	X
ECOG	X	X	X	X	X	X	X	X	X
CT with contrast		X[3]		X[3]	X[3]	X[3]	X[3]	X[3]	X[3]
Clinical laboratory (local laboratory)	X[4]								
ctDNA (central laboratory)[5]	X[5]	X[5]	X[5]	X[5]	X[5]	X[5]	X[5]	X[5]	X[5]
Whole blood-PBMC collection for immunogenicity testing (central laboratory)	X[6]						X[6]		
Serum CA-125/CEA/CA 19-9 (local laboratory)		X[4]		X[4]	X[4]	X[4]	X[4]	X[4]	X[4]
Serum collection for cytokine analysis (local laboratory)	X[4]								
Adverse events	X[7]	X[7]	X[7]	X[7]	X[7]	X[7]	X[7]	X[7]	X[7]
Standard of care biopsy (local laboratory)		X[8]		X[8]	X[8]	X[8]	X[8]	X[8]	X[8]

CA=carbohydrate antigen; CBC=complete blood count; CEA=carcinoembryonic antigen; CRC=colorectal cancer; ctDNA=circulating tumor deoxyribonucleic acid; ECOG=Eastern Cooperative Oncology Group; IFNγ=interferon gamma; IL=interleukin; IV=intravenous; mKRAS=mutant Kirsten rat sarcoma; mNRAS=mutant neuroblastoma ras viral oncogene homolog; MRI=magnetic resonance imaging; PBMC=peripheral blood mononuclear cell; PDAC=pancreatic ductal adenocarcinoma; TNFα =tumor necrosis factor alpha

1. The patient reported outcomes (PROs) will include the general QLQ-C30, as well as specific PROs for the subject's tumor type (see Section 11.3). PROs must be completed prior to other study visit procedures.
2. Physical examinations are defined in Section 12.1.3.
3. A visit window of ±14 days is permitted for imaging assessments during the Follow-up Period. For subjects with IV contrast allergy/intolerance, MRI may be performed. Unscheduled CT or MRI imaging may be performed at any time, if the investigator determines that there are clinical signs of disease progression.
4. Safety laboratories should include CBC with differential: chemistry to include parameters listed in Section 12.1.6.3; hematology to include parameters listed in Section 12.1.6.1; coagulation to include parameters listed in Section 12.1.6.2. Cytokine laboratories IL-2, IFNγ, IL-6, IL-10, and TNFα. Serum biomarkers CA-125 [ovarian], CA19-9 [PDAC] or CEA [CRC]. Details for central laboratory sample collections are provided in the Central Laboratory Manual
5. ctDNA blood testing will occur at a central laboratory. Details for blood collection are provided in the Central Laboratory Manual

6. The PBMCs may be used for immunogenicity and/or biomarker genetic sequencing. Details for central laboratory sample collections are provided in the Central Laboratory Manual.
7. Long-term observation for post dose vaccination adverse events will be recorded if an adverse event began within 30 days of last dose of treatment and continues in the follow-up period. See Section 6.7 for instructions regarding signs of toxicity.
8. If new lesions are observed on radiographic imaging, a standard of care biopsy may be performed per iRECIST criteria to confirm disease progression and when the investigator judges that tissue can be safely obtained. If there is sufficient tissue obtained from the biopsy, the pathology lab should perform in situ gene expression and/or IHC for evaluation of tumor infiltrating T cells and the tumor microenvironment.
9. All enrolled subjects should be followed in the Follow-Up period for disease status and overall survival. As part of the study, sites may conduct searches of public records, such as those establishing overall survival status, to obtain survival data as needed (ie: a subject that is lost to follow-up, withdraws from the study, etc.)

Table 16: Schedule of Assessments- Follow-up Period (Year 2 to Year 3)

Assessment	Follow-up Period			
**Visit**	**Visit 25**	**Visit 26**	**Visit 27**	**Visit 28/ End of Study[6]**
**Day (± days)**	**Day 903 (±3 days)**	**Day 987 (±3 days)**	**Day 1071 (±3 days)**	**Day 1127 (±3 days)**
ECOG	X	X	X	X
CT with contrast	X[1]	X[1]	X[1]	X[1]
ctDNA (central laboratory)	X[3]	X[3]	X[3]	X[3]
Serum CA-125/CEA/CA 19-9 (local laboratory)	X[2]	X[2]	X[2]	X[2]
Adverse events	X[4]	X[4]	X[4]	X[4]
Standard of care biopsy (local laboratory)	X[5]	X[5]	X[5]	X[5]

CA=carbohydrate antigen; CEA=carcinoembryonic antigen; CRC=colorectal cancer; CT=computed tomography; ctDNA=circulating tumor deoxyribonucleic acid; ECOG=Eastern Cooperative Oncology Group; IV=intravenous; MRI=magnetic resonance imaging; PDAC=pancreatic ductal adenocarcinoma;

1. A visit window of ±14 days is permitted for imaging assessments during the Follow-up Period. For subjects with IV contrast allergy/intolerance, MRI may be performed. Unscheduled CT imaging or MRI may be performed at any time, if the investigator determines that there are clinical signs of disease progression.
2. Serum biomarkers CA-125 [ovarian], CA19-9 [PDAC] or CEA [CRC]. Details for central laboratory sample collections are provided in the Central Laboratory Manual
3. ctDNA blood testing will occur at a central laboratory. Details for blood collection are provided in the Central Laboratory Manual
4. Long-term observation for post dose vaccination adverse events will be recorded if an adverse event began within 30 days of last dose of treatment and continues in the follow-up period. See Section 6.7 for instructions regarding signs of toxicity.
5. If new lesions are observed on radiographic imaging, a standard of care biopsy may be performed per iRECIST criteria to confirm disease progression and when the investigator judges that tissue can be safely obtained. If there is sufficient tissue obtained from the biopsy, the pathology lab should perform in situ gene expression and/or IHC for evaluation of tumor infiltrating T cells and the tumor microenvironment.
6. All enrolled subjects should be followed in the Follow-Up period for disease status and overall survival. As part of the study, sites may conduct searches of public records, such as those establishing overall survival status, to obtain survival data as needed (ie: a subject that is lost to follow-up, withdraws from the study, etc.)

6.1.5. Risk Determination for Investigational In Vitro Diagnostics

Protocol ELI-002-001 incorporates two different investigational in vitro diagnostics (IVDS). Only one IVD will be used for each subject's enrollment, based on ability to access acceptable tumor sample and/or upon Elicio's instruction.

1. Tissue-informed personalized ctDNA assay: This approach requires two assays, a tissue based Comprehensive Genomic Profiling (CGP) test based on WES (KRAS mutation) followed by a personalized ctDNA assay (MRD status). Both assays are performed at central laboratories under CLIA and CAP requirements.
2. Plasma RAS ctDNA assay: This approach requires a single ctDNA assay to monitor both KRAS mutation and MRD status performed at a central laboratory under CLIA and CAP requirements.

For the tumor-informed personalized ctDNA assay, to determine eligibility, subjects will have their tumor specimen assayed using CGP-WES to confirm presence of at least 1 of the mKRAS/mNRAS alleles targeted by ELI-002. Next, the WES data will be used to identify subject-specific somatic variants and design a personalized assay, per subject, for the detection of ctDNA in plasma (see Figure 4).

For the plasma RAS ctDNA assay, to determine eligibility, subjects will proceed to have their blood tested to confirm the presence of at least 1 of the mKRAS/mNRAS alleles targeted by ELI-002 and the detection of ctDNA in plasma in a single assay (see Figure 4).

Figure 4: Flow Diagram of ELI-002-001 Eligibility Determination using the IVD assays

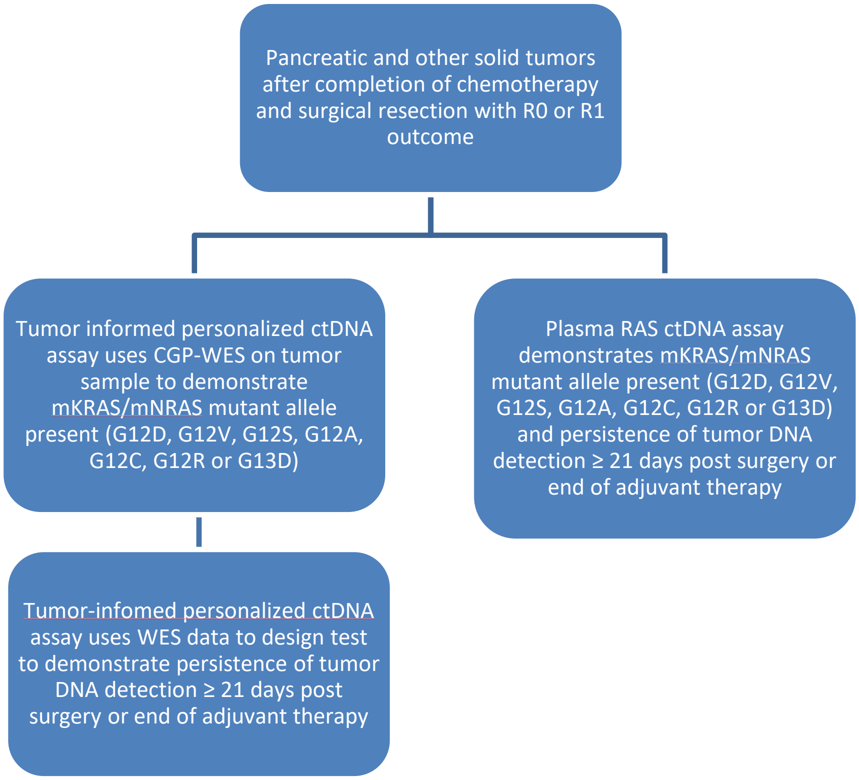

Subjects eligible to participate must have at least 1 of the mKRAS/mNRAS alleles targeted by ELI-002 and they must have either detectable ctDNA or a successively rising serum tumor biomarker (CA19-9, CEA, or CA-125) in samples taken ≥21 days after surgery or after the last administration of adjuvant therapy, whichever comes last in the subject's treatment plan. The ctDNA status is determined by the investigation IVDs.

Elicio considers the use of ctDNA investigational IVDs in Protocol ELI-002-001 to be nonsignificant risk. The planned administration of ELI-002 2P occurs after standard surgery and neoadjuvant and/or adjuvant chemotherapy and before relapse characterized by the re-emergence of clinical or radiographic symptoms. As there is no standard of care treatment for minimal residual disease in this space, a false positive or false negative will not result in a substitution or omission of standard treatment.

6.1.5.1. Description of the IVDs

6.1.5.1.1. Tumor-informed Personalized ctDNA Assay

The tumor informed personalized ctDNA assay is composed of two parts, WES from tumor DNA to identify tumor-specific mutations and a plasma-based ctDNA assay to detect ctDNA.CGP-WES test is a CAP- and CLIA-validated Laboratory Developed Test, performed at a single-site laboratory. The assay compares the whole exome sequence of tumor and germline DNA, facilitating identification of tumor-specific DNA alterations which for the ELI-002-001 trial will be analyzed to identify the specific subset of subjects with mKRAS/mNRAS alleles for

eligibility. The assay is performed by target enrichments followed by 440x coverage sequencing on an Illumina HiSeq 2500 or NovaSeq 6000 (Illumina, Inc.). Use of CGP-WES to identify mKRAS alleles has been documented in pancreatic [Liang et al, 2012] and colon cancer [Shanmugam et al, 2014].

The tissue-based CGP test is intended for inclusion of subjects who are positive for any of the specific mutations (eg, G12Dand G12R) within the KRAS or NRAS genes that are targeted by the ELI-002 product candidate. In addition, the WES data will serve as the input for subject specific personalized Signatera ctDNA assay.

The Natera Signatera test is a personalized multiplex-PCR and NGS-based clinical assay targeting 16 tumor-specific mutations. It is intended for the detection of ctDNA isolated from anticoagulated peripheral whole blood

The standard Signatera assay is based on tracking driver clonal/truncal mutations that occur early in tumor initiation. Signatera tracks a subset of these early clonal variants to detect residual ctDNA with significantly increased sensitivity using a multiplexed PCR methodology. In brief, WES of tumor and matched normal blood are used to identify 16 clonal, somatic tumor variants not present in non-tumor tissues. Then a multiplexed PCR assay specific for a given subject assays serial plasma biospecimens – presence or absence of ctDNA is highly predictive of response to treatment or MRD [Sethi et al, 2018]. Signatera ctDNA positive status was significantly correlated with RFS across several tumor types, including mKRAS-positive colon [Reinert et al, 2019] and lung [Abbosh et al, 2017].

6.1.5.1.2. Plasma RAS ctDNA Assay

The Sysmex SafeSEQ RAS-RAF pathway assay was designed to deliver a clinical grade, ultra-sensitive liquid biopsy solution for the identification of gene mutations in KRAS, NRAS, BRAF and PIK3CA. This test identifies different genomic alterations (single and multiple base substitutions, insertions and deletions) across 24 regions in these oncogenes. It is intended for the detection of ctDNA isolated from plasma. The SafeSEQ RAS-RAF pathway assay will identify subjects who are positive for any of the specific mutations (eg, G12D and G12R) within the KRAS or NRAS genes that are targeted by the ELI-002 product candidate. In addition, the SafeSEQ assay will inform the presence or absence of ctDNA. Unlike the tumor-informed personalized assay, tumor tissue is not required for KRAS mutation identification; instead, a single plasma sample will inform both KRAS mutation type and ctDNA positivity (MRD).

SafeSEQ is an amplification-based NGS method which was designed for ctDNA analysis to suppress NGS errors and to improve the calling of low frequency mutations. This approach enables confidence in the calling of mutations from clinical specimens even with a low volume of input DNA. SafeSEQ provides nearly 100% conversion of DNA input, so that molecules, including rare ctDNA molecules, are effectively measured. This allows for high sensitivity and accuracy by discriminating sequencing artifacts from bona fide mutations. Use of SafeSEQ to identify KRAS and NRAS gene mutations has been documented in colorectal and pancreatic cancers [Bettegowda et al, 2014]. Sysmex SafeSEQ positive ctDNA detection after resection was associated with an increased risk of relapse in pancreatic [Lee, Lipton, et al, 2019] and colorectal [Tie et al, 2019] cancers.

6.1.5.2. Applying the IVD Results in the Clinical Trial

ELI-002-001 is a Phase 1, open-label trial of ELI-002 as adjuvant therapy for subjects with mKRAS/mNRAS+ solid tumors who have no standard available treatment. These subjects have undergone surgical resection and may have positive MRD as determined by detectable ctDNA. MRD will be defined as the presence of ctDNA in the subject's body, in which the primary tumor has been removed and the subject is currently without clinical signs of disease. The mKRAS/mNRAS+ status will be confirmed by CGP WES from tumor samples versus normal germline or from plasma RAS ctDNA from plasma samples, and ctDNA status will be determined by either the tumor-informed or plasma RAS ctDNA assays at screening.

Approximately 18 subjects are planned to be treated in 3 planned dose level cohorts. The RP2D will be determined by consideration of the MTD (if any MTD is observed), safety data, and pharmacodynamic data.

Baseline mKRAS/mNRAS status and ctDNA status for eligibility will be assessed during screening.

The tumor-informed personalized ctDNA or plasma RAS ctDNA assays to detect mKRAS/mNRAS and MRD status will be used to assess subject eligibility for enrollment.

The prevalence of mutations in KRAS and NRAS to be evaluated by the IVDs is reviewed in detail in protocol Section 4.1. The prevalence of NRAS mutations is highest in CRC among tumor types included in this study, while KRAS mutations are found across all histologies.

6.2. Endpoints

6.2.1. Primary Endpoints

The primary endpoints of Phase 1 are:

- To define the MTD of ELI-002 (in the event there is an MTD) and the RP2D
- To evaluate safety as assessed by the incidence of AEs and clinically significant in laboratory tests and vital signs

6.2.2. Secondary Endpoint

The secondary endpoint of Phase 1 is:

- To report the proportion of subjects with ctDNA reduction and clearance, defined as reduction or clearance in ctDNA compared to baseline, or if ctDNA was not detectable at baseline, serum tumor biomarker (such as CA19-9, CEA, and CA-125) reduction and clearance compared to baseline

6.2.3. Exploratory Endpoints

The exploratory endpoints of Phase 1 are:

- Median RFS, and median OS

- Duration of biomarker (ctDNA or serum tumor biomarker) clearance and reduction, defined as time from the date of the first negative and/or decreased biomarker to the earliest date of any of the following events:
  – Subsequent increased/positive biomarker
  – Subsequent disease relapse
  – Death due to any cause
- Change relative to baseline in:
  – Serum cytokines IL-2, IFNγ, IL-6, IL-10, and TNFα
  – PROs: EORTC QLQ-C30 and QLQ-PAN26, QLQ-CR29, QLQ-OV28, QLQ-BIL21 or QLQ-LC13
  – Immunogenicity of ELI-002 determined by magnitude of response and fold-change from baseline using assays such as ICS, Fluorospot, and/or dextramer+ T cells for subjects with HLA alleles for which dextramer/s is/are available
  – Biomarker levels (ctDNA, CA19-9, CEA, and CA-125)
  – Immune cell infiltrate and tumor microenvironment, if standard of care biopsies are obtained, to confirm disease progression

6.3. Number of Subjects

Approximately 18 subjects are planned to be enrolled in this trial: Additional cohorts may be added to explore intermediate or higher dose levels based on the cumulative safety review and preliminary review of pharmacodynamic responses. If additional cohorts are added, this will be documented in a study memo sent to all clinical sites and submitted to IRBs, per IRB guidance.

6.4. Treatment Assignment

Approximately 18 subjects are planned to receive SC injections of ELI-002 during the Immunization Period and the Booster Period.

6.5. Dose Adjustment Criteria

ELI-002 will be escalated in the planned dose increments (see Section 6.1.1).

The SMC will have the authority to assess and recommend any dosing changes warranted due to safety concerns.

There are no PK assessments in this protocol, therefore, there will be no consideration for PK criteria for adjustment or stopping of doses.

6.6. Safety Criteria for Temporarily or Permanently Stopping the Trial

The sponsor medical monitor will be continuously monitoring the safety of subjects. In addition, the cumulative safety will be reviewed by the SMC at the following key time points:

- After the last subject in each cohort completes the DLT observation period
- Ad hoc as necessary

The trial may be temporarily or permanently stopped by the sponsor, if any subject death occurs attributed to administration of ELI-002 (ie, if there is Grade 5 toxicity that is definitely, probably, or possibly attributed to trial treatment).

Any potential re-initiation of the trial will be discussed by the Sponsor, with the support of the SMC and in consultation with the FDA, if necessary.

6.7. Management of Study Drug-Related Events

6.7.1. Temporarily Suspending Treatment Administration

If a subject meets any of the following conditions within 48 hours prior to the scheduled administration of the trial treatment, treatment administration will be temporarily suspended:

1. To reduce the risk of CRS, subjects will not be given a repeat dose of ELI-002 if they are exhibiting T ≥38°C and/or IL-6 ≥500 pg/mL (cytokine collection, including IL-6, may be performed up to 7 days prior to treatment). In addition, if a subject exhibits IFNγ ≥1000 pg/mL, the Elicio medical monitor should be contacted for consultation.
2. Adverse event(s) that meet the definition of DLT caused by prior treatment. See Section 6.7.3 for the definition of a DLT; see Section 12.4 for the definition of Common Terminology Criteria for Adverse Events (CTCAEs).
3. Any subsequent dosing in a subject who has experienced Grade ≥2 CRS/ immune effector cell-associated neurotoxicity syndrome (ICANS)

Investigators must consult the medical monitor to discuss any subjects meeting the above criteria.

In the event the ELI-002 dose is held, the subject should not receive any make up dose and remain on the original protocol schedule (ie, their next dose will be at the next protocol-specified dose if they meet the criteria for dosing). All trial procedures and treatment administration will be resumed as soon as the subject’s condition allows or at the investigator’s discretion after Sponsor approval.

The trial may proceed at a lower dose, at a less frequent schedule, or omitting the Amph-CpG-7909 adjuvant while continuing to administer the Amph-Peptides, based on emerging toxicity or pharmacodynamic data until the MTD is determined.

6.7.2. Permanent Stopping Treatment Administration

If a subject meets any of the following conditions, the subject will be required to discontinue the trial treatment administration but should continue other trial-related procedures as specified for the follow-up period of the study. (See Section 7.3 for more information on procedures for subjects withdrawn from trial treatment.)

1. Evidence of radiographic disease progression
2. Any intolerable AE, at the investigator’s discretion

3. During study treatment:
   - Any investigational product use other than ELI-002
   - Other anti-cancer treatment
4. Non-compliance with trial treatments/procedures. If a subject misses ≥ 2 trial visits without underlying toxicity, the subject should be re-instructed regarding dosing and follow-up and continue trial participation. If a subject continues to miss trial visits without toxicity and despite re-instruction, they should be discontinued from the trial treatment phase but follow-up data should be collected as possible.
5. The subject requests to be withdrawn from the trial treatment

For subjects whose treatment is discontinued prematurely, the procedures outlined for End of Treatment/Visit 15 (Table 14) will be carried out and, if possible, prior to the initiation of any other anti-cancer treatment. These subjects should then enter the Follow-up Period, where procedures listed in Table 15 and Table 16 will be performed. In the event that a subject will not continue to be evaluated in the Follow-up Period, the End of Study visit (Visit 24) should be scheduled, and all procedures performed.

6.7.3. Dose-limiting Toxicity Guidelines

A DLT will be defined as any event at least possibly related to ELI-002 as follows:

a. Any Grade ≥ 3 non-hematologic toxicity except asymptomatic laboratory abnormalities (see Section 12.2.1.1)
b. Any Grade ≥ 3 toxicity involving major organ systems for greater than 72 hours and occurring within 28 days of subcutaneous administration
c. Grade 3 CRS that does not resolve to ≤ Grade 2 within 7 days
d. Any Grade 4 CRS that does not improve to < Grade 2 within 72 hours
e. Any Grade ≥ 3 autoimmune disorder
f. Any Grade ≥ 3 rash that does not resolve to ≤ Grade 1 within 7 days with appropriate treatment

The DLT observation period will be 28 days.

Maximum tolerated dose definition: The MTD is defined as the highest dose level with a DLT incidence <33% of cohort subjects.

See protocol Section 6.7.1 and Section 6.8 for management of toxicity.

6.8. Management of Risks and Toxicities

Review of the ELI-002 pre-clinical data indicates that certain cytokine levels such as IL-6 may be elevated (see Section 4.4). Elevated cytokines are a hallmark of CAR-T cell therapies often leading to CRS and ICANS which can be serious or fatal.

Consequently, the management of such toxicities have been extensively reviewed through the CAR-T Toxicity Management Workgroup [Lee et al, 2019]. To be fully prepared in the event that CRS is observed, this trial will follow the same guidelines as are used for CAR-T studies as outlined in Section 6.8.1 through Section 6.8.2.

All subjects will be monitored for potential AEs such as CRS. In addition, there will be a 1-week stagger between enrollment of the first subject in a cohort and the subsequent subjects. In addition, all patients will be asked to stay within one hour travel time to the clinic for at least 24 hours following their first ELI-002 dose.

6.8.1. Management of Cytokine Release Syndrome

Since ELI-002 activates T cells by a novel amphiphile mechanism whereby potent responses are generated, there is a theoretical risk for observation of T-cell-mediated toxicity.

To reduce the risk of CRS, subjects will not be given a repeat dose of ELI-002 if they are exhibiting T >38°C and/or IL-6 >500 pg/mL. In addition, if a subject exhibits IFNγ ≥1000 pg/mL, the Elicio medical monitor should be contacted for consultation.

In the event the ELI-002 dose is held, the subject should not receive any make up dose and remain on the original protocol schedule (ie, their next dose will be at the next protocol-specified dose if they meet the criteria for dosing and with approval from the medical monitor).

Cytokine release syndrome is caused by the release of inflammatory cytokines such as IL-6, IL-2, IFNγ, and TNFα, which causes a systemic inflammatory response similar to sepsis. The release of cytokines occurs when cells that are targeted by antibodies, immune effector cells (T and B-cells, Natural Killer cells, and monocytes/macrophages) are recruited to the tumor area and the subject's immune cells are activated. The importance of cytokine secretion of CAR T cells after exposure to tumor cells is closely related to their killing activity, and the degree of cytokine elevation is correlated to the bulk of residual disease at the time of adoptive T-cell infusion, ie, tumor burden, as well as with the number of detectable CAR T-cells after administration [Brentjens, 2012; Lee et al, 2015, Lee et al, 2019]. IL-6 levels in patients with low grade 0-3 CRS were a median of 100 pg/mL, whereas IL-6 was a median of 10,000 pg/mL [Teachey et al, 2016] in patients with high grade 4-5 CRS. Likewise, IFNγ was approximately 40 pg/mL in patients with low grade CRS, versus approximately 4,000 pg/mL in those with high grade CRS [Teachey et al, 2016] ; therefore, this protocol takes a conservative cut-point and mandates dose hold for ELI-002 if IL-6 >500 pg/mL is observed or contact with the medical monitor in the event that IFNγ > 1000 pg/mL is observed, making the assumption that although CRS guidelines address CAR-T safety management, ELI-002 may elicit sufficient T cells to merit a similar approach. The goal of CRS management is to prevent life-threatening complications while preserving the antitumor T cell immune response.

Clinical signs and symptoms of CRS listed in Table 17 should raise concern.

Table 17: Clinical Symptoms of Cytokine Release Syndrome

System	Symptom
**Constitutional**	Fever ± rigors, malaise, fatigue, anorexia, myalgias, arthralgias
**Biochemical**	Evidence of disseminated intravascular coagulation and MAS, elevation in liver function tests, D-dimers, LDH, CRP, uric acid and phosphorus
**Skin**	Rash
**Gastrointestinal**	Nausea, vomiting, diarrhea
**Respiratory**	Tachypnoea, hypoxaemia
**Cardiovascular**	Tachycardia, widened pulse pressure, hypotension, arrhythmias, increased cardiac output (early), potentially diminished cardiac output (late)
**Coagulation**	Disseminated intravascular coagulation, elevated D-dimer, hypofibrinogenaemia ± bleeding
**Renal**	Azotaemia
**Hepatic**	Transaminitis, hyperbilirubinaemia
**Neurological**	Headache, mental status changes, confusion, delirium, word-finding difficulty or frank aphasia, hallucinations, tremor, dysmetria, altered gait, seizures, cerebral edema

CRP=C-reactive protein; LDH=lactate dehydrogenase; MAS=macrophage activation syndrome

The Common Toxicity Criteria (ie, CTCAE) grading system was originally developed to capture a cytokine syndrome occurring during infusion therapy; therefore, it is inadequate to capture the delayed CRS that occurs after repeat dose ELI-002 administration. Appreciating the scale needed to be adapted for other therapeutics, to define mild, moderate, severe and life-threatening events, account for overlapping symptoms and guide treatment recommendations, and allow cross-trial comparison, an objective revised consensus CRS grading system was developed by the American Society for Transplantation and Cellular Therapy (ASTCT) as provided in Table 18 [Lee et al, 2019].

Table 18: Suggested Treatment by Cytokine Release Syndrome Grade

Grade	Treatment Requirement	Immunosuppressive Treatment
**Grade 1**	Symptoms are not life-threatening and require **symptomatic treatment only** (eg, fever, nausea, fatigue, headache, myalgias, malaise). Vigilant supportive care. Assess for infection, treat fever and neutropenia if present, monitor fluid balance	Acetaminophen and other antipyretics/analgesic supportive measures
**Grade 2**	Symptoms require and respond to **moderate intervention**:<br>• Oxygen requirement <40%<br>• Vigilant Supportive Care	Acetaminophen and other antipyretic/analgesic supportive measures. If multiple comorbidities or older age are present initiate IL-6 inhibition with tocilizumab ± corticosteroids
**Grade 3**	Symptoms require and respond to **aggressive intervention**:<br>• Oxygen requirement ≥40% or<br>• Hypotension requiring at least one vasopressor<br>• Vigilant Supportive Care	Initiate immunosuppressive therapy using tocilizumab and corticosteroids.
**Grade 4**	**Life-threatening symptoms**<br>• Requirement for ventilator support or<br>• Grade 4 organ toxicity (excluding transaminitis)	Initiate immunosuppressive therapy using tocilizumab and corticosteroids
**Grade 5**	**Death**	-

High-dose vasopressor doses shown in Table 19.
[Lee et al, 2019]

When applicable, use of high-dose vasopressor is recommended as described in Table 19.

Table 19: High-dose Vasopressors (All Doses are Required for ≥3 Hours)

Therapy	Dose
Norepinephrine monotherapy	≥20 µg/kg/min
Dopamine monotherapy	≥10 µg/kg/min
Phenylephrine monotherapy	≥200 µg/kg/min
Epinephrine monotherapy	≥10 µg/kg/min
If on vasopressin	Vasopressin + NE equivalent of ≥10 µg/kg/min[a]
If on combination vasopressors (not vasopressin)	Norepinephrine equivalent of ≥20 µg/kg/min[a]

[a] VASST Trial Vasopressor Equivalent Equation: Norepinephrine equivalent dose = [norepinephrine (µg/min)] + [dopamine (µg/kg/min) ÷ 2] + [epinephrine (µg/min)] + [phenylephrine (µg/min) ÷ 10]

6.8.2. Cytokine Release Syndrome Treatment Guidelines

In the event CRS is observed, vigilant supportive care, including empiric treatment of concurrent bacterial infections and maintenance of adequate hydration and blood pressure is required for every grade. Blood pressure instability will be evaluated in the context of blood pressures obtained at baseline and at Day 1 prior to the ELI-002 vaccine. Appropriate respiratory support will be provided as clinically indicated. Every attempt should be made to discern and treat potentially overlapping symptoms arising from infections, tumor lysis syndrome, or other

medical complications. Blood samples for cytokine and other analysis judged to be appropriate by the investigator will be obtained as soon as possible and daily until clinical concern for CRS has resolved.

The following clinical laboratory parameters will be followed in addition to expeditious local laboratory cytokine analysis:

- C-reactive protein (CRP)
- Complete blood count (CBC) with differential
- Blood chemistries
- Liver function tests
- Ferritin
- Coagulation parameters including prothrombin time, partial thromboplastin time, fibrinogen, D-dimer
- Blood cultures

Tocilizumab must be used as the first-line agent in the treatment of CRS. If the subject's condition does not improve or stabilize within 24 hours of the tocilizumab dose, administration of a second dose of tocilizumab and/or a second immunosuppressive agent (such as corticosteroids) should be considered.

- Tocilizumab is a recombinant humanized anti-human IL-6 receptor monoclonal antibody of the immunoglobulin (Ig) IgG1k subclass. It binds both the membrane bound and soluble forms for the IL-6 receptor, and thus blocks IL-6 mediated pro-inflammatory effects. Tocilizumab is approved for the treatment of CRS occurring in the setting of CAR-T cell therapy, rheumatoid arthritis, systemic juvenile idiopathic arthritis and polyarticular juvenile rheumatoid arthritis [ACTEMRA® USPI].
- Tocilizumab at a dose of 8mg/kg (max dose 800 mg; or at the level recommended by local standards) should be administered intravenously over 1-hour, with the option to repeat the dose if clinical improvement does not occur within 24 to 48 hours. There should be a minimum of 8 hours between consecutive doses of tocilizumab [Grupp et al, 2013; Lee et al, 2014; Maude et al, 2015].
- Corticosteroids: Corticosteroids may ablate or significantly impair T-cell function and may limit therapeutic benefit. However, they may be used as a secondary agent or in cases of severe or life-threatening CRS as front-line therapy or in conjunction with Tocilizumab.
- Methylprednisolone (1mg/kg every 12 hours) is recommended, but consideration should be given to using Dexamethasone (10 mg/dose every 6 hours) in subjects with neurologic symptoms who may benefit from more efficient penetration of the blood brain barrier [Bonifant et al, 2016; Maude et al, 2014; Neelapu et al, 2018].
- Additional agents: Siltuximab (anti-IL6 mAb) [Chen et al, 2016], Etanercept (soluble TNFα receptor antagonist), Dasatinib (tyrosine kinase inhibitor) [Mestermann et al,

2019], and Anakinra (IL-1R antagonist) [Giavridis et al, 2018] are investigational but may be considered for use in the context of severe CRS.

Subjects will be advised via the informed consent of the signs and symptoms of CRS. Subjects will be given a protocol-specific card with all necessary information for health care professionals to be aware that the subject is on a clinical trial and to contact the Primary Investigator (PI) and/or team. Subjects will be advised to seek immediate medical attention if such signs and symptoms occur.

Subjects with signs and symptoms of CRS will have blood collected as soon as possible and then at least daily for cytokine and other analysis judged to be appropriate by the investigator until clinical concern for CRS has resolved. Local laboratory cytokine and other appropriate analysis will be undertaken as expeditiously as possible.

6.8.3. ICANS Treatment Guidelines

Neurotoxicity (eg, encephalopathy, somnolence, aphasia; ICANS) has been observed with previous CAR T-cell therapies and since ELI-002 potently activates T cells there is a theoretical risk for ICANS.

Evaluation of any new onset ≥Grade 2 neurotoxicity should include a neurological examination, brain MRI, and examination of the cerebrospinal fluid (CSF) as clinically indicated.

Endotracheal intubation may be needed for airway protection in severe ICANS cases. Tocilizumab and corticosteroids should be used as outlined in Table 22, and anti-epileptic may be considered as clinically indicated. Medications with sedative properties should be avoided, if possible, in the setting of neurotoxicity. ICANS should be graded in accordance with the ASTCT consensus definitions:

The Immune Effector Cell-Associated Encephalopathy (ICE) scoring system is provided in Table 20, and the current ASTCT ICANS Consensus Grading for Adults is provided in Table 21.

Table 20: Encephalopathy Assessment Tool for Grading ICANS

	**ICE Scoring System**
Orientation	Orientation to year, month, city, hospital: 4points
Naming	Ability to name 3 objects (eg, point to clock, pen, button): 3 points
Following Commands	Ability to follow simple commands (eg, “Show me 2 fingers” or “Close your eyes and stick out your tongue”): 1 point
Writing	Ability to write a standard sentence (eg, “Our national bird is the bald eagle.”): 1 point
Attention	Ability to count backwards from100 by10: 1 point

ICANS=Immune effector cell-associated neurotoxicity syndrome; ICE=Immune Effector Cell-Associated Encephalopathy.
[Lee et al, 2019]

Table 21: ASTCT ICANS Consensus Grading for Adults

Neurotoxicity Domain	Grade 1	Grade 2	Grade 3	Grade 4
ICE Score[a]	7-9	3-6	0-2	0 (subject is unarousable and unable to perform ICE)
Depressed level of consciousness[b]	Awakens spontaneously	Awakens to voice	Awakens to tactile stimulus	Subject is unarousable or requires vigorous or repetitive tactile stimuli to arouse. Stupor or coma
Seizure	N/A	N/A	Any clinical seizure focal or generalized that resolves rapidly or nonconvulsive seizures on EEG that resolve with intervention	Life-threatening prolonged seizure (>5 min); or repetitive clinical or electrical seizures without return to baseline in between
Motor findings[c]	N/A	N/A	N/A	Deep focal motor weakness such as hemiparesis or paraparesis
Elevated intracranial pressure/ cerebral edema	N/A	N/A	Focal/local edema on neuroimaging[d]	Diffuse cerebral edema on neuroimaging; decerebrate or decorticate posturing; or cranial nerve VI palsy; or papilledema; or Cushing's triad

ASTCT=American Society for Transplantation and Cellular Therapy; CTCAE= Common Terminology Criteria for Adverse Events; ICANS=Immune effector cell-associated neurotoxicity syndrome; ICE=Immune Effector Cell-Associated Encephalopathy; N/A=not applicable

ICANS grade is determined by the most severe event (ICE score, level of consciousness, seizure, motor findings, raised intracranial pressure/cerebral edema) not attributable to any other cause; for example, a subject with an ICE score of 3 who has a generalized seizure is classified as grade 3 ICANS.

a: A subject with an ICE score of 0 may be classified as grade 3 ICANS if awake with global aphasia, but a subject with an ICE score of 0 may be classified as grade 4 ICANS if unarousable.

b: Depressed level of consciousness should be attributable to no other cause (eg, no sedating medication).

c: Tremors and myoclonus associated with immune effector cell therapies may be graded according to CTCAEv5.0, but they do not influence ICANS grading.

d: Intracranial hemorrhage with or without associated edema is not considered a neurotoxicity feature and is excluded from ICANS grading. It may be graded according to CTCAEv5.0.

[Lee et al, 2019]

Table 22: Neurotoxicity Suggested Treatment

Neurotoxicity<br>Grading assessment (CTCAE 5.0)	Evaluation	Treatment
<u>Grade 1 – Examples include</u>:<br>Somnolence-mild drowsiness or sleepiness<br>Confusion-mild disorientation<br>Encephalopathy-mild limiting of ADL<br>Dysphasia-not impairing ability to communicate	Neurological examination<br>Additional work up as clinically indicated	Vigilant supportive care
<u>Grade 2 – Examples include</u>:<br>Somnolence-moderate, limiting instrumental ADL<br>Confusion-moderate disorientation, limiting instrumental ADL<br>Encephalopathy-limiting instrumental ADL<br>Dysphasia-moderate impairing ability to communicate spontaneously	Consider EEG as clinically indicated<br>Should include brain MRI and evaluation of CSF in addition to neurological examination	Vigilant supportive care<br>Consider prophylactic anti-epileptic
<u>Grade 3 – Examples include</u>:<br>Somnolence-obtundation or stupor<br>Confusion-severe disorientation, limiting self-care ADL<br>Encephalopathy-limiting self- care ADL<br>Dysphasia-severe receptive or expressive characteristics, impairing ability to read, write or communicate intelligibly		Consider tocilizumab 4-8 mg/kg IV over 1 hour (not to exceed 800 mg)<br>Repeat tocilizumab if symptoms have not stabilized or improved within 12-24 hours<br>Consider corticosteroids (eg, dexamethasone 10 mg IV q6h) for worsening symptoms despite tocilizumab<br>Consider prophylactic anti-epileptic
<u>Grade 4 – Examples include</u>:<br>Life-threatening consequences<br>Urgent Intervention Indicated<br>Mechanical Ventilation		Corticosteroids (eg, dexamethasone 10 mg IV q6h)<br>Administer tocilizumab as per guidance for Grade 3 neurotoxicity if not previously administered<br>Consider prophylactic anti-epileptic

ADL=activities of daily life; CSF=cerebrospinal fluid; CTCAE= Common Terminology Criteria for Adverse Events; EEG=electroencephalogram; IV=intravenous; MRI=magnetic resonance imaging

6.8.4. Management of On-target Off-tumor Toxicity

Examples of on-target off-tumor toxicities have been observed in different immunotherapies where these events were not expected from pre-clinical data. It is difficult to anticipate all “autoimmune inflammation” that theoretically may be induced by an on-target off-tumor effect of ELI-002. For that reason, Grade 3 or higher autoimmune toxicity is considered a DLT (ie, fits non-hematologic ≥Grade 3 toxicity; see Section 6.7.3) Any observed treatment emergent autoimmune events must be followed closely. The exact diagnostic and the therapeutic approach to be followed will be left to the investigator’s discretion who can contact at any time the Sponsor Medical Monitor. The Sponsor may discuss any specific case with the SMC members to evaluate appropriate therapeutic action.

6.8.5. Management of Maculo-Papular or Acneiform Rashes, Injection Site Reactions

For routine injection site reactions manifested as maculo-papular or acneiform rashes, erythema, induration, pruritis and pain, topical application of cool compresses, antihistamine or corticosteroids may be used. If needed, systemic antihistamines and antipyretics may be added.

7. SELECTION AND WITHDRAWAL OF SUBJECTS

7.1. Subject Inclusion Criteria

Subjects must meet the following criteria on screening examination to be eligible to participate in the trial:

1. Male or female subjects aged ≥18 years
2. Histologically or cytologically confirmed diagnosis of solid tumor
3. Depending on the clinical site ability to access acceptable tumor sample for analysis: available tumor sample and EDTA blood sample for central lab WES biomarker analysis (preferable) or a blood sample for central lab plasma RAS ctDNA assay, which demonstrates at least 1 of the 2 mKRAS/NRAS alleles (eg, G12D or G12R) targeted by ELI-002 2P
4. High relapse risk, as evidenced by biomarker data with the last value obtained ≥21 days after surgery or last administration of adjuvant therapy, whichever comes last in the specific subject's treatment plan (values must not be attributable to a non-cancer condition, such as pancreatitis, peritonitis, postoperative leak/fistula, or biliary obstruction) as follows:
   a. ctDNA blood test positive, or
   b. successive rising values (≥1 week apart) in a serum tumor biomarker (such as CA19-9, CEA, and CA-125), or
   c. individual biomarker values CA19-9 ≥90 U/mL, or CEA ≥15 ng/mL, or CA-125 ≥35 U/mL
5. If subject has PDAC:
   a. Stage I, II, III or Stage IV oligometastatic (<3 lesions in one organ) disease per current American Joint Committee on Cancer (AJCC) staging criteria, with radiographic NED (no evidence of disease) status following surgery. Equivocal radiographic findings (eg, subcentimeter lesions, potential resolving soft tissue changes after surgery) are acceptable.
   b. Prior treatment with a standard of care chemotherapy or chemotherapy/chemoradiation (eg, FOLFIRINOX, gemcitabine/nab-paclitaxel, or 5-FU/capecitabine) according to NCCN pancreatic cancer guidelines and administered in neoadjuvant and/or adjuvant setting
   c. Successful surgical resection (focal use of intraoperative irreversible electroporation (eg, Nanoknife®) is permitted): Complete resection (R0) or with microscopic residual disease (R1)
6. If subject has CRC:
   a. High risk Stage II (T4N0), Stage III (T4N1-2/TanyN2), or Stage IV oligometastatic disease per current AJCC staging criteria, with radiographic NED status following surgery. Equivocal radiographic findings (eg, subcentimeter lesions, potential resolving soft tissue changes after surgery) are acceptable.

b. Prior treatment with standard of care chemotherapy (eg, FOLFOX, CAPEOX) according to NCCN colon cancer guidelines and administered in neoadjuvant and/or adjuvant setting
c. If the subject has rectal cancer, prior treatment with standard of care chemoradiation (eg, 5-FU or capecitabine- based) according to NCCN rectal cancer guidelines and administered in the neoadjuvant or adjuvant setting, or as total neoadjuvant therapy
d. For non-rectal subjects, successful surgical resection (focal use of intraoperative irreversible electroporation (eg, Nanoknife®) is permitted): Complete resection (R0) or with microscopic residual disease (R1)

7. If the subject has NSCLC Stage IB, II, or III disease per current AJCC staging criteria (8th edition) with receipt of stage-appropriate standard of care therapy per NCCN guidelines. The criteria below are examples of current common clinical scenarios based on stage. Since some agents are recently approved for specific clinical settings, alternative historical standard of care regimens will be allowed with approval of the medical monitor.

   a. Stage IB (T3-T4): complete surgical resection (R0). Prior systemic therapy is not required.
   b. Stage II:
      i. neoadjuvant SOC platinum-based chemotherapy according to NCCN guidelines with checkpoint inhibitor (if the tumor is ≥4 cm or node positive), followed by complete surgical resection (R0)
      ii. complete surgical resection (R0), followed by adjuvant chemotherapy with a SOC platinum doublet (eg, cisplatin/pemetrexed, cisplatin/gemcitabine, cisplatin/docetaxel, cisplatin/vinorelbine, or carboplatin/paclitaxel) and checkpoint inhibitor (if the tumor is >1% $PDL1^{+}$) according to NCCN guidelines
   c. Resectable Stage III:
      i. Prior standard of care neoadjuvant platinum-based chemotherapy:
         a) with or without checkpoint inhibitor (if the tumor is ≥4 cm or node positive), followed by complete surgical resection (R0)
         b) with or without radiation, followed by complete surgical resection (R0), followed by checkpoint inhibitor (if the tumor is >1% $PDL1^{+}$)
      ii. Complete surgical resection (R0) followed by SOC adjuvant platinum-based chemotherapy (with or without radiation for N2 disease) followed by a checkpoint inhibitor (if the tumor is >1% $PDL1^{+}$) according to NCCN guidelines
   d. Unresectable Stage III: concurrent standard of care chemotherapy and radiation followed by consolidation with checkpoint inhibitor regardless of PDL1 expression
   e. Equivocal radiographic findings (eg, subcentimeter lesions, potential resolving soft tissue changes after surgery) are acceptable.

8. If subject has ovarian cancer:

   a. Stage II, III or IV disease per current AJCC staging criteria, including ovarian, fallopian tube and primary peritoneal cancers or borderline tumors
   Primary debulking surgery with optimal cytoreduction (residual disease <1cm [R1] or removal of macroscopic disease [R0]) followed by six cycles of standard of care platinum-based adjuvant chemotherapy (eg, paclitaxel 175/carboplatin or pyrimidine-

based 5-FU/leucovorin/oxaliplatin or capecitabine/oxaliplatin) or neoadjuvant standard of care platinum-based chemotherapy with interval debulking surgery for subjects who were not candidates for primary debulking surgery due to disease unlikely to be optimally cytoreduced, followed by standard of care adjuvant platinum or pyrimidine based chemotherapy (eg, paclitaxel 175/carboplatin, or 5-FU/leucovorin/oxaliplatin or capecitabine/oxaliplatin). Equivocal radiographic findings (eg, potential resolving soft tissue changes after surgery) are acceptable.

9. If subject has CCA or gallbladder carcinoma:
    a. Localized and surgically resected intrahepatic, perihilar or distal CCA, or surgically resected gallbladder carcinoma (focal use of intraoperative irreversible electroporation (eg, Nanoknife®) is permitted) with complete resection (R0) or microscopic residual disease (R1). Equivocal radiographic findings (eg, subcentimeter lesions, potential resolving soft tissue changes after surgery) are acceptable.
    b. For subjects with CCA and R0 resection, observation or standard of care adjuvant treatment or chemoradiation per the list below; for CCA and R1 resection, standard of care adjuvant treatment or chemoradiation per the list below
    c. For subjects with gallbladder carcinoma (with either R0 or R1 resection), standard of care neoadjuvant, adjuvant or chemoradiation per the list below

    Neoadjuvant (gallbladder only) eg, 5-FU oxaliplatin, capecitabine oxaliplatin, gemcitabine capecitabine, gemcitabine cisplatin, 5-FU cisplatin, capecitabine cisplatin, gemcitabine cisplatin nab-paclitaxel, gemcitabine oxaliplatin, 5-FU, capecitabine, gemcitabine

    Adjuvant, eg, capecitabine, 5-FU oxaliplatin, capecitabine oxaliplatin, gemcitabine capecitabine, gemcitabine cisplatin, 5-FU cisplatin, capecitabine cisplatin, 5-FU, gemcitabine (gallbladder and intrahepatic CCA only)

    Chemoradiation, eg, XRT with 5-FU, XRT with capecitabine

10. Screening CT is negative for recurrent disease
11. Laboratory values must be:
    a. Absolute neutrophil count $\geq 1.5 \times 10^9$/L
    b. Platelets $\geq 100 \times 10^9$/L
    c. Hemoglobin ≥9 g/dL
    d. Total bilirubin ≤1.5 x ULN, unless subject has documented Gilbert's syndrome
    e. Aspartate aminotransferase (AST) or alanine aminotransferase (ALT) ≤2.5 x ULN
    f. Serum creatinine <1.5 mg/dL (or if serum creatinine is ≥1.5 mg/dL, the creatinine clearance calculated by Cockcroft-Gault formula must be ≥60 mL/min)
    g. Albumin ≥2.5 g/dL
    h. IL-6 < 500 pg/mL
12. Eastern Cooperative Oncology Group (ECOG) performance status of 0 or 1
13. Subjects have recovered from prior surgery, chemotherapy, or radiation without any ongoing medical/surgical issues (excluding resolved, low grade or chronic stable findings such as neuropathy, impaired hearing, or reproductive changes)

14. Women of childbearing potential and men with a female partner of childbearing potential must be willing to use effective methods of contraception during trial participation and for 30 days after the last dose of trial drug to prevent pregnancy as follows:

    a. Women: willing to use contraceptives with a failure rate <1% per year when used consistently and correctly, eg, combined oral contraceptives, barrier methods, approved contraceptive implant, long- term injectable contraception, or intrauterine device, sexual abstinence, or a vasectomized partner

       Definitions that meet the criteria to not be considered of childbearing potential:

       – Women >2 years postmenopausal (defined as 1 year or longer since last menstrual period) AND >55 years of age

       – Postmenopausal women (as defined above) and <55 years of age with a negative pregnancy test within 24 hours of first dose

       – Women who were surgically sterilized at least 3 months prior to enrollment

    b. Men: willing to use an acceptable method of birth control, eg, condom with spermicide. In addition, men must refrain from donating sperm.

15. Able to provide written (signed) informed consent to participate in the trial prior to any trial specific screening procedures

7.2. Subject Exclusion Criteria

Subjects must not meet any of the following criteria on screening examination to be eligible to participate in the trial:

1. Has received an anti-tumor therapy including investigational drug within 4 weeks prior to trial drug administration
2. Is currently receiving any agent with a known effect on the immune system, unless at dose levels that are not immunosuppressive (eg, inhaled corticosteroids at doses used for the treatment of asthma) (see Section 8.1.1)
3. Has any other serious illnesses or medical conditions such as, but not limited to:

    a. Any uncontrolled infection
    b. Uncontrolled cardiac failure classification III or IV (New York Heart Association)
    c. Myocardial infarction ≤6 months prior to enrollment
    d. Serious or uncontrolled cardiac arrhythmia, unstable angina
    e. History of or active seizure disorder
    f. History of or active auto-immune disease (excluding mild conditions, such as vitiligo, type 1 diabetes, psoriasis)
    g. History of or active interstitial lung disease/pneumonitis requiring treatment with systemic steroids
    h. Prior organ transplant

4. Baseline pulse oximetry <92% on room air at screening

5. Known history of positive tests for human immunodeficiency virus/acquired immune deficiency syndrome (HIV)/AIDS or hepatitis B
6. Hepatitis C subjects are excluded unless they have had a sustained virologic response to direct-acting antiviral therapy; such subjects must have an undetectable hepatitis C virus RNA at screening
7. Active severe acute respiratory syndrome (SARS)-CoV-2 (excluding subjects who have completed 2 weeks of quarantine and are considered clinically stable per investigator judgement); SARS-CoV-2 antibody test positivity is permitted.
8. Pregnant or lactating females
9. Women of childbearing potential who have a confirmed positive pregnancy test result at Baseline. If the urine test is positive or cannot be confirmed as negative, a serum pregnancy test will be required.
10. Had any other malignancies within the last 3 years (except for adequately treated carcinoma of the cervix, bladder, prostate, basal or squamous cell skin cancer), and in the investigator's judgement, there is no anticipation that the patient would need treatment for the other malignancy in the next 2 years, except for endocrine therapy for early-stage tumors.
11. Known malignant brain lesion(s). Subjects with previously treated brain metastases may be eligible provided they are stable (ie: without evidence of progression by imaging for at least 4 weeks prior to the first dose of ELI-002 7P, and any neurologic symptoms have returned to baseline), and there is no evidence of new or enlarging brain metastases.
12. Pancreatic neuroendocrine tumor or germline BRCA 1/2
13. If a subject has pancreatic cancer:
    a. Any tumor mutation where specific therapy is approved, and the subject is able to receive the approved therapy (except in cases where the subject cannot receive the approved therapy, such as due to a contraindication)
14. If subject has CRC:
    a. Prior immunotherapy as part of their adjuvant regimen
    b. Mismatch repair defective (MSI+)
    c. Any tumor mutation where specific therapy is approved, and the subject is able to receive the approved therapy
15. If subject has NSCLC:
    a. Any tumor mutation where specific therapy is approved, and the subject is able to receive the approved therapy
16. If subject has ovarian cancer, BRCA 1/2 or other hereditary cancer syndrome

7.3. Subject Withdrawal Criteria

A subject may voluntarily withdraw or be withdrawn from the trial at any time for reasons including, but not limited to, the following:

- The subject wishes to withdraw from further participation.
- The subject is significantly noncompliant with the protocol.
- Continuation in the trial would be detrimental to the subject's safety in the opinion of the investigator.

Any subject who wishes to withdraw from the trial may do so at any time without it affecting their medical care. The investigator may withdraw a subject from the trial at any time if it is felt to be in the best interest of the subject.

If a treated subject is discontinued prematurely from further treatment, the investigator must provide an explanation in the subject source records, as well as the eCRF and perform all End of Treatment evaluations according to Visit 15 in Table 14. These subjects should then enter the Follow-up Period, where procedures listed in Table 15 will be performed. In the event that a subject will not continue to be evaluated in the Follow-up Period, the End of Study visit (Visit 24) should be scheduled, and all procedures performed.

In the event that a subject does not return for the Follow-up evaluations, the investigator must make every effort to contact the subject to review all AEs and to determine the reason for dropping out (see Section 7.3.2).

In the event that a subject discontinues prematurely due to an AE or SAE assessed to be study drug-related, the event should be followed until it resolves (returned to normal or baseline values), stabilizes, or is judged by the investigator to be no longer clinically significant. Follow-up of an AE may also be continued at the request of the Sponsor Medical Monitor.

7.3.1. Subject Replacement

If a subject discontinues for any reason other than toxicity prior to completing 28 days of ELI-002 therapy, a replacement subject will be added to the cohort until 3 subjects per cohort complete 28 days of ELI-002 therapy.

7.3.2. Subjects Lost to Follow-up

If a subject is lost to follow-up, every attempt must be made to contact the subject and to determine the reason for dropping out. If the subject cannot be reached after three documented attempts over a reasonable amount of time, a certified letter must be sent to the subject asking them to contact the trial center for follow-up. Only after all above documented attempts will a subject be considered lost to follow-up.

8. TREATMENT OF SUBJECTS

8.1. Concomitant Medications

Subjects should receive medication appropriate to their health condition during the entire trial. All concomitant medications during the length of the trial, including screening, will be documented in the subject source record and in the appropriate electronic case report form (eCRF) page.

8.1.1. Prohibited Concomitant Medications

The following concomitant medications should not be used during study drug administration and are criteria for permanent stopping of the trial treatment administration for an individual subject

- Growth factors (except erythropoietin). The effect of growth factor administration on expansion and persistence of ELI-002 is unknown.
- Corticosteroids and other immunosuppressive agents except as needed for management of safety events. Systemic immunosuppressive agents should not be used while in the trial, except if needed for counteracting an AE, eg, cytokine release syndrome. However, the use of corticosteroids for anti-allergic reaction/symptoms, eg, pre/post tumor imaging (contrast product); topical and inhaled steroids are permitted.
- Cytotoxic agents and any other cancer therapy
- Investigational products/devices

8.2. Treatment Compliance

All subjects are expected to be fully complaint throughout the trial (see Section 6.7.2).

Subjects will receive SC injections of ELI-002 by qualified site staff who are under the supervision of the investigator or designee.

Treatment administration will be recorded on the eCRF.

8.3. Randomization and Blinding

ELI-002 is an open-label, Phase 1 trial with a single arm. Treatment will not be blinded and there will not be randomization as part of this study.

9. TRIAL DRUG MATERIALS AND MANAGEMENT

9.1. Trial Drug

ELI-002 is an investigational product consisting of 2 drug products:

1. A mixture of lipid-conjugated peptide-based antigens (amphiphilic peptides, referred to as 'Amph-Peptides'). During the study, the Amph-Peptide mixture (Amph-Peptides 2P) will be comprised of 1.4 mg of 2 Amph modified KRAS peptides, Amph-G12D, and Amph-G12R (0.7 mg/peptide). Elicio plans to investigate the use of an Amph-Peptide 7P drug product containing all 7 Amph-Peptides (G12D, G12R, G12V, G12A, G12C, G12S, G13D) in future clinical trials.
2. A lipid-conjugated immune-stimulatory oligonucleotide ('Amph-CpG-7909')

The ELI-002 admixture is prepared by combining the separately provided products in a diluent prior to dosing. The KRAS Amph-Peptides are provided in 5.0 mM, pH 4.5 acetate buffer in a 2-mL glass vial. The Amph-CpG-7909 is provided in water for injection (WFI) in a 2-mL glass vial. These 2 drug products will then be provided to the clinical pharmacy for combination with 10X PBS (phosphate buffered saline) on a dose-by-dose basis at the proper ratio for administration to the subject as a drug product admixture. For further details, refer to the Product Manual.

In the event of toxicity after immunization period doses, Amph-Peptides without Amph-CpG-7909 adjuvant may be administered during the booster period.

The Product Manual will provide instructions for the admixing procedure as well as storage instructions.

9.2. Trial Drug Packaging and Labeling

The ELI-002 study product will be provided in two separate kits comprised of 10 vials of Amph-CpG-7909and 10 vials of Amph-Peptides respectively. In addition, kits containing 10 vials of 10X PBS will be provided to sites. Each vial is for single use only and is labeled according to local regulatory requirements. ELI-002 is for subcutaneous injection.

9.3. Trial Drug Storage

The Amph-Peptides will contain a mixture of 2 KRAS Amph-Peptide drug substances. The Amph-Peptides are provided as a clear, colorless to slightly yellow, sterile frozen liquid. The recommended storage condition is -20ºC. For further details please refer to the Product Manual.

The Amph-CpG-7909 drug product will be a clear, colorless, sterile liquid fill for injection and the adjuvant will be produced by admixing with the Amph-Peptides drug product. The intended storage condition is -20°C. For further details please refer to the Product Manual.

9.4. Trial Drug Preparation

For details and instructions on preparation of the study drug, please see the ELI-002-001 Product Manual.

9.5. Administration and Dosage

Subjects will receive SC injections of ELI-002 during the Immunization Period at Visit 3 through Visit 8 (Table 12). Three months after the Immunization Period, subjects will receive SC injections of ELI-002 during the Booster Period at Visit 12 through Visit 15 (see Section 6.1.4, Table 14).

The doses evaluated in the cohorts are as shown in Table 23. All cohorts will enroll RAS mutated PDAC, CRC, NSCLC and OST (including ovarian, bile duct and gallbladder carcinoma).

Table 23: Phase 1 Cohorts and Doses

	Cohort	Tumor	Dose Level
**Dose Escalation**	Cohort 1	KRAS/NRAS mutated solid tumors	Amph-CpG-7909 0.1 mg with Amph-Peptides
	Cohort 2	KRAS/NRAS mutated solid tumors	Amph-CpG-7909 0.5 mg with Amph-Peptides
	Cohort 3	KRAS/NRAS mutated solid tumors	Amph-CpG-7909 2.5 mg with Amph-Peptides

KRAS=Kirsten rat sarcoma; NRAS=neuroblastoma ras viral oncogene homolog; OST=other solid tumors

9.6. Trial Drug Accountability

The investigator is responsible for trial drug accountability, reconciliation, and record maintenance. Drug accountability records will be maintained during the trial, including the amount of trial drug received from the Sponsor (or Sponsor's designee), the amount distributed to each subject, and the amount of unused drug destroyed at the site or returned to the Sponsor for destruction.

The trial center will maintain a log of all trial drug prepared and administered to subjects, as well as destruction of used/unused vials. Administration of the drug product admix (the test article) will occur during the Immunization Period and the Booster Period, according to the Schedule of Assessments (Table 12 and Table 14).

Trial center personnel should refer to the Product Manual for instructions regarding disposal of any trial drug or used trial drug packaging.

9.7. Trial Drug Handling and Disposal

All used and unused trial drug supplies must remain stored at the clinical site in a secure location and within appropriate temperature conditions up until receiving instructions for destruction. Disposal of trial drug will be conducted in compliance with all applicable laws and regulations.

10. TIMING OF TRIAL PROCEDURES

Home Health Visits: Based on assessment of risk, and to ensure subject safety and minimize risks to trial integrity, off-site study visits can be performed in the event a study participant cannot attend an on-site visit during the COVID-19 crisis [Food and Drug Guidance, 2020]. The procedures that cannot be performed during a home health visit include:

- CT/MRI scans
- First ELI-002 dose administration (Visit 3, Day 57); this must occur on-site as per Section 10.2.
- Leukapheresis (Visit 2, Visit 9)

The informed consent form will describe the availability of home health visits if applicable at the clinical site.

The subject will be invited to continue future study visits on-site per protocol, as soon as local and federal Covid-19 regulations allow.

10.1. Screening and Observation Period

Visit 1 (Day 10), subjects will provide written informed consent before any trial-related procedures are performed.

- Once a subject has consented among the first procedures will be:
    - A retrospective tumor tissue sample from the prior surgery will be requisitioned for WES. Tumor DNA will be sequenced to guide ctDNA testing in subsequent blood samples and baseline tumor gene expression will be assessed (Table 11).
    - In parallel, a 6-mL ethylenediaminetetraacetic acid (EDTA) whole blood tube will be collected and shipped at the same time as the tissue sample. The somatic DNA from the EDTA tube will serve as a control to ensure only tumor-specific DNA is included for subsequent ctDNA testing.

At **Visit 2 (Day 26 ±5 days)**, the following visit procedures will include:

- Administer the EORTC QLQ-C30 and the tumor type-specific PRO (see Section 11.3).
- Collect a urine sample for a pregnancy test for women of childbearing potential.
- Record demographic information (including sex, age, race and ethnicity).
- Record height and weight.
- Record medical and medication history, including cancer history/prior treatments and current medications.
- Review available eligibility criteria. While mKRAS/mNRAS and ctDNA results may not yet be available, the expectation is that these will be reviewed, and full eligibility determined, prior to Visit 3.

- Perform a physical examination which will be based on institution standard of care(see Section 12.1.3).
- Record vital signs (blood pressure, pulse rate, body temperature, and respiratory rate). Measurements will be obtained after 3 minutes in the sitting position. No other measurements or procedures should be performed during this 3-minute period.
- Perform a 12-lead ECG (to be reviewed by a cardiologist or clinical investigator according to institutional guidelines and practice).
- Perform an ECOG Scale of Performance Status. To be eligible, ECOG performance status must be 0 to 1.
- Perform a CT with contrast within 90 days of first ELI-002 dose, following resection or after last administration of adjuvant treatment, whichever is last in the specific subject treatment plan. For subjects with IV contrast allergy/intolerance, MRI may be performed. The same modality should be used for all imaging throughout the trial.
- Collect a blood sample for clinical laboratory assessments, including the cytokine panel (local laboratory; see Section 11.1 and Section 12.1.6).
- Collect a sample appropriate for the available test method for active Covid-19 infection (local laboratory).
- Collect a blood sample for ctDNA test ≥21 days post-surgery or after last administration of adjuvant treatment, whichever is last in the specific subject treatment plan (to be performed at a central laboratory).
- Collect blood sample(s) (local laboratory, see Inclusion Criteria #4 in Section 7.1). Biomarker values must be obtained ≥21 days post-surgery or after the last administration of adjuvant therapy, whichever comes last in the specific subject's treatment plan.
- Collect a leukapheresis for immunogenicity assessment (central laboratory). The leukapheresis must be collected once eligibility has been confirmed and prior to the first dose of ELI-002.
- Record any pre-treatment SAEs that have occurred.

10.1.1. Rescreening

Subjects are allowed to rescreen for the ELI-002-001 trial. No screening procedures, other than what may need to be repeated due to screen failure, will need to be repeated as long as the screening CT/MRI scan was performed ≤90 days from the time of the anticipated first dose of trial drug. If the CT/MRI scan was performed >90 days from the anticipated first dose, then it will need to be repeated and reviewed, to ensure that the patient is negative for recurrent disease, per the protocol inclusion criteria.

Other screening evaluations can be repeated as deemed appropriate and necessary by the investigator.

10.2. Immunization Period

During the Immunization Period, subjects will receive 4 weekly SC injections (Weeks 1 to 4) followed by 2 injections at Week 6 and Week 8 (Table 12). If the subject has fever ≥38.0°C and/or elevated IL-6 ≥500 pg/mL, proceed with the visit but omit study treatment administration; if fever abates and cytokines diminish to <500 pg/mL, the subject may proceed with the next scheduled dose after approval from the medical monitor). In addition, if a subject exhibits IFNγ ≥1000 pg/mL, the Elicio medical monitor should be contacted for consultation.

All laboratory samples should be taken prior to dosing on any study visit that has dosing.

At **Visit 3 (Baseline, Day 57) prior to trial drug administration,** the following visit procedures will include:

- Administer the EORTC QLQ-C30 and the tumor type-specific PRO (see Section 11.3).
- Record any change in medical history since the previous visit. Subjects must have recovered from surgery without any ongoing medical/surgical issues.
- Record any change in cancer history since the previous visit.
- Perform a physical examination which will be based on institution standard of care (see Section 12.1.3). In addition, a neurological examination for baseline assessment of ICANS/ICE will be performed.
- Record vital signs (blood pressure, pulse rate, body temperature, and respiratory rate).
- Record weight.
- Perform a 12-lead ECG (to be reviewed by a cardiologist or clinical investigator according to institutional guidelines and practice).
- Confirm ECOG Scale of Performance Status is 0 or 1.
- Record any change in concomitant medications since the previous visit.
- Collect a blood sample for clinical laboratory assessments, including the cytokine panel (local laboratory; see Section 11.1 and Section 12.1.6). Note: cytokine testing should be performed within 7 days prior to dosing.
- Collect a blood sample for ctDNA (to be performed at a central laboratory).
- Collect a blood sample for high resolution HLA typing (central laboratory).
- Record any pre-treatment SAEs that have occurred since the previous visit.

Dose 1: The first dose must be administered at the clinical site. Subjects must be observed by study staff for at least 1-hour postdose for observation and must stay within an hour of the clinic for ≥24 hours in order to monitor for potential AEs such as CRS.

After dose administration the following visit procedures will occur:

- Record vital signs (blood pressure, pulse rate, body temperature, and respiratory rate). Measurements will be obtained after 3 minutes in the sitting position. No other measurements or procedures were performed during this 3-minute period.
- Subjects will be provided with the reactogenicity diary to complete for 7-days following the dose. Subjects will be instructed how to complete the diary and to return the completed form at their next visit.

At **Visit 4 (Day 64)**, the following visit procedures will include:

- Administer the EORTC QLQ-C30 and the tumor type-specific PRO (see Section 11.3).
- Perform a physical examination, which will be based on institution standard of care (see Section 12.1.3).
- Record vital signs (blood pressure, pulse rate, body temperature, and respiratory rate). Measurements will be obtained after 3 minutes in the sitting position. No other measurements or procedures were performed during this 3-minute period.
- Record weight.
- Confirm ECOG Scale of Performance Status.
- Record any change in concomitant medications since the previous visit.
- All laboratory samples should be taken prior to dosing on any study visit that has dosing.
  - Collect a blood sample for clinical laboratory assessments, including the cytokine panel (local laboratory; see Section 11.1 and Section 12.1.6). Note: cytokine testing should be performed and reviewed within 7 days prior to dosing.
- Record any AEs since last visit. Check for signs of toxicity. Including fever, and laboratory evidence of elevated cytokines. Hold the ELI-002 dose if Grade ≥2 CRS is present, or if elevated cytokine IL-6 is present. If elevated IFNγ is present, the Elicio medical monitor should be contacted for consultation(Section 6.8.1).
- **Dose 2:** Trial drug administration and subjects must be observed by study staff for at least 1-hour postdose for observation.
- Subjects will be provided with the reactogenicity diary to complete for 7-days following the dose. Subjects will be instructed how to complete the diary and to return the completed form at their next visit.

At **Visit 5 (Day 71)**, the following visit procedures will include:

- Administer the EORTC QLQ-C30 and the tumor type-specific PRO (see Section 11.3).
- Perform a physical examination, which will be based on institution standard of care (see Section 12.1.3).

- Record vital signs (blood pressure, pulse rate, body temperature, and respiratory rate). Measurements will be obtained after 3 minutes in the sitting position. No other measurements or procedures were performed during this 3-minute period.
- Record weight.
- Confirm ECOG Scale of Performance Status.
- Record any change in concomitant medications since the previous visit.
- All laboratory samples should be taken prior to dosing on any study visit that has dosing.
  - Collect a blood sample for clinical laboratory assessments, including the cytokine panel (local laboratory; see Section 11.1 and Section 12.1.6). Note: cytokine testing should be performed and reviewed within 7 days prior to dosing.
  - Collect a whole blood sample for immunogenicity testing (central laboratory).
- Record any AEs since last visit. Check for signs of toxicity, including fever and laboratory evidence of elevated cytokines. Hold the ELI-002 dose if Grade ≥2 CRS is present, or if elevated cytokine IL-6 is present. If elevated IFNγ is present, the Elicio medical monitor should be contacted for consultation (Section 6.8.1).
- **Dose 3**: Trial drug administration and subjects must be observed for safety for at least 1-hour postdose.
- Subjects will be provided with the reactogenicity diary to complete for 7-days following the dose. Subjects will be instructed how to complete the diary and to return the completed form at their next visit.

At **Visit 6 (Day 78)**, the following visit procedures will include:

- Administer the EORTC QLQ-C30 and the tumor type-specific PRO (see Section 11.3).
- Perform a physical examination, which will be based on institution standard of care (see Section 12.1.3).
- Record vital signs (blood pressure, pulse rate, body temperature, and respiratory rate). Measurements will be obtained after 3 minutes in the sitting position. No other measurements or procedures were performed during this 3-minute period.
- Record weight.
- Confirm ECOG Scale of Performance Status.
- Record any change in concomitant medications since the previous visit.
- All laboratory samples should be taken prior to dosing on any study visit that has dosing.
  - Collect a blood sample for clinical laboratory assessments, including the cytokine panel (local laboratory; see Section 11.1 and Section 12.1.6). Note: cytokine testing should be performed and reviewed within 7 days prior to dosing.

  - Collect a blood sample for ctDNA.

- Record any AEs since last visit. Check for signs of toxicity, including fever and laboratory evidence of elevated cytokines. Hold the ELI-002 dose if Grade ≥2 CRS is present, or if elevated cytokine IL-6 is present. If elevated IFNγ is present, the Elicio medical monitor should be contacted for consultation (Section 6.8.1).
- **Dose 4**: Trial drug administration and subjects must be observed for safety for at least 1-hour postdose.
- Subjects will be provided with the reactogenicity diary to complete for 7-days following the dose. Subjects will be instructed how to complete the diary and to return the completed form at their next visit.

At **Visit 7 (Day 92)**, the following visit procedures will include:

- Administer the EORTC QLQ-C30 and the tumor type-specific PRO (see Section 11.3).
- Perform a physical examination, which will be based on institution standard of care (see Section 12.1.3).
- Record vital signs (blood pressure, pulse rate, body temperature, and respiratory rate). Measurements will be obtained after 3 minutes in the sitting position. No other measurements or procedures were performed during this 3-minute period.
- Record weight.
- Confirm ECOG Scale of Performance Status.
- Record any change in concomitant medications since the previous visit.
- All laboratory samples should be taken prior to dosing on any study visit that has dosing.
  - Collect a blood sample for clinical laboratory assessments, including the cytokine panel (local laboratory; see Section 11.1 and Section 12.1.6). Note: cytokine testing should be performed and reviewed within 7 days prior to dosing.
  - Collect a whole blood sample for immunogenicity testing (central laboratory).
- Record any AEs since last visit. Check for signs of toxicity, including fever and laboratory evidence of elevated cytokines. Hold the ELI-002 dose if Grade ≥2 CRS is present, or if elevated cytokine IL-6 is present. If elevated IFNγ is present, the Elicio medical monitor should be contacted for consultation (Section 6.8.1).
- **Dose 5**: Trial drug administration and subjects must be observed for safety for at least 1-hour post dose.
- Subjects will be provided with the reactogenicity diary to complete for 7-days following the dose. Subjects will be instructed how to complete the diary and to return the completed form at their next visit.

At **Visit 8 (Day 106)**, the following visit procedures will include:

- Administer the EORTC QLQ-C30 and the tumor type-specific PRO (see Section 11.3).
- Perform a physical examination, which will be based on institution standard of care (see Section 12.1.3).
- Record vital signs (blood pressure, pulse rate, body temperature, and respiratory rate). Measurements will be obtained after 3 minutes in the sitting position. No other measurements or procedures were performed during this 3-minute period.
- Record weight.
- Confirm ECOG Scale of Performance Status.
- Record any change in concomitant medications since the previous visit.
- All laboratory samples should be taken prior to dosing on any study visit that has dosing.
  - Collect a blood sample for clinical laboratory assessments, including the cytokine panel (local laboratory; see Section 11.1 and Section 12.1.6). Note: cytokine testing should be performed and reviewed within 7 days prior to dosing.
- Record any AEs since last visit. Check for signs of toxicity, including fever and laboratory evidence of elevated cytokines. Hold the ELI-002 dose if Grade ≥2 CRS is present, or if elevated cytokine IL-6 is present. If elevated IFNγ is present, the Elicio medical monitor should be contacted for consultation (Section 6.8.1).
- **Dose 6**: Trial drug administration and subjects must be observed for safety for at least 1-hour postdose.
- Subjects will be provided with the reactogenicity diary to complete for 7-days following the dose. Subjects will be instructed how to complete the diary and to return the completed form at their next visit.

10.3. No Dosing 3-Month Period

After the Immunization Period, subjects will be observed during a No Dosing 3-Month Period (Table 13).

At **Visit 9 (Day 120),** the following visit procedures will include:

- Administer the EORTC QLQ-C30 and the tumor type-specific PRO (see Section 11.3).
- Perform a physical examination, which will be based on institution standard of care (see Section 12.1.3).
- Record vital signs (blood pressure, pulse rate, body temperature, and respiratory rate). Measurements will be obtained after 3 minutes in the sitting position. No other measurements or procedures were performed during this 3-minute period.

- Record weight.
- Record any change in concomitant medications since the previous visit.
- Collect a blood sample for clinical laboratory assessments (local laboratory; see Section 12.1.6).
- Collect leukapheresis for immunogenicity assessments (central laboratory). This procedure may be performed -7 days to +3 days from Visit 9.
- Record any AEs since last visit.

At **Visit 10 (Day 148) and Visit 11 (Day 176)**, the following visit procedures will include:

- Administer the EORTC QLQ-C30 and the tumor type-specific PRO (see Section 11.3).
- Perform a physical examination, which will be based on institution standard of care (see Section 12.1.3).
- Record vital signs (blood pressure, pulse rate, body temperature, and respiratory rate). Measurements will be obtained after 3 minutes in the sitting position. No other measurements or procedures were performed during this 3-minute period.
- Record weight.
- Record any change in concomitant medications since the previous visit.
- At **Visit 10 (Day 148 [±14 days])** only perform a CT with contrast. For subjects with IV contrast allergy/intolerance, MRI may be performed. The same modality as the screening imaging assessment should be used throughout the trial.
- Collect a blood sample for clinical laboratory assessments (local laboratory; see Section 12.1.6).
  - At Visit 11 (Day 176) only – collect a blood sample for the cytokine panel (local laboratory; see Section 11.1). Note: cytokine testing should be performed and reviewed within 7 days prior to dosing.
- At **Visit 10 (Day 148 [±14 days])** only collect a serum sample for CA-125/CA 19-9/CEA for appropriate tumor type (local laboratory).
- At **Visit 11 (Day 176)** only collect a blood sample for ctDNA (to be performed at a central laboratory):
- At **Visit 11 (Day 176)** collect a whole blood sample for immunogenicity testing (central laboratory)
- Record any AEs since last visit.

10.4. Booster Period

During the Booster Period, eligible subjects will receive booster vaccinations, consisting of 4 weekly injections (Table 14). If the subject has fever ≥38.0°C and/or elevated IL-6 ≥500 pg/mL, proceed with the visit but omit study treatment administration; if fever abates and

cytokines diminish to <500 pg/mL, the subject may proceed with the next scheduled dose after approval from the medical monitor). In addition, if a subject exhibits IFNγ ≥1000 pg/mL, the Elicio medical monitor should be contacted for consultation.

At **Visit 12 (Day 196)**, the following visit procedures will include:

- Administer the EORTC QLQ-C30 and the tumor type-specific PRO (see Section 11.3).
- Perform a physical examination, which will be based on institution standard of care (see Section 12.1.3).
- Record vital signs (blood pressure, pulse rate, body temperature, and respiratory rate). Measurements will be obtained after 3 minutes in the sitting position. No other measurements or procedures were performed during this 3-minute period.
- Record weight.
- Confirm ECOG Scale of Performance Status.
- Record any change in concomitant medications since the previous visit.
- All laboratory samples should be taken prior to dosing on any study visit that has dosing.
  - Collect a blood sample for clinical laboratory assessments, including the cytokine panel (local laboratory; see Section 11.1 and Section 12.1.6). Note: cytokine testing should be performed and reviewed within 7 days prior to dosing.
  - Collect a blood sample for ctDNA (to be performed at a central laboratory).
- Record any AEs since last visit. Check for signs of toxicity, including fever and laboratory evidence of elevated cytokines. Hold the ELI-002 dose if Grade ≥2 CRS is present, or if elevated cytokine IL-6 is present. If elevated IFNγ is present, the Elicio medical monitor should be contacted for consultation (Section 6.8.1).
- **Dose 7**: Trial drug administration and subjects must be observed for safety for at least 1-hour postdose.
- Subjects will be provided with the reactogenicity diary to complete for 7-days following the dose. Subjects will be instructed how to complete the diary and to return the completed form at their next visit.

At **Visit 13 (Day 203)**, the following visit procedures will include:

- Administer the EORTC QLQ-C30 and the tumor type-specific PRO (see Section 11.3).
- Perform a physical examination, which will be based on institution standard of care (see Section 12.1.3).
- Record vital signs (blood pressure, pulse rate, body temperature, and respiratory rate). Measurements will be obtained after 3 minutes in the sitting position. No other measurements or procedures were performed during this 3-minute period.

- Record weight.
- Confirm ECOG Scale of Performance Status.
- Record any change in concomitant medications since the previous visit.
- All laboratory samples should be taken prior to dosing on any study visit that has dosing.
  - Collect a blood sample for clinical laboratory assessments, including the cytokine panel (local laboratory; see Section 11.1 and Section 12.1.6). Note: cytokine testing should be performed and reviewed within 7 days prior to dosing.
- Record any AEs since last visit. Check for signs of toxicity, including fever and laboratory evidence of elevated cytokines. Hold the ELI-002 dose if Grade ≥2 CRS is present, or if elevated cytokine IL-6 is present. If elevated IFNγ is present, the Elicio medical monitor should be contacted for consultation (Section 6.8.1).
- **Dose 8**: Trial drug administration and subjects must be observed for safety for at least 1-hour postdose.
- Subjects will be provided with the reactogenicity diary to complete for 7-days following the dose. Subjects will be instructed how to complete the diary and to return the completed form at their next visit.

At **Visit 14 (Day 210)**, the following visit procedures will include:

- Administer the EORTC QLQ-C30 and the tumor type-specific PRO (see Section 11.3).
- Perform a physical examination, which will be based on institution standard of care (see Section 12.1.3).
- Record vital signs (blood pressure, pulse rate, body temperature, and respiratory rate). Measurements will be obtained after 3 minutes in the sitting position. No other measurements or procedures were performed during this 3-minute period.
- Record weight.
- Confirm ECOG Scale of Performance Status.
- Record any change in concomitant medications since the previous visit.
- All laboratory samples should be taken prior to dosing on any study visit that has dosing.
  - Collect a blood sample for clinical laboratory assessments, including the cytokine panel (local laboratory; see Section 11.1 and Section 12.1.6). Note: cytokine testing should be performed and reviewed within 7 days prior to dosing.
  - Collect a whole blood sample for immunogenicity testing (central laboratory).
- Record any AEs since last visit. Check for signs of toxicity, including fever and laboratory evidence of elevated cytokines. Hold the ELI-002 dose if Grade ≥2 CRS is

present, or if elevated cytokine IL-6 is present. If elevated IFNγ is present, the Elicio medical monitor should be contacted for consultation (Section 6.8.1).

- **Dose 9**: Trial drug administration and subjects must be observed for safety for at least 1-hour postdose.
- Subjects will be provided with the reactogenicity diary to complete for 7-days following the dose. Subjects will be instructed how to complete the diary and to return the completed form at their next visit.

At **Visit 15/ End of Treatment (Day 217)**, this visit is to be performed when the subject completes the treatment period or if they discontinue treatment early and prior to entering the follow-up period of the study. The visit procedures will include:

- Administer the EORTC QLQ-C30 and the tumor type-specific PRO (see Section 11.3).
- Perform a physical examination, which will be based on institution standard of care (see Section 12.1.3).
- Record vital signs (blood pressure, pulse rate, body temperature, and respiratory rate). Measurements will be obtained after 3 minutes in the sitting position. No other measurements or procedures were performed during this 3-minute period.
- Record weight.
- Confirm ECOG Scale of Performance Status.
- Record any change in concomitant medications since the previous visit.
- All laboratory samples should be taken prior to dosing on any study visit that has dosing.
  - Collect a blood sample for clinical laboratory assessments, including the cytokine panel (local laboratory; see Section 11.1 and Section 12.1.6). Note: cytokine testing should be performed and reviewed within 7 days prior to dosing.
- Record any AEs since last visit. Check for signs of toxicity, including fever and laboratory evidence of elevated cytokines. Hold the ELI-002 dose if Grade ≥2 CRS is present, or if elevated cytokine IL-6 is present. If elevated IFNγ is present, the Elicio medical monitor should be contacted for consultation (Section 6.8.1).
- **Dose 10**: Trial drug administration and subjects must be observed for safety for at least 1-hour postdose.
- Subjects will be provided with the reactogenicity diary to complete for 7-days following the dose. Subjects will be instructed how to complete the diary and to return the completed form at their next visit.

10.5. Follow-up Period

A Follow-up Period will provide safety and efficacy data (Table 15 and Table 16).

At every visit during the Follow-up Period, **Visit 16 (Day 231), Visit 17 (Day 259), Visit 18 (Day 315), Visit 19 (Day 399), Visit 20 (Day 483), Visit 21 (Day 567), Visit 22 (Day 651), Visit 23 (Day 735), Visit 24 (Day 819), Visit 25 (Day 903), Visit 26 (Day 987), Visit 27 (Day 1071) and Visit 28/End of Study (Day 1127)**, the following visit procedures will include:

- Confirm ECOG Scale of Performance Status.
- Collect a blood sample for ctDNA test (central laboratory).
- Record any AEs that occur within 30 days of last dose and follow any such AEs until they resolve or return to baseline level.

In addition, the following assessments will be conducted at these Follow-up visits:

At **Visit 16 (Day 231),** the following visit procedures will include

- Collect a blood sample for clinical laboratory assessments, including the cytokine panel (local laboratory; see Section 11.1 and Section 12.1.6).
- Collect a whole blood sample for immunogenicity testing (central laboratory).

At **Visit 16 (Day 231)**, **Visit 17 (Day 259)**, **Visit 18 (Day 315)**, **Visit 19 (Day 399)**, **Visit 20 (Day 483)**, **Visit 21 (Day 567)**, **Visit 22 (Day 651)**, **Visit 23 (Day 735)**, and **Visit 24 (Day 819)** the following visit procedures will include

- Administer the EORTC QLQ-C30 and the tumor type-specific PRO (see Section 11.3).
- Record vital signs (blood pressure, pulse rate, body temperature, and respiratory rate). Measurements will be obtained after 3 minutes in the sitting position. No other measurements or procedures were performed during this 3-minute period.
- Record weight.

At **Visit 17 (Day 259)**, **Visit 19 (Day 399)**, **Visit 20 (Day 483)**, **Visit 21 (Day 567)**, **Visit 22 (Day 651)**, **Visit 23 (Day 735)**, **Visit 24 (Day 819)**, **Visit 25 (Day 903)**, **Visit 26 (Day 987)**, **Visit 27 (Day 1071)**, and **Visit 28/End of Study (Day 1127)**, the following visit procedures will include:

- Perform a CT (±14 days) with contrast. For subjects with IV contrast allergy/intolerance, MRI may be performed. The same modality as the screening imaging assessment should be used throughout the trial.
- Collect a serum sample for CA-125/CA 19-9/CEA for appropriate tumor type (local laboratory).

At **Visit 20 (Day 483),** the following visit procedures will include:

- Perform a physical examination, which will be based on institution standard of care (see Section 12.1.3).

At **Visit 22 (Day 651),** the following visit procedures will include:

- Collect a whole blood sample for immunogenicity testing (central laboratory).

The **Visit 28/End of Study** visit will be scheduled for subjects who complete the study or for any subject that discontinues from the study early.

11. CLINICAL ACTIVITY, IMMUNE ASSESSMENTS, AND SUBJECT REPORTED OUTCOMES

Clinical activity, immune assessments, and PROs will be collected according to the Schedule of Assessments (Table 11, Table 12, Table 13, Table 14, Table 15, and Table 16).

Median RFS and 1-year RFS rate will be assessed through CT imaging with contrast as assessed by the site investigators using iRECIST criteria [Seymour et al, 2017]; subjects will be followed for OS. Unscheduled CT or MRI imaging may be performed at any time, if the investigator determines that there are clinical signs of disease progression.

In addition, biomarker response rate will be based on the percentage of subjects achieving biomarker reduction or clearance, as measured by the absence or decrease of ctDNA from baseline and the duration of ctDNA clearance and/or reduction, or if ctDNA was not detectable at baseline, serum tumor biomarker (such as carbohydrate antigen [CA]19-9, carcinoembryonic antigen [CEA], and CA-125) reduction and clearance compared to baseline will be recorded. T cell responses in PBMCs will be assessed by immune assays such as IFNγ Fluorospot, ICS, and/or dextramers.

Immune responses will be correlated to clinical efficacy parameters.

11.1. Immunogenicity

Patients will have leukapheresis and blood samples drawn to evaluate the immunogenicity of ELI-002. T cell responses in PBMCs will be assessed by immune assays such as IFNγ Fluorospot, ICS, and/or dextramers. Serum cytokine, CA-125, CEA and CA 19-9 levels will also be examined.

HLA Typing: High resolution HLA typing will be used to identify certain individual variations in the immune system. Cohorts will be enrolled regardless of the HLA subtype.

Serum Cytokines: Concentrations of cytokines, IL-2, IFNγ, IL-6, IL-10, and TNFα, will be measured using multiplex technology in serum samples (local laboratory).

Cellular immune response: The cellular immune response to ELI-002 will be monitored by examining T cell responses using immune assays such as Fluorospot and ICS. The Fluorospot assay enumerates the release of IFNγ in human PBMCs following KRAS peptide stimulation. The ICS assay is a flow cytometry-based assay that can detect cytokine production by CD4+ and CD8+ T cells to the KRAS peptides. For both assays, PBMCs will be stimulated individually with mKRAS peptide pools to determine the frequency of KRAS-specific T cells per patient and timepoint. Dextramers are fluorescently labeled HLA multimers that can detect antigen-specific T cells. In subjects with HLA alleles for which dextramer(s) are available, the frequency of KRAS-specific T cells can be monitored in PBMCs before and after vaccination.

Full details of the leukapheresis or phlebotomy procedure to obtain PBMCs will be provided in the Central Laboratory Manual.

Standard of Care Biopsies: Tumor tissue specimens will be collected post-dosing from biopsies performed as standard of care per iRECIST criteria (ie; following observation of new lesions on radiographic imaging and investigator assessment that tissue can be safely obtained). If there is sufficient sample collected, the pathology laboratory should perform in situ gene

expression and/or IHC staining to examine tumor-infiltrating T cells and the tumor microenvironment.

11.2. Tumor Tissue Collection at Surgery

Once a subject has consented, and depending on the clinical site location, the trial coordinator will requisition a tumor tissue sample that was collected at surgery. Details for tumor tissue collection are provided in the Central Laboratory Manual).

11.3. Patient Reported Outcomes

Patient reported outcomes will be assessed by EORTC QLQ-C30 and QLQ-PAN26, QLQ-CR29, QLQ-OV28, QLQ-BIL21 or QLQ-LC13 and will be administered to subjects according to the Schedule of Assessments and prior to other visit procedures (Table 11, Table 12, Table 13, Table 14, Table 15, and Table 16). All the PROs have been fully validated by EORTC, except QLQ-PAN26, which has completed phase III of validation and is on-track for full validation in 2021. Verification of validation can be obtained through EORTC, and the list of questionnaires and the phase of validation can be found at the following site: https://qol.eortc.org/questionnaires/.

11.3.1. EORTC QLQ-C30

The European Organization for Research and Treatment of Cancer quality of life questionnaire (EORTC QLQ-C30) is a tool for assessing the quality of life (QoL) of cancer subjects participating in clinical trials. The EORTC QLQ-C30 incorporates 9 multi-item scales: 5 functional scales (Physical, Role, Cognitive, Emotional and Social Functioning); 3 symptom scales (Fatigue, Pain and Nausea/Vomiting); and a Global Health Status/QoL scale. Six single item scales are also included (Dyspnoea, Insomnia, Appetite Loss, Constipation, Diarrhoea and Financial Difficulties). The psychometric properties of the questionnaire have been determined to be valid, reliable, and sensitive [Aaronson et al, 1993; Osoba et al, 1994; Kaasa et al, 1995].

11.3.2. Tumor type-specific PROs

Tumor type-specific PROs are intended to supplement the EORTC QLQ-C30 with questions appropriate for the symptoms experienced by subjects with each tumor type. Please refer to the EORTC website for the PRO validation documentation. Tumor type-specific PROs will include:

- QLQ-PAN26: pancreatic cancer
- QLQ-BIL-21: biliary cancer
- QLQ-CR-29: colorectal cancer
- QLQ-LC-13: lung cancer
- QLQ-OV-28: ovarian cancer

11.4. ctDNA Levels

Levels of ctDNA (mean tumor molecules/mL) will be assessed using a ctDNA assay (central laboratory) in anticoagulated peripheral whole blood at indicated timepoints post vaccination. The ctDNA fold change from baseline will be calculated at each timepoint.

If a subject is MRD^- by ctDNA at screening, sites may continue to monitor ctDNA levels per their standard of care testing and rescreen if the subject becomes MRD^+ by ctDNA analysis. It is recommended that the time between ctDNA screening tests be ≥90 days.

12. ASSESSMENT OF SAFETY

12.1. Safety Parameters

12.1.1. Demographics and Medical History

Demographic variables will include age, gender, and race/ethnicity. Medical and medication history, including cancer history/prior treatments, current medications (prescription, herbal and dietary supplements, over-the-counter medications, and topical treatments), smoking history (for NSCLC subjects only), and diseases will be assessed at Screening and recorded in the source documents and the eCRF.

Weight and height will be collected during the Screening Period and weight will collected throughout the trial according to the Schedule of Assessments (Table 11, Table 12, Table 13, Table 14, Table 15, and Table 16).

12.1.2. Vital Signs

Vital signs will be collected throughout the trial according to the Schedule of Assessments (Table 11, Table 12, Table 13, Table 14, Table 15, and Table 16) and will include blood pressure, pulse rate, body temperature, and respiratory rate. Measurements will be obtained after 3 minutes in the sitting position. No other measurements or procedures were performed during this 3-minute period.

12.1.3. Physical Examinations

Full or targeted PEs will be performed at screening and at specified times during the trial (Table 11, Table 12, Table 13, Table 14, Table 15, and Table 16) based on standard of care.

12.1.4. Electrocardiogram

A resting 12-lead ECG will be performed according to the Schedule of Assessments (Table 11 and Table 12) and reviewed by a cardiologist or clinical investigator, per institutional guidelines and practice.

12.1.5. Eastern Cooperative Oncology Group

The ECOG Scale of Performance Status describes a subject's level of functioning in terms of their ability to care for themselves, daily activity, and physical ability (walking, working, etc.).

Eligibility for this trial requires a subject's ECOG performance status to be 0 to 1 (see Table 24).

The ECOG assessment will be performed according to the Schedule of Assessments (Table 11, Table 12, Table 13, Table 14, Table 15, and Table 16).

12.1.6. Laboratory Assessments

Laboratory tests will be performed according to the Schedule of Assessments (Table 11, Table 12, Table 13, Table 14, and Table 15). A central clinical laboratory will be utilized for biomarkers, but local clinical laboratories will be used for safety assessments.

The results of clinical laboratory tests conducted during the trial will be assessed by the investigator to determine each subject's appropriateness for trial participation.

For the remainder of the trial, the investigator will review laboratory values that are outside the normal reference ranges to determine whether the values are clinically significant. Clinically significant changes will be followed until the parameter returns to Baseline or until the investigator determines that follow-up is no longer medically necessary. Clinically significant values will be documented as Adverse Events as described in Section 12.2.1.1. Unscheduled re-tests may be performed as needed through the Follow-up Visit.

12.1.6.1. Hematology

The following hematology parameters will be assessed:

- White blood cell count
- Absolute/% Neutrophils
- Absolute/% Lymphocytes
- Absolute/% Monocytes
- Absolute/% Eosinophils
- Red blood cell count
- Hematocrit
- Hemoglobin
- Platelet Count
- Absolute/% Basophils

12.1.6.2. Coagulation

The following coagulation parameters will be assessed:

- Prothrombin time (PT)
- Partial Thromboplastin time (PTT)
- International Normalized Ratio (INR)
- Fibrinogen
- D-dimer

12.1.6.3. Blood Chemistry

The following blood chemistry parameters will be assessed:

- Sodium
- Potassium
- Chloride
- Creatinine
- Calcium
- Phosphate
- Glucose
- Alkaline phosphatase
- Lactate dehydrogenase
- Blood urea nitrogen (BUN)
- BUN/creatinine ratio
- Bilirubin (total)
- Protein (total)
- Albumin
- Uric acid
- Aspartate transaminase (AST)

- Bicarbonate
- LDH
- Magnesium
- Alanine transaminase (ALT)
- Creatine kinase
- Ferritin
- C-reactive protein (CRP)

12.1.6.4. Serum Cytokines

The following serum cytokines will be assessed:

- IL-2
- IFNγ
- IL-6
- IL-10
- TNFα

12.1.6.5. Viral Laboratory Testing at Screening

The following viral parameters will be assessed at Screening only:

- Hepatitis C RNA
- COVID-19 active infection

12.1.6.6. Pregnancy Screen

A urine pregnancy test will be performed according to the Schedule of Assessments (Table 11) for women of childbearing potential. Postmenopausal women must have been amenorrhoeic for at least 12 months to be considered of non-childbearing potential. Childbearing status for all women will be recorded in the source documents and in the eCRF.

A positive pregnancy test will exclude the subject from participation in the trial. Any clinically abnormal or inconclusive test will be repeated.

12.2. Adverse and Serious Adverse Events

12.2.1. Definition of Adverse Events

12.2.1.1. Adverse Event

An AE is the development of an undesirable medical condition or the deterioration of a pre-existing medical condition following or during exposure to a pharmaceutical product, whether or not considered study-drug related. An AE can be any unfavorable and unintended sign, symptom of disease temporally associated with the use of study drug, without any judgement about causality (see current ICH guidelines).

In this study, investigators should report AEs based on CTCAE v5.0 criteria, or ASTCT consensus criteria in the event of either CRS or immune effector cell associated neurotoxicity

syndrome (ICANS). Treatment-emergent AEs (TEAEs) are defined as events not present prior to the initiation of study drug or any event already present that worsens in either intensity of frequency following exposure to study treatment. AEs must be recorded in the eCRF and on forms as provided by Elicio.

An AE does not include the following:

- Medical or surgical procedures (eg, surgery, endoscopy, dental extraction, transfusion); the condition that necessitates the procedure is the AE
- Any pre-existing disease or condition, or laboratory or ECG abnormality, which is present prior to the start of the study medication and that does not worsen
- Laboratory or ECG abnormalities without clinical significance (ie, those which do not require medical intervention or that do not result in discontinuation or interruption of the study drug)
- Situations where an adverse medical event has not occurred (ie, hospitalization for elective surgery, social admission)
- Overdose of study medication or any concomitant medication without any signs or symptoms, unless the subject is hospitalized for observation

The time period for collecting and documenting AEs for each subject begins from the time the subject is administered the first dose of study drug through end of treatment (EOT) or early discontinuation of treatment.

12.2.1.2. Serious Adverse Event

An SAE is an AE occurring during any trial phase (ie, screening, treatment, or follow-up), and at any dose of the investigational product, that fulfils one or more of the following:

- Results in death
- It is immediately life-threatening
- It requires inpatient hospitalization or prolongation of existing hospitalization
- It results in persistent or significant disability or incapacity
- Results in a congenital abnormality or birth defect
- It is an important medical event that may jeopardize the subject or may require medical intervention to prevent one of the outcomes listed above.

The time period for collecting and documenting SAEs for each subject begins from the time the subject provides informed consent through the end of study (EOS) or early discontinuation from the study. For this study, the reporting of CRS and/or ICANS/Neurotoxicity events will follow the SAE timelines and reporting requirements.

12.2.1.3. Reactogenicity to the Vaccine

Injection site reactions, skin rash, and pruritis will not be considered a DLT or safety event and should be recorded in the patient diary and eCRF for reactogenicity. (See Section 6.7.3 for DLT

Guidelines, Section 6.8.5 for toxicity management, and Section 12.2.1.2 for SAE definition.) Severe skin rashes such as Steven-Johnson Syndrome or toxic epidermal necrolysis (TEN) will be considered a DLT and should be reported on the eCRF as safety events including SAE as appropriate. Reactogenicity monitoring will include subject's reactogenicity to the trial drug (vaccine). This will be captured at several time points as follows:

- 1-hour post ELI-002 dose administration (subjects will be observed in the clinic for at least 1-hour after each dose)
- 1-week post ELI-002 dose administration (subjects will record daily observations in diary)
- Any unresolved reactogenicity event should be followed until resolution.

12.2.1.4. Cytokine Release Syndrome

The definition, management, and grading of CRS are provided in Section 6.8.

12.3. Relationship to Trial Drug

An investigator who is qualified in medicine must make the determination of relationship to the investigational product for each AE (Unrelated, Possibly Related, or Related). The investigator should decide whether, in his or her medical judgement, there is a reasonable possibility that the event may have been caused by the investigational product.

Unrelated: The AE is definitely not associated with the study drug administration and is judged related to causes other than the study drug.

Possibly Related (Suspected Adverse Reaction [SAR]): A causal relationship between treatment with the study drug and the AE is at least a reasonable possibility, ie, the relationship cannot be ruled out. This implies a lesser degree of certainty about causality than a related AE. Additional evidence to suggest a SAR includes:

- Occurrence of uncommon AEs that are known to be strongly associated with drug exposure (eg, anaphylaxis, angioedema, blood dyscrasias, rhabdomyolysis, hepatic injury, Stevens-Johnson Syndrome)
- Occurrence of AEs that are uncommon in the study population, but not commonly associated with drug exposure (eg, intussusception in healthy infants)

Related (Adverse Reaction): A causal relationship between the study drug administration and the AE is definite.

If the relationship between the AE/SAE and the investigational product is determined to be "possible" the event will be considered related to the investigational product for the purposes of expedited regulatory reporting.

12.4. Severity of Adverse Events

The CTCAE Version 5 displays Grades 1 through 5 with unique clinical descriptions of severity for each AE based on this general guideline:

- **Grade 1** Mild; asymptomatic or mild symptoms; clinical or diagnostic observations only; intervention not indicated
- **Grade 2** Moderate; minimal, local or noninvasive intervention indicated; limiting age appropriate instrumental activities of daily living (such as preparing meals, shopping for groceries or clothes, using the telephone, managing money, etc.)
- **Grade 3** Severe or medically significant but not immediately life-threatening; hospitalization or prolongation of hospitalization indicated; disabling; limiting self-care activities of daily living (such as bathing, dressing and undressing, feeding self, using the toilet, taking medications, and not bedridden)
- **Grade 4** Life-threatening consequences; urgent intervention indicated
- **Grade 5** Death related to AE.

For CRS/ICANs, AEs will be graded according to the ASTCT criteria (Table 18 and Table 21).

12.5. Recording Adverse Events

Adverse events spontaneously reported by the subject and/or in response to an open question from the trial personnel or revealed by observation will be recorded during the trial at the investigational trial center. Treatment emergent AEs (TEAEs) are defined as events not present prior to the initiation of study drug or any event already present that worsens in either intensity of frequency following exposure to study treatment. AEs must be recorded in the eCRF and on forms as provided by Elicio. The AE term should be reported in standard medical terminology when possible. For each AE, the investigator will evaluate and report the onset (date and time), resolution (date and time), severity, causality, action taken, outcome, and whether or not it caused the subject to discontinue the trial.

Severity will be assessed according to CTCAE or ASTCT as appropriate for the event. For AEs not described in the consensus criteria, the following scale will be used:

- Mild (awareness of sign or symptom, but easily tolerated. Concomitant prescription medications are not ordinarily needed for relief of symptoms)
- Moderate (discomfort sufficient to cause interference with normal activities. Concomitant prescription medications/treatment(s) may be needed for relief of symptoms)
- Severe (incapacitating, with inability to perform normal activities. Requires interruption or discontinuation of study medication. Systemic drug therapy or other treatment is needed)

Reactogenicity will be assessed according to the toxicity grading scale for local reactions for preventive vaccines (see Section 12.2.1.3).

It is important to distinguish between serious and severe AEs. Severity is a measure of intensity whereas seriousness is defined by the criteria under Section 12.2.1.2. An AE of severe intensity may not be considered serious.

12.6. Pregnancy

Should a pregnancy occur within 30 days of the last ELI-002 dose in a female subject, or in the partner of a male subject, it must be reported on Elicio's paper pregnancy form. Neither pregnancy itself nor an induced elective abortion to terminate a pregnancy for non-medical reasons is considered an AE. However, an induced therapeutic abortion to terminate a pregnancy due to complications or other medical reasons will be recorded as an SAE. The underlying medical reason for this procedure should be recorded as the AE term. Pregnancy outcomes considered to be SAEs are:

- Spontaneous abortion (miscarriage)
- Stillbirth
- Induced therapeutic abortion to terminate pregnancy due to complications or other medical reasons
- Neonatal death; this includes all neonatal deaths that occur within 1 month of the expected birthdate (ie, either before or after the expected birth date)
- Infant death occurring 1 month after birth, if the investigator assesses the death as possibly related to in utero exposure to study treatment
- Congenital anomaly (in live birth or aborted fetus)

If the subject has signed the pregnancy informed consent, the outcome of all pregnancies (spontaneous miscarriage, elective termination, normal birth or congenital abnormality) must be followed up and documented even if the subject was discontinued from the trial.

12.7. Reporting Adverse Events

All AEs and SAEs will be reported on the appropriate eCRF pages, which will also be the reporting mechanism to Elicio safety/pharmacovigilance. All AEs (related and unrelated) will be recorded from first dose administration until 30 days after the end of study treatment administration. All SAEs (related and unrelated) will be recorded from the signing of the ICF until 30 days after the end of study treatment administration. All SAEs must be reported to Elicio or their designee within 1 business day of the first awareness of the event. The investigator must verify the accuracy of the information recorded on the SAE pages with the corresponding source documents.

Additional follow-up information, if required or available, should all be sent to Elicio or its designee within one business day of receipt and this should be completed on a follow-up SAE form, as well as documented with the original SAE source information and kept with the appropriate section of the subject source records.

Elicio is responsible for notifying the relevant regulatory authorities of certain events. It is the PI's responsibility to notify the Institutional Review Board (IRB) of all SAEs that occur at his or

her trial center in accordance with local IRB requirements. Investigators will also be notified of all unexpected, serious, drug-related events (Suspected Unexpected Serious Adverse Reaction [SUSAR] Reports) that occur during the clinical trial. Each trial center is responsible for notifying its IRB of these additional SAEs in accordance with local requirements. If the clinical site submits to a central IRB, SUSAR reports will be submitted by Elicio or its designee.

12.8. Extension Protocol

Subjects who remain in the trial until the end of the required Follow-up Period may be eligible to enroll in a separate Extension protocol to continue to receive further mKRAS/mNRAS lymph node immunotherapy to maintain effective levels of antigen-specific T-cells.

13. STATISTICS

ELI-002-001 is a multi-center Phase 1 trial to evaluate the safety and efficacy of ELI-002 in subjects with mKRAS/mNRAS solid tumors who have MRD defined as a positive ctDNA blood test or serum tumor biomarkers persisting ≥21 days post-surgery or after last administration of adjuvant therapy. The investigational product ELI-002 consists of up to 2 amphiphile-modified KRAS peptides plus an amphiphile-modified CpG adjuvant.

Each cohort will be evaluated separately; however, combined analysis of the RP2D and higher dose cohorts may be carried out to improve statistical precision via larger sample size.

13.1. Sample Size

Approximately 18 subjects are planned to enroll: 3 to 6 subjects in each of 3 cohorts. The sample size is not based on power calculations, but rather on clinical judgement and the expectation that the objectives of this trial will be met with approximately 18 subjects enrolled in the 3+3 design.

13.2. General Statistical Considerations and Definitions

13.2.1. General Statistical Methods

As a general approach, continuous variables will be summarized using mean, standard deviation, median, minimum value, and maximum value. Categorical variables will be summarized using frequency counts and percentages. Time to event data will be summarized using the Kaplan-Meier method. Where appropriate, CIs around point estimates will be presented, and estimates of the median and other quantiles, as well as individual time points (for time to event data) will be produced.

All statistical hypothesis testing within (for change from baseline) and between dose cohorts will be conducted at the 1-sided 0.05 level of significance. Nominal p-values may be computed for secondary or exploratory efficacy analyses as a descriptive measure of strength of results rather than for formal tests of hypotheses. Emphasis will be on RP2D, highest dose, and combined RP2D and all higher doses for change from baseline and comparison to lowest dose. No formal tests of hypotheses are planned for safety analyses.

The following methods will be used in this trial for descriptive summaries and hypothesis testing unless otherwise indicated:

- **Mean change from baseline.** Compared to zero change and compared between dose levels using t-tests from repeated measures as the analysis model and/or via non-parametric tests based on ranks, as appropriate.
- **Median time to event for a single group (non-comparative).** The Kaplan-Meier method will be used to estimate median time to event; 2-sided CIs will use the method of Brookmeyer and Crowley; p-values will not be computed.
- **Hazard ratio of a time to event endpoint comparing two groups.** A stratified Cox proportional hazards model using Efron's method for ties will be used to estimate the HR and 2-sided CI; p-values will be computed using the log rank test.

- **Time to event analysis at a fixed time point.** The Kaplan-Meier method will be used to estimate the survival (or event-free) probability; 2-sided Cis for a single survival probability or for a difference of two survival probabilities will use the complementary log-log method; p-values will be computed using the complementary log-log test statistic.
- **Binary endpoint for a single group (non-comparative)**. The binomial parameter (probability of success/failure) will be estimated by the sample proportion. 2-sided CIs will use the method of Clopper and Pearson; p-values will not be computed.
- **Comparison of binary endpoint between two groups**. Difference of sample proportions will be used to estimate the difference in binomial parameters. 2-sided CIs will use Newcombe's score method without continuity correction; p-values will be computed using stratified Cochran-Mantel-Haenszel test.

All descriptive statistical analyses will be performed using SAS statistical software (Version 9.3 or later), unless otherwise noted. Medical History and AEs will be coding using MedDRA.

No adjustment for multiple testing will be made.

13.2.2. Analysis Population

The following populations will be used for data analyses and presentations.

13.2.2.1. Full Analysis Set (FAS)

The Full Analysis Set (FAS) will include all subjects who received any amount of ELI-002 vaccine. This population will be used for summaries of subject demographics, and baseline characteristics. Additional exploratory analyses may be performed for the subjects treated at the MTD and doses lower than the MTD at which biomarker (ctDNA, or if ctDNA was not detected at baseline, serum tumor biomarker) clearance/reduction is observed.

13.2.2.2. Safety Analysis Set

The FAS will be used for the safety evaluation (AEs, clinical laboratory assessments, vital signs, ECGs, ECOG, PEs). No data will be imputed for the safety analysis set; only observed data will be summarized.

13.2.2.3. Disease Response Evaluable Set

All subjects who received any amount of ELI-002 vaccine who have positive MRD at baseline, and either:

1. Have at least 1 available post-baseline MRD assessment, or
2. Have experienced an RFS event (recurrence or death).

This population will be used for supportive analysis of biomarker clearance/reduction and duration of biomarker clearance/reduction.

13.2.2.4. Per Protocol Set

The Per Protocol Analysis Set will include all FAS subjects who have no major protocol violations. The exclusion list for this set will be identified prior to database lock.

13.2.2.5. PD/Biomarker Evaluable Set

All Phase 1 subjects who receive any amount of trial treatment, with the additional requirement that relevant blood sampling data at the appropriate assessment time point(s) must be available for inclusion in the analysis for the corresponding parameter.

13.2.3. Missing Data

There will be no substitutions made to accommodate missing data points unless specifically indicated otherwise. All data recorded on the CRF will be included in data listings that will accompany the clinical trial report.

13.3. Statistical Analyses

13.3.1. Demographic and Background Characteristics

Demographic and other baseline data, including but not limited to age, sex, race, ethnicity, height, weight, baseline ECOG Performance Status, primary diagnosis, disease stage at diagnosis and screening, and prior therapies (including systemic therapies, radiation, and surgeries, etc.) will be listed individually by subject and summarized by trial phase and treatment group using descriptive statistics (continuous data) or contingency tables (categorical data). The FAS will be used.

13.3.2. Immunogenicity Analysis

A positive immune response for each KRAS/NRAS peptide will be determined, for example, using a non-parametric distribution-free resampling method for the Fluorospot assay. For all immunogenicity assays, cellular immune responses to ELI-002 will be summarized for each patient by magnitude of response and fold-change from baseline for each timepoint tested. Immune responses will be summarized for each tumor type and dose level cohort.

Statistical tests such as Wilcoxon rank-sum test will be used to compare magnitude of response or changes before and after vaccination when applicable. The p-values associated with these tests will not be used for hypothesis testing.

13.3.3. Efficacy Analysis

13.3.3.1. Relapse-free survival

Relapse-free survival will be censored on the date of the last evaluable CT scan on trial for subjects who do not have recurrence and who do not die while on trial. Subjects without any post baseline CT scans will have their RFS time censored on the date of randomization. Subjects who withdraw from the trial (including withdrawal of consent and lost to follow-up) will be censored at the last of the last CT scan. For disease recurrence documented by CT between scheduled evaluations, the actual date of the scan will be used. For death or recurrence after no more than

1 missed evaluation, the date of the event will be the date of recurrence or death, whichever occurs first. For death or recurrence after more than 1 missed evaluation, RFS will be censored at the last evaluable disease assessment.

RFS (in months) is calculated as:

- [(date of recurrence/death – date of randomization) + 1)]/30.4375

13.3.3.2. Analysis of Biomarker Response

Response will be defined as any subject with a reduction or clearance of biomarker (ctDNA or serum tumor biomarker) compared with baseline [Tie et al, 2019]. Subjects who do not attain a response for any reason (eg, missing data due to withdrawal) will be counted as non-responders. The subset of subjects with biomarker clearance will also be summarized.

13.3.3.3. Analysis of Duration of Biomarker Response

Duration of biomarker response (ie, reduction or clearance) will be reported for subjects who meet the biomarker reduction or clearance definitions.

- Duration of biomarker reduction is calculated from the date of the first biomarker blood test with a reduction compared to baseline, until the earliest date of any of the following events: (1) subsequent increase in biomarker; (2) subsequent disease recurrence; or (3) death due to any cause.
- Duration of biomarker clearance is calculated from the date of the first negative ctDNA test or the date when the serum tumor biomarker is ≤ULN to the earliest date of any of the following events: (1) subsequent positive ctDNA test or subsequent serum tumor biomarker >ULN; (2) subsequent disease recurrence; or (3) death due to any cause.

If a subject has not experienced any of the events for reduction or clearance, duration of response will be censored on the date of the last biomarker assessment.

Response duration will be summarized for each cohort. Assessment of biomarker response duration is considered supportive of the primary biomarker response analysis and is not considered part of the fixed sequence testing procedure to control overall type 1 error.

13.3.3.4. Analysis of 1-Year RFS

Exploratory analysis of 1-year RFS will be reported.

13.3.3.5. Analysis of OS

Exploratory analysis of OS will be reported.

OS is calculated from the day of first ELI-002 administration to death. For subjects who are alive or lost to follow-up, OS is censored at the day the subject is last known alive as the last recorded date known alive. OS (in months) is defined as:

- [(date of death or last known alive – date of first ELI-002 dose) + 1)]/30.4375

13.3.4. Safety Analysis

Summary statistics (counts, percentage, mean, standard deviation, etc.) will be provided for the safety endpoints as appropriate.

Treatment-emergent adverse events (TEAEs) will be listed and tabulated by system organ class, preferred term, maximum CTCAE grade, relationship to trial treatment, outcome, and action taken with trial treatment. TEAEs will be tabulated by treatment regimen and overall and reviewed for potential significance and clinical importance. Adverse events qualifying as serious will be separately listed, along with any dose interruptions and reductions.

Significant PE findings and vital signs will be listed and summarized by treatment regimen. Clinical laboratory results will be presented in a listing including change from baseline and selected parameters will be summarized in shift tables. ECG derived data will be explored graphically and by summary statistics.

13.3.5. Pharmacokinetics

No pharmacokinetics analyses will be performed.

13.3.6. Pharmacodynamics

The pharmacodynamic response to ELI-002 will be assessed by evaluation of immunogenic responses to the vaccine.

14. DATA INTEGRITY AND QUALITY ASSURANCE

14.1. Data Management and Case Report Forms

Elicio and/or its designee will be responsible for the processing and quality control of the data. Data management will be performed as described in the sponsor's and/or designee's standard operating procedures (SOPs) for clinical studies and the study Data Management Plan. Adverse events will be coded using MedDRA and medications will be coded using the World Health Organization Drug Dictionary (WHO Drug).

The handling of data, including data quality control, will comply with regulatory guidelines (ie, current ICH E6) and the sponsor and/or designee SOPs.

The eCRF will be provided by the sponsor or its designee and should be completed in accordance with applicable study guidelines and within the timeframe specified. Each eCRF should be filled out completely by the investigator or designee as stated on the Delegation of Authority Log. All data entry in the eCRF must have corresponding source documentation located in the subject study records.

All data will be stored in a validated database that is 21 CFR Part 11 compliant. The principal investigator is responsible for ensuring the timely completion of eCRFs. The principal investigator (or sub-investigator) must review, sign and date the eCRFs at the time of study completion to attest to the accuracy and completeness of the study data at the clinical site.

14.2. Source Documentation

Source documents are defined as documentation of the observations and activities of a clinical study. Source documents may include, but are not limited to, study progress notes, email correspondence, subject QLQs (or PROs), subject diaries, laboratory data and pathology and/or imaging reports. All source documents for this study will be maintained by the investigator and made available to the sponsor, the sponsor designee and regulatory authorities. The signed informed consent document and statement of consent will be maintained in the subject study records, and a copy of the informed consent will be provided to the subject.

14.3. Study Drug Accountability

At all times, a record of the study drug inventory and study drug accountability must be maintained. Inventory and accountability records must be readily available to the study monitor, the sponsor, and any regulatory authority. The investigator, in conjunction with the site pharmacy or study drug preparation lab, is responsible for ensuring accountability of all used and unused study drug.

At the end of the trial, and following study monitor verification of drug accountability, Elicio or its designee will provide instruction for the destruction of unused study drug supplies remaining at the clinical site. During the conduct of the study, used vials may be destroyed on-site if Elicio has approved the destruction policy and only after the study monitor has verified drug accountability. If study drug destruction by the clinical site is approved by Elicio or its designee, the investigator must ensure that all study drug is destroyed in compliance with institutional

policy, applicable local regulations, as well as 21 CFR 312.59 and current ICH E6 guidelines pertaining to drug destruction. All destruction at the clinical site must be adequately documented.

14.4. Laboratory Assessments

Local laboratories will be responsible to analyze safety laboratory tests for this study. All local laboratory results will be captured on eCRFs. Central labs will be used for the analysis of pharmacodynamic and immunology assays, as well as specialty biomarkers.

14.5. Trial Monitoring

During the trial, Elicio and/or its designee will perform periodic monitoring visits to ensure that the protocol and ICH/GCP are being followed in the conduct of the study. Study monitoring will be performed in accordance with ICH/GCP, the sponsor and/or its designee's SOPs, the study protocol and study plans, as well as applicable local regulations. The monitor will maintain current personal knowledge of the study through observation, review of study records and source documentation, as well as discussion on the conduct of the study with the investigator and site staff. The monitor will review all study source documents to ensure that the eCRFs are completed and accurate.

The monitor, Elicio representatives and regulatory authorities will be given direct access to all study related documentation (such as medical charts/records, laboratory reports, diagnostic reports, study drug logs), study staff training documentation and institutional SOPs to verify study conduct. His verification may continue following completion of the study. It is important that the investigator and study staff are available during the monitoring visit and that sufficient time is devoted to items requiring follow-up or resolution.

14.6. Protocol Deviations

A deviation from the protocol is a departure from the procedures and/or processes approved by the sponsor and the IRB and agreed to by the principal investigator.

Protocol deviations will be reported to the sponsor or its designee as they occur or are discovered and must be documented as appropriate in the study source record. In addition, protocol deviations will be reported to the IRB per the individual IRB guidelines. The monitor will document deviations discovered throughout the course of monitoring visits and review of the eCRFs. These deviations, and any necessary corrective actions, will be reviewed and discussed with the sponsor and the investigators as applicable.

14.7. Audits and Inspections

During and/or after the completion of the study, authorized representatives of Elicio, a regulatory authority, or an IRB may visit the trial center to perform audits or inspections, including source data verification. The purpose of an audit or inspection, which is independent from routine monitoring, is to systematically and independently evaluate all trial-related activities and documents to determine whether these activities were conducted, according to the protocol, GCP and current ICH guidelines, and applicable regulatory requirements.

The investigators accept that by endorsing the protocol signature page that the sponsor, IRB or regulatory authorities may conduct an inspection to verify compliance to the protocol and GCP/ICH guidelines. Representatives of the sponsor, IRB or regulatory authority will be permitted direct access to source documents. It is important that the investigator and study staff are available during the inspection or audit and that sufficient time is given to addressing any questions or items that require resolution.

If a regulatory authority or IRB notifies the clinical site of an inspection related to the study, the investigator will immediately notify the sponsor. The investigator agrees to cooperate with Elicio or its designees, when possible, to be present at the inspection. The investigator also agrees to promptly provide the sponsor with copies of inspection findings. Before submitting a response to regulatory authorities, the investigator will give Elicio and/or its designees the opportunity to review the response and provide feedback.

14.8. Institutional Review Board and Other Committees

14.8.1. Institutional Review Board

Federal regulations and ICH guidelines require that IRB approval be obtained prior to participation of human subjects in research studies. Prior to the study start, the protocol, IB, informed consent form (ICF) and any other relevant documents must be approved by an IRB. IRB approvals, and all materials approved by the IRB for this trial must be maintained by the investigator and made available for inspection. Copies of all IRB approvals should be forwarded to Elicio or its designee.

The investigator is responsible for informing the IRB of any amendment to the protocol in accordance with local requirements. In addition, the IRB must approve all advertising used to recruit subjects for the trial. The protocol must be re-approved by the IRB upon receipt of amendments and annually, as local regulations require.

The investigator is also responsible for providing the IRB with reports of any SUSAR (suspected unexpected serious adverse reaction) from any other site or trial conducted with ELI-002. Elicio or its designee will provide this information to each participating site.

Progress reports and notifications of SUSARs will be provided to the IRB according to local regulations and guidelines.

If the clinical site submits to a central IRB, Elicio or its designee will submit the protocol, amendments, and any required safety notifications on the site’s behalf to the central IRB.

14.8.2. Safety Monitoring Committees

The SMC will meet at least once per cohort during the study. The SMC will consist of Elicio clinical representatives and the recruiting site investigators and will have the authority to assess and recommend any dosing changes, including dose escalation to the next cohort, based on cumulative safety data provided for their review.

15. ETHICS

15.1. Ethical Conduct of the Trial

The trial will be performed in accordance with the protocol, legal and regulatory requirements, and the general principals in the Declaration of Helsinki, International Ethical Guidelines for Biomedical Research Involving Human Subjects, and ICH Guidelines for GCP.

15.2. Written Informed Consent

A written informed consent form in compliance with 21 CFR Part 50 will be obtained from each subject prior to the subject entering the study or performance of any study specific procedures. The investigator at each center will ensure that the IRB approved consent form is used to provide the subject full and adequate oral and written information about the nature, purpose, possible risk, the possibility of disease progression or recurrence, and benefit of the trial. Subjects must also be notified that they are free to discontinue from the trial at any time. The subject should be given the opportunity to ask questions and allowed time to consider the information provided.

Remote/electronic consent procedures may be used following sponsor review of the process in the event of Covid-19 site impact.

The investigator must maintain the signed Informed Consent Form in the subject study record, along with documentation of the informed consent process (ie, statement of consent). A copy of the signed Informed Consent Form must be given to the subject. If the ICF is revised during the study, the subject must sign the revised consent form.

16. DATA HANDLING AND RECORDKEEPING

16.1. Retention of Records

The investigator must maintain all documentation relating to the trial (defined as all essential documents in ICH/GCP) for at least a period of 2 years after the last marketing application approval, or if not approved 2 years following the discontinuance of the study; or in accordance with local requirements (if longer). If it becomes necessary for Elicio or the Regulatory Authority to review any documentation relating to the trial, the investigator must permit access to such records.

The clinical site will provide details of storage location for all study related documentation to the sponsor or its designee both during and after the study.

17. PUBLICATION POLICY

Elicio encourages publication of results obtained during the conduct of the sponsored clinical trial, whether or not the results are positive or negative. To ensure against inadvertent disclosure of confidential information or unprotected inventions, the investigator and/or institution will provide Elicio the proposed publication at least thirty (30) days prior to disclosure so that Elicio has an opportunity to review prior to the submission or other disclosure. Upon the request of Elicio, Institution and Investigator will remove all confidential information of sponsor other than study data from such proposed publication. In addition, if any patent action is required to protect intellectual property rights or other proprietary rights contained in any proposed publication, the investigator and/or institution will delay the proposed publication for an additional 60 days to allow sponsor to obtain such protection.

If the site is part of a multicenter study, the first publication will be jointly authored by all clinical trial sites and any subsequent publications by the investigator will reference the first publication. Authorship will be determined by the sponsor and investigators according to the ethical standards concerning publication and authorship, including Section II – "Ethical considerations in the Conduct and Reporting of Research" of the requirements for submission to biomedical journals set by the International Committee of Medical Journal Editors. Subject identity will remain anonymous in any presentation or reporting of the trial. At least 2 sponsor representatives will also be included as co-authors on the first publication of the multicenter results to allow recognition of the sponsor's involvement in the design, clinical operations, and management of the study. If a joint manuscript is not submitted within 18 months of study completion the investigator is free to publish separately, following all other requirements in this section.

18. LIST OF REFERENCES

Aaronson NK, Ahmedzai S, Bergman B, et al. The European Organization for Research and Treatment of Cancer QLQ-C30: A quality-of-life instrument for use in international clinical trials in oncology. J Natl Cancer Inst. 1993; 85: 365-76.

Abbosh C, Birkbak NJ, Wilson GA. Phylogenetic ctDNA analysis depicts early-stage lung cancer evolution. Nature. 2017; 26;545(7655):446-451.

Abou-Alfa GK, Chapman PB, Feilchenfeldt J, et al. Targeting Mutated K-ras in Pancreatic Adenocarcinoma Using an Adjuvant Vaccine. Am J Clin Oncol. 2011;34: 321–325.

ACTEMRA® (tociluzumab) Prescribing Information. South San Francisco, California. Genentech, Inc. https://www.gene.com/download/pdf/actemra_prescribing.pdf. Accessed 23 Jan 2020.

Adam R, De Gramont A, Figueras J, et al. The oncosurgery approach to managing liver metastases from colorectal cancer: a multidisciplinary international consensus. Oncologist. 2012;17:1225-39.

Afrasanie VA, Marinca MV, Alexa-Stratulat T, et al. KRAS, NRAS, BRAF, HER2 and microsatellite instability in metastatic colorectal cancer – practical implications for the clinician. Radiol Oncol. 2019;53: 265-74.

Akgul O, Cetinkaya E, Ersoz S, Tez M. Role of surgery in colorectal cancer liver metastases. World J Gastroenterol. 2014;20: 6113-22.

Amri R, J. England L, Bordeianou G, Berger DL. Risk Stratification in Patients with Stage II Colon Cancer. Ann Surg Oncol. 2016;23: 3907-14.

Ansari D, Tingstedt B, Andersson B, et al. Pancreatic cancer: yesterday, today and tomorrow. Future Oncol. 2016;12(16):1929-46.

Antonia SJ, Vellegas A, Daniel D, et al. Durvalumab after chemoradiotherapy in Stage III non-small-cell lung cancer, N Engl J Med. 2017;377:1919-29.

Armstrong, DK. NCCN Guidelines in oncology: ovarian cancer including fallopian tube cancer and primary peritoneal cancer. https://www.nccn.org/professionals/physician_gls. Accessed 7 Jul 2020.

Auner V, Kriegshauser G, Tong D, et al. KRAS mutation analysis in ovarian samples using a high sensitivity biochip assay. BMC Cancer. 2009;9: 111.

Benson AB, Venook AP, Al-Hawary MM, et al. Rectal Cancer, Version 2.2018, NCCN Clinical Practice Guidelines in Oncology. J Natl Compr Canc Netw. 2018;16: 874-901.

Berger AC, Garcia M, Hoffman JP, et al. Postresection CA 19-9 predicts overall survival in patients with pancreatic cancer treated with adjuvant chemoradiation: a prospective validation by RTOG 9704. J Clin Oncol. 2008;26(36):5918-22.

Bettegowda C, Sausen M, Leary RJ, et al. Detection of circulating tumor DNA in early- and late-stage human malignancies. Sci Transl Med. 2014 Feb 19;6(224):224ra24.

Biankin AV, Waddell N, Kassahnet KS et al. Pancreatic cancer genomes reveal aberrations in axon guidance pathway genes. Nature. 2012;491(7424):399-405.

Blechacz B. Cholangiocarcinoma: Current Knowledge and New Developments. Gut Liver. 2017;11: 13-26.

Boeckx N, Peeters M, Van Camp G, et al. Prognostic and Predictive Value of RAS Gene Mutations in Colorectal Cancer: Moving Beyond KRAS Exon 2. Drugs. 2015;75: 1739-56.

Bonifant CL, Jackson HJ, Brentjens RJ, Curran KJ. Toxicity and management in CAR T-cell therapy. Mol Ther Oncolytics. 2016;20;3:16011.

Boussios S, Karihtala P, Moschetta M, et al. Combined Strategies with Poly (ADP-Ribose) Polymerase (PARP) Inhibitors for the Treatment of Ovarian Cancer: A Literature Review. Diagnostics (Basel). 2019;9.

Bray F, Ferlay J, Soerjomataram I, et al. Global cancer statistics 2018: GLOBOCAN estimates of incidence and mortality worldwide for 36 cancers in 185 countries. CA Cancer J Clin. 2018;68: 394-424.

Brentjens, RJ, Davila ML, Riviere I, et al. CD19-targeted T cells rapidly induce molecular remissions in adults with chemotherapy-refractory acute lymphoblastic leukemia. Sci Transl Med. 2012:177ra38.

Burdett SS, Stewart LA, Rydzewska L. Chemotherapy and surgery versus surgery alone in non-small cell lung cancer. Cochrane Database Syst Rev. 2007;(3):CD006157.

Cafri G, Gartner JJ, Zaks T, et al. mRNA vaccine-induced neoantigen-specific T cell immunity in patients with gastrointestinal cancer. J Clin Invest. 2020;130(11):5976-5988. doi:10.1172/JCI134915

Cancer Genome Atlas Research Network. Integrated genomic characterization of pancreatic ductal adenocarcinoma. Cancer Cell. 2017;32: 185-203 e13.

Canon J, Rex K, Saiki AY, et al. The clinical KRAS(G12c) inhibitor AMG 510 drives anti-tumour immunity. Nature. 2019; 575(7781):217-223.

Carvalho C, Glynne-Jones R. Challenges behind proving efficacy of adjuvant chemotherapy after preoperative chemoradiation for rectal cancer. Lancet Oncol. 2017;18: e354-e63.

Chatani PD, Yang JC. Mutated RAS: Targeting the “untargetable” with T cells. Clin Cancer Res. 2020;26: 537-44.

Chaudhuri AA, Chabon JJ, Lovejoy AF, et al. Early Detection of Molecular Residual Disease in Localized Lung Cancer by Circulating Tumor DNA Profiling. Cancer Discov. Dec 2017;7(12):1394-1403. doi:10.1158/2159-8290.CD-17-0716

Chawla A, Ferrone CR. Neoadjuvant therapy for resectable pancreatic cancer: an evolving paradigm shift. Front. Oncol. 2019;9:1085.

Chen F, Teachey DT, Pequignot E, et al. Measuring IL-6 and sIL-6R in serum from patients treated with tocilizumab and/or siltuximab following CAR T cell therapy. J Immunol Methods. 2016;434:1-8.

Churi CR, Shroff R, Wang Y, et al. Mutation profiling in cholangiocarcinoma: prognostic and therapeutic implications. PloS One. 2014;9: e115383.

Ciliberto D, Prati U, Roveda L, et al. Role of systemic chemotherapy in the management of resected or resectable colorectal liver metastases: a systematic review and meta-analysis of randomized controlled trials. Oncol Rep. 2012;27: 1849-56.

Clinical Trial Endpoints for the Approval of Cancer Drugs and Biologics. Guidance for Industry. U.S. Department of Health and Human Services Food and Drug Administration Oncology Center of Excellence Center for Drug Evaluation and Research (CDER) Center for Biologics Evaluation and Research (CBER), December 2018. https://www.fda.gov/media/71195/download Accessed 5 Jan 2020.

Conroy T, Hammel P, Hebbar M, et al. FOLFIRINOX or gemcitabine as adjuvant therapy for pancreatic cancer. N Engl J Med. 2018;379(25):2395-2406.

Cooper CL, Davis HL, Morris ML, et al. CPG 7909, an immunostimulatory TLR-9 agonist oligodeoxynucleotide, as adjuvant to Engerix-B HBV vaccine in healthy adults: a double-blind phase I/II study. J Clin Immunol. 2004;24: 693-701.

Diaz LA, Bardelli A. Liquid biopsies: genotyping circulating tumor DNA. J Clin Oncol. 2014;32(6): 579–586.

Diehl F, Schmidt K, Choti MA, et al. Circulating mutant DNA to assess tumor dynamics. Nat Med. 2008;14: 985-90.

Dobre M, Dinu DE, Panaitescu E, et al. KRAS gene mutations – prognostic factor in colorectal cancer? Rom J Morphol Embryol. 2015;56: 671-8.

Dobrzycka B, Terlikowski SJ, Kinalski M, et al. Circulating free DNA and p53 antibodies in plasma of patients with ovarian epithelial cancers. Ann Oncol. 2011;22: 1133-40.

Dogan S, Shen R, Ang DC, et al. Molecular epidemiology of EGFR and KRAS mutations in 3,026 lung adenocarcinomas: higher susceptibility of women to smoking-related KRAS-mutant cancers. Clin Cancer Res. 2012;18:6169-77.

Douillard JY, Oliner KS, Siena S, et al. Panitumumab-FOLFOX4 treatment and RAS mutations in colorectal cancer. N Engl J Med. 2013;369: 1023-34.

Ericksen JA, Gladhaug I, Rosseland A. An Observational Clinical Study with RAS Peptide Vaccine TG01 Evaluating Immune Response, Safety and Overall Survival in Patients with Non-Resectable Pancreatic Cancer. ESMO Congress. Madrid, Spain, Sep 2017. Poster 2.

Eser S, Schnieke A, Cchneider G, Saur D. Oncogenic KRAS signaling in pancreatic cancer. Br J Cancer. 2014;111:817-822.

Ettrich TJ, Schwerdel D, Dolnik A, et al. Genotyping of circulating tumor DNA in cholangiocarcinoma reveals diagnostic and prognostic information. Sci Rep. 2019;9: 13261.

Fan G, Zhang K, Yang X, et al. Prognostic value of circulating tumor DNA in patients with colon cancer: Systematic review. PloS One. 2017;12: e0171991.

Felip E, Altorki N, Zhou C, et al. Adjuvant atezolizumab after adjuvant chemotherapy in resected stage IB-IIIA non-small-cell lung cancer (IMpower010): a randomised, multicentre, open-label, phase 3 trial. Lancet. 2021;398(10308):1344-1357. doi:10.1016/S0140-6736(21)02098-5

FDA Press Release: FDA approves neoadjuvant nivolumab and platinum-doublet chemotherapy for early-stage non-small cell lung cancer. March 4, 2022. Available at: https://www.fda.gov/drugs/resources-information-approved-drugs/fda-approves-neoadjuvant-nivolumab-and-platinum-doublet-chemotherapy-early-stage-non-small-cell-lung#:~:text=On%20March%204%2C%202022%2C%20the,NSCLC)%20in%20the%20neoadjuvant%20setting. Accessed April 2, 2022.

Food and Drug Administration Guidance on Conduct of Clinical Trials of Medicinal Products during COVID-19 Public Health Emergency, March 2020 and April 2020 update. https://www.fda.gov/regulatory-information/search-fda-guidance-documents/fda-guidance-conduct-clinical-trials-medical-products-during-covid-19-public-health-emergency; https://www.hhs.gov/ohrp/sites/default/files/fda-covid-guidance-2apr2020.pdf. Accessed 26Jun2020.

Gedde-Dahl T, Nilsen E, Thorsby E, Gaudernack G. Growth inhibition of a colonic adenocarcinoma cell line (HT29) by T cells specific for mutant p21 ras', Cancer Immunol Immunother. 1994; 38: 127-34.

Giannopoulou L, Kasimir-Bauer S, Lianidou SE. Liquid biopsy in ovarian cancer: recent advances on circulating tumor cells and circulating tumor DNA. Clin Chem Lab Med. 2018;56: 186-97.

Giavridis T, van der Stegen SJC, Eyquem J, et al. CAR T cell-induced cytokine release syndrome is mediated by macrophages and abated by IL-1 blockade. Nat Med. 2018;24(6):731-738.

Gjertsen MK, Buanes T. Rosseland AR. Intradermal RAS peptide vaccination with granulocyte-macrophage colony-stimulating factor as adjuvant: clinical and immunological responses in patients with pancreatic adenocarcinoma. Int. J. Cancer. 2001:92: 441–450.

Gjertsen MK, Bakka A, Breivik J, et al. *Ex vivo* ras peptide vaccination in patients with advanced pancreatic cancer: Results of a Phase I/II study. Int J Cancer. 1996;65(4):450-453.

Gong J, Cho M, Sy M, et al. Molecular profiling of metastatic colorectal tumors using next-generation sequencing: a single-institution experience. Oncotarget. 2017;8: 42198-213.

Groot VP, Mosier S, Javed AA, et al. Circulating tumor DNA as a clinical test in resected pancreatic cancer. Clin Cancer Res. 2019;25:4973–84.

Grothey A, Sobrero AF, Shields AF, et al. Duration of adjuvant chemotherapy for Stage III colon cancer. N Engl J Med. 2018;378: 1177-88.

Grupp SA, Kalos M, Barrett D, et al. Chimeric antigen receptor-modified T cells for acute lymphoid leukemia. N Engl J Med. 2013;368(16):1509-1518.

Guo, S, Xiaohan S, Jing S, et al. Preoperative detection of KRAS G12D mutation in ctDNA is a powerful predictor for early recurrence of resectable PDAC patients. British Journal of Cancer. 2020;122: 857-67.

Haas M, Ormanns S, Baechmannet S, et al. Extended RAS analysis and correlation with overall survival in advanced pancreatic cancer. Br J Cancer. 2017;116:1462-69.

Heinemann V, Stintzing S, Kirchner T, et al. Clinical relevance of EGFR- and KRAS-status in colorectal cancer patients treated with monoclonal antibodies directed against the EGFR. Cancer Treat Rev. 2009;35: 262-71.

Hendifar AE, Blais EM, Ng C, et al. Comprehensive analysis of KRAS varians in patients with pancreatic cancer (PDAC): Clinical/molecular correlations and real-world outcomes across standard therapies. J Clin Oncol. 2020;38 suppl; abstr 4641.

Hong DS, Fakih MG, Strickler JH, et al. KRASG12C Inhibition with Sotorasib in Advanced Solid Tumors. N Engl J Med 2020; 383:1207-1217

Illei PB, Belchis D, Tseng LH, et al., Clinical mutational profiling of 1006 lung cancers by next generation sequencing. Oncotarget. 2017;8:96684-96.

Imaoka H, Shimizu Y, Senda Y, et al. Post-adjuvant chemotherapy CA19-9 levels predict prognosis in patients with pancreatic ductal adenocarcinoma: A retrospective cohort study. Pancreatology. 2016;16(4):658-64.

Jensen LH, R Andersen RF, Byriel L, et al. Phase II study of gemcitabine, oxaliplatin and capecitabine in patients with KRAS exon 2 mutated biliary tract cancers. Acta Oncol. 2020;59:298-301.

Kaasa S, Bjordal K, Aaronson N, et al. The EORTC core quality of life questionnaire (QLQ-C30): validity and reliability when analysed with patients treated with palliative radiotherapy. Eur J Cancer. 1995; 31A: 2260-3.

Kasi PM, Dayyani F, Morris VK, et al. Tumor-informed assessment of molecular residual disease and its incorporation into practice for patients with early and advanced stage colorectal cancer (CRC-MRD Consortia). J Clin Oncol. 2020;38: 2020 (suppl; abstr 4108).

Kato Y, Takahashi S, Gotohda N, Konishi M. Prognostic Impact of the Initial Postoperative CA19-9 Level in Patients with Extrahepatic Bile Duct Cancer. J Gastrointest Surg. 2016;20(8):1435-43.

Khleif SN, Abrams SI, Hamilton JM, et al. A phase I vaccine trial with peptides reflecting ras oncogene mutations of solid tumors. J Immunother. 1999;22: 155-65.

Kim SW, Her KH, Jang JY, et al. K-ras oncogene mutation in cancer and precancerous lesions of the gallbladder. J Surg Oncol. 2000;75: 246-51.

Kim YM, Lee SW, Lee YJ, et al. Prospective study of the efficacy and utility of TP53 mutations in circulating tumor DNA as a non-invasive biomarker of treatment response monitoring in patients with high-grade serous ovarian carcinoma. J Gynecol Oncol. 2019;30: e32.

Kirstein MM, Vogel A. Epidemiology and risk factors of cholangiocarcinoma. Visc Med. 2016;32: 395-400.

Kondo N, Murakami Y, Uemura K, et al. Elevated perioperative serum CA 19-9 levels are independent predictors of poor survival in patients with resectable cholangiocarcinoma. J Surg Oncol. 2014;110(4):422-9.

Krell RW, D'Angelica MI. Treatment sequencing for simultaneous colorectal liver metastases. J Surg Oncol. 2019;119: 583-93.

Krieg AM, Efler SM, Wittpoth M, et al. Induction of systemic TH1-like innate immunity in normal volunteers following subcutaneous but not intravenous administration of CPG 7909, a synthetic B-class CpG oligodeoxynucleotide TLR-9 agonist. J Immunother. 2004;27: 460-71.

Kudrin A. Overview of cancer vaccines. Hum Vaccin Immunother. 2012;8(9):1335–1353.

Lee DW, Kochenderfer JN, Stetler-Stevenson M, et al. T cells expressing CD19 chimeric antigen receptors for acute lymphoblastic leukaemia in children and young adults: A Phase 1 dose-escalation trial. www.thelancet.com Published online October 13, 2014. http://dx.doi.org/10.1016/S0140-6736(14)61403-3. Accessed 3 Jan 2020.

Lee DW, Kochenderfer JN, Stetler-Stevenson M, et al. T cells expressing CD19 chimeric antigen receptors for acute lymphoblastic leukaemia in children and young adults: a phase 1 dose-escalation trial. Lancet. 2015;385(9967):517-528.

Lee DW, Santomasso BD, Locke FL, et al. ASTCT consensus grading for cytokine release syndrome and neurologic toxicity associated with immune effector cells. Biol Blood Marrow Transplant. 2019;25(4):625-638.

Lee B, Lipton L, Cohen J, et al. Circulating tumor DNA as a potential marker of adjuvant chemotherapy benefit following surgery for localized pancreatic cancer. Ann Oncol. 2019;30(9):1472-1478.

Liang WS, Craig DW, Carpten J, et al. Genome-wide characterization of pancreatic adenocarcinoma patients using next generation sequencing. PLoS One. 2012;7(10):e43192.

Litvak A, Cercek A, Segal N, et al. False-positive elevations of carcinoembryonic antigen in patients with a history of resected colorectal cancer. J Natl Compr Canc Netw. 2014;12(6):907-13.

Liu H, Moynihan KD, Zheng Y, et al. Structure-based programming of lymph-node targeting in molecular vaccines. Nature. 2014;507(7493):519-22.

Ma L, Dichwalkar T, Chang JYH. Enhanced CAR-T Cell Activity Against Solid Tumors by Vaccine Boosting Through the Chimeric Receptor. Science. 2019;365(6449):162-468.

Macdonald S, Mair F. Tackling cancers of unmet need: the pancreatic cancer pathway. Lancet. 2016;1(4):266-267.

Malvezzi M, Carioli G, Bertuccio P. European cancer mortality predictions for the year 2018 with focus on colorectal cancer. Ann Oncol. 2018;29(4):1016-1022.

Maude SL, Barrett D, Teachey DT, Grupp SA. Managing cytokine release syndrome associated with novel T cell-engaging therapies. Cancer J. 2014;20(2):119-22.

Maude SL, Teachey DT, Porter DL, Grupp SA. CD19-targeted chimeric antigen receptor T-cell therapy for acute lymphoblastic leukemia. Blood. 2015;125: 4017-23.

Melisi D, Frizziero M, Tamburrino A, et al. Toll-like Receptor 9 agonists for cancer therapy. Biomedicines. 2014;2:211-228.

Mestermann K, Giavridis T, Weber J, et al. The tyrosine kinase inhibitor dasatinib acts as a pharmacologic on/off switch for CAR T cells. Science Translational Medicine. 2019;11(499): eaau5907.

Miller KD, Nogueira L, Mariotto AB, et al. Cancer treatment and survivorship statistics, 2019. CA Cancer J Clin. 2019;69: 363-85.

Modest DP, Ricard I, Heinemann V, et al. Outcome according to KRAS-, NRAS- and BRAF-mutation as well as KRAS mutation variants: pooled analysis of five randomized trials in metastatic colorectal cancer by the AIO colorectal cancer study group. Ann Oncol. 2016;27: 1746-53.

Morikawa A, Hayashi T, Shimizu N, et al. PIK3CA and KRAS mutations in cell free circulating DNA are useful markers for monitoring ovarian clear cell carcinoma. Oncotarget. 2018;9: 15266-74.

Moynihan KD, Opel CF, Szeto GL et al. Eradication of large established tumors in mice by combination immunotherapy that engages innate and adaptive immune responses. Nat Med. 2016;22(12):1402-10.

Moynihan KD, Holden RL, Mehta NK, et al. Enhancement of Peptide Vaccine Immunogenicity by Increasing Lymphatic Drainage and Boosting Serum Stability. 2018. DOI: 10.1158/2326-6066.CIR-17-0607.

Nabet BY, Esfahani MS, Moding EJ, et al. Noninvasive Early Identification of Therapeutic Benefit from Immune Checkpoint Inhibition. Cell. 2020;183(2):363-376 e13. doi:10.1016/j.cell.2020.09.001

National Cancer Institute: Surveillance, Epidemiology, and End Results Program (SEER) https://seer.cancer.gov/statfacts/html/pancreas.html / accessed 3 Jan 2020.

National Comprehensive Cancer Network. Hepatobiliary Cancers (Version 5.2021 – September 21, 2021). https://www.nccn.org/professionals/physician_gls/pdf/hepatobiliary.pdf. Accessed March 2, 2022.

National Comprehensive Cancer Network. Ovarian Cancer Including Fallopian Tube Cancer and Primary Peritoneal Cancer (Version 1.2022 – January 18, 2022). https://www.nccn.org/professionals/physician_gls/pdf/ovarian.pdf. Accessed March 2, 2022.

National Comprehensive Cancer Network. Pancreatic Adenocarcinoma (Version 1.2022 – February 24, 2022). https://www.nccn.org/professionals/physician_gls/pdf/pancreatic.pdf. Accessed March 2, 2022.

National Comprehensive Cancer Network. Rectal Cancer (Version 1.2022 – February 25, 2022). https://www.nccn.org/professionals/physician_gls/pdf/rectal.pdf. Accessed March 2, 2022.

Neelapu SS, Tummala S, Kebriaei P, et al. Chimeric antigen receptor T-cell therapy - assessment and management of toxicities. Nat Rev Clin Oncol. 2018;15(1):47-62.

Oettle H, Post S, Neuhaus, et al. Adjuvant chemotherapy with gemcitabine vs observation in patients undergoing curative-intent resection of pancreatic cancer. a randomized controlled trial. JAMA. 2007;297(3):267-277.

Osoba D, Pater JL, Zee B. Effective anti-emetic therapy improves quality of life (QoL) after moderately emetogenic chemotherapy (MEC). Qual Life Res. 1994;4:467-8.

Palmer D, Valle, J, et al. TG01/GM-CSF and adjuvant gemcitabine in patients with resected RAS-mutant adenocarcinoma of the pancreas (CT TG01-01): a single-arm, phase 1/2 trial. Br J Cancer. 2020;122:971-977.

Park JH, Rivière I, Gonen M, et al. Long-Term Follow-up of CD19 CAR Therapy in Acute Lymphoblastic Leukemia. N Engl J Med. 2018;378:449-459. DOI: 10.1056/NEJMoa1709919.

Perets R, Greenberg O, Shentzer T, et al. Mutant KRAS circulating tumor DNA is an accurate tool for pancreatic cancer monitoring. Oncologist. 2018;23(5):566-572.

Perren, TJ. Mucinous epithelial ovarian carcinoma. Ann Oncol. 2016;27(Suppl 1): i53-i57.

Philip PA, Lacy J, Portales F, et al. Nab-paclitaxel plus gemcitabine in patients with locally advanced pancreatic cancer (LAPACT): a multicentre, open-label phase 2 study. Lancet Gastroenterol Hepatol. 2020 Mar;5(3):285-294.

Pignon JP, Tribodet H, Scagliotti GV, et al. Lung adjuvant cisplatin evaluation: a pooled analysis by the LACE Collaborative Group. J Clin Oncol. 2008;26:3552-9.

PORT Meta-analysis Trialists Group. Postoperative radiotherapy for non-small cell lung cancer. Cochrane Database Syst Rev. 2005;2:CD002142.

Prat J, D'Angelo E, Espinosa I. Ovarian carcinomas: at least five different diseases with distinct histological features and molecular genetics. Hum Pathol. 2018;80: 11-27.

Price TJ, Piantadosi C, Townsend AR, et al. Prognostic differences of RAS mutations: Results from South Australian metastatic colorectal registry. J Clin Oncol 2020;38: 2020 (suppl; abstr 4108).

Primrose JN, Fox RP, Palmer DH, et al. Capecitabine compared with observation in resected biliary tract cancer (BILCAP): a randomised, controlled, multicentre, phase 3 study. Lancet Oncol. 2019;20: 663-73

Prior IA, Lewis PD, Mattos C. A comprehensive survey of Ras mutations in cancer. Cancer Res. 2012;72: 2457-67.

PROVENGE® (sipuleucel-T) Prescribing Information. Seattle, Washington. Dendreon Corporation. https://www.provenge.com/Why-Immunotherapy. Accessed 30 Jul 2019.

Rahma OE, Hamilton JM, Wojtowicz M, et al. The immunological and clinical effects of mutated ras peptide vaccine in combination with IL-2, GM-CSF, or both in patients with solid tumors. J Transl Med. 2014;12: 55.

Raje N, Berdeja J, Lin Y, et al. Anti-BCMA CAR T-Cell therapy bb2121 in relapsed or refractory multiple myeloma. N Engl J Med. 2019;380(18):1726-1737.

Rambau PF, McIntyre JB, Taylor J, et al. Morphologic reproducibility, genotyping, and immunohistochemical profiling do not support a category of seromucinous carcinoma of the ovary. Am J Surg Pathol. 2017;41: 685-95.

Ravdin PM, Davis G. Prognosis of patients with resected non-small cell lung cancer: impact of clinical and pathologic variables. Lung Cancer. 2006;52:207

Rawla, P, Sunkara T, Thandra KC, Barsouk A. Epidemiology of gallbladder cancer. Clin Exp Hepatol. 2019;5: 93-102.

Rechsteiner M, Zimmermann AK, Wild PJ, et al. TP53 mutations are common in all subtypes of epithelial ovarian cancer and occur concomitantly with KRAS mutations in the mucinous type. Exp Mol Pathol. 2013;95: 235-41.

Reinert T, Scholer LV, Thomsen R, et al. Analysis of circulating tumour DNA to monitor disease burden following colorectal cancer surgery. Gut. 2016;65: 625-34.

Reinert T, Henriksen TV, Christensen E. Analysis of plasma cell-free DNA by ultradeep sequencing in patients with Stages I to III colorectal cancer. JAMA Oncol. 2019; doi: 10.1001/jamaoncol.2019.0528.

Rieser CJ, Zenati M, Hamad A, et al. CA19-9 on Postoperative Surveillance in Pancreatic Ductal Adenocarcinoma: Predicting Recurrence and Changing Prognosis over Time. Ann Surg Oncol. 2018;25(12):3483-3491.

Rizvi S, Khan SA, Hallemeier CL, et al. Cholangiocarcinoma - evolving concepts and therapeutic strategies. Nat Rev Clin Oncol. 2018;15: 95-111.

Sausen M, Phallen J, Adleff V, et al. Clinical implications of genomic alterations in the tumour and circulation of pancreatic cancer patients. Nat Commun. 2015;6:7686.

Sener SF, Fremgen A, Menck HR, et al. Pancreatic cancer: a report of treatment and survival trends for 100,313 patients diagnosed from 1985–1995, using the National Cancer Database. JACS. 1999;189(1):1-7.

Sethi H, Salari R, Navarro S, et al, 2018. Analytical validation of the Signatura™ RUO assay, a highly sensitive patient-specific multiplex PCR NGS-based non-invasive cancer recurrence detection and therapy monitoring assay. Cancer Research. 2018;78(13):4542.

Seymour L, Bogaerts J, Perrone A, et al. iRECIST: guidelines for response criteria for use in trials testing immunotherapeutics. Lancet Oncol. 2017;18(3):e143-e152.

Shanmugam V, Ramanathan RK, Lavender NA, et al. Whole genome sequencing reveals potential targets for therapy in patients with refractory KRAS mutated colorectal cancer. BMC Med Genomics. 2014;7:36.

Shono Y, Tanimura H, Iwahashi M, et al. Specific T-cell immunity against Ki-ras peptides in patients with pancreatic and colorectal cancers. Br J Cancer. 2003;88: 530-6.

Shroff RT, Kennedy EB, Bachini M, et al. Adjuvant therapy for resected biliary tract cancer: ASCO Clinical Practice Guideline. J Clin Oncol. 2019;37: 1015-27.

Siegel RL, Miller KD, Fuchs HE, Jemal A. Cancer Statistics, 2021, CA Cancer J Clin. 2021;71:7-33.

Siegel RL, Miller KD, Jemal A. Cancer statistics, 2020. CA Cancer J Clin. 2020;70:7-30.

Simbolo M, Fassan M, Ruzzenente A, et al. Multigene mutational profiling of cholangiocarcinomas identifies actionable molecular subgroups. Oncotarget. 2014;5: 2839-52.

Singhi AD, George B, Greenbowe JR, et al. Real-time targeted genome profile analysis of pancreatic ductal adenocarcinomas identifies genetic alterations that might be targeted with existing drugs or used as biomarkers. Gastroenterology. 2019;156:2242-53.

Singh N, Gupta S, Pandey RM, Chauhan SS, Saraya A. High levels of cell-free circulating nucleic acids in pancreatic cancer are associated with vascular encasement, metastasis and poor survival. Cancer Invest. 2015;33:78-85.

Singh N, June CH. Boosting engineered T cells. Science. 2019;365(6449):119-120.

Strauss GM, Herndon JE, Maddaus MA, et al. Adjuvant paclitaxel plus carboplatin compared with observation in stage IB non-small-cell lung cancer: CALGB9633 with the Cancer and Leukemia Group B, Radiation Therapy Oncology Group, and North Central Cancer Treatment Group Study Groups. J Clin Oncol. 2008;26:5043-51.

Summers MG, Smith CG, Maughan TS, et al. BRAF and NRAS locus-specific variants have different outcomes on survival to colorectal cancer. Clin Cancer Res. 2017;23: 2742-49.

Takada T, Amano H, Yasuda H, et al. Study Group of Surgical Adjuvant Therapy for Carcinomas of the Pancreas and Biliary Tract. Is postoperative adjuvant chemotherapy useful for gallbladder carcinoma? A phase III multicenter prospective randomized controlled trial in patients with resected pancreaticobiliary carcinoma. Cancer. 2002 Oct 15;95(8):1685-95.

Tarazona N, Henricksen TV, Carbonell-Asins JA, et al. Circulating tumor DNA to detect minimal residual disease, response to adjuvant therapy and identify patients at high risk of recurrence in patients with Stage I-III CRC. J Clin Oncol. 2020;38: 2020 (suppl; abstr 4009).

Teachey DT, Lacey SF, Shaw PA, et al. Identification of predictive biomarkers for cytokine release syndrome after chimeric antigen receptor T-cell therapy for acute lymphoblastic leukemia. Cancer Discov. 2016;6: 664-79.

Tie J, Cohen JD, Wang Y, et al. Circulating Tumor DNA Analyses as Markers of Recurrence Risk and Benefit of Adjuvant Therapy for Stage III Colon Cancer. JAMA Oncol. 2019 Dec 1;5(12):1710-1717.

Tie J, Wang Y, Tomasetti C, et al. Circulating tumor DNA analysis detects minimal residual disease and predicts recurrence in patients with stage II colon cancer. Sci Transl Med. 2016 Jul 6;8(346):346ra92.

Toubaji A, Achtar M, Provenzano M, et al. Pilot study of mutant ras peptide-based vaccine as an adjuvant treatment in pancreatic and colorectal cancers. Cancer Immunol Immunother. 2008;57: 1413-20.

Tran E, Robbins PF, Lu YC, et al. T-cell transfer therapy targeting mutant KRAS in cancer. N Engl J Med. 2016;375: 2255-62.

Valle J, Wasan H, Palmer DH, et al. Cisplatin plus gemcitabine versus gemcitabine for biliary tract cancer. N Engl J Med. 2010;362: 1273-81.

Van der Burg MEL, Lammes FB, Verweij J. The role of CA 125 in the early diagnosis of progressive disease in ovarian cancer. Annals of Oncology. 1990;1:301-302.

Verberne CJ, Zhan Z, van den Heuvel E, et al. Intensified follow-up in colorectal cancer patients using frequent Carcino-Embryonic Antigen (CEA) measurements and CEA-triggered imaging: Results of the randomized "CEAwatch" trial. Eur J Surg Oncol. 2015;41(9):1188-96.

Verberne CJ, Zhan Z, van den Heuvel E, et al. Survival analysis of the CEAwatch multicentre clustered randomized trial. Br J Surg. 2017;104(8):1069-77.

Vigil D, Cherfils J, Rossman KL, Der CJ. Ras superfamily GEFs and GAPS: Validated and tractable targets for cancer therapy? Nat Rev Cancer. 2010;10:842-857.

Vymetalkova V, Cervena K, Bartu L, Vodicka P. Circulating cell-free DNA and colorectal cancer: a systematic review. Int J Mol Sci. 2018;19.

Wang QJ, Yu Z, Griffith K, et al. Identification of T-cell receptors targeting KRAS-mutated human tumors. Cancer Immunol Res. 2016;4: 204-14.

Waters AM, Der CJ. KRAS: The critical driver and therapeutic target for pancreatic cancer. Cold Spring Harb Perspect Med. 2018; 8:1-22.

Weber JS, Hodi FS, Wolchok JD, et al. Safety profile of nivolumab monotherapy: a pooled analysis of patients with advanced melanoma. J Clin Oncol. 2017;35(7):785-792.

Wedén S, Klemp M, Gladhaug IP, et al. Long-term follow-up of patients with resected pancreatic cancer following vaccination against mutant K-ras. Int J Cancer. 2011;128(5):1120-1128.

Wolchok JD, Hoos A, O'Day S, et al. Guidelines for the evaluation of immune therapy activity in solid tumors: immune-related response criteria. Clin Cancer Res. 2009;15:7412-7420.

Yang A, Farmer E, Wu T-C, Hung C-F. Perspectives for therapeutic HPV vaccine development. J Biomed Sci. 2016a;23(75).

Yang A, Jeang J, Cheng K, et al. Current state in the development of candidate therapeutic HPV vaccines. Expert Rev Vaccines. 2016b;15(8):989–1007. doi:10.1586/14760584.2016b.1157477.

Yang A, Farmer E, Lin J, et al. The current state of therapeutic and T cell-based vaccines against human papillomaviruses. Virus Res. 2017;231:148–165. doi:10.1016/j.virusres.2016.12.002.

Yao KJ, Jabbour S, Parekh N, et al. Increasing mortality in the United States from cholangiocarcinoma: an analysis of the National Center for Health Statistics Database. BMC Gastroenterol. 2016;16: 117.

Zent CS, Smith BJ, Ballas ZK, et al. Phase I clinical trial of CpG oligonucleotide 7909 (PF-03512676) in patients with previously treated chronic lymphocytic leukemia. Leuk Lymphoma. 2012;53: 211-7

Zhang J, Zheng J, Yang Y, et al. Molecular spectrum of KRAS, NRAS, BRAF and PIK3CA mutations in Chinese colorectal cancer patients: analysis of 1,110 cases. Sci Rep. 2015;5: 18678.

Zou S, Li J, Zhou H, et al. Mutational landscape of intrahepatic cholangiocarcinoma. Nat Commun. 2014;5: 5696.

19. APPENDICES

19.1. ECOG Performance Status Scale

The ECOG Performance Status Scale is provided in Table 24.

Table 24: ECOG Performance Status Scale

GRADE	ECOG PERFORMANCE STATUS
0	Normal activity. Fully active, able to carry on all pre-disease performance without restriction.
1	Symptoms, but ambulatory. Restricted in physically strenuous activity, but ambulatory and able to carry out work of a light or sedentary nature (eg, light housework, office work).
2	In bed <50% of the time. Ambulatory and capable of all self-care, but unable to carry out any work activities. Up and about more than 50% of waking hours.
3	In bed >50% of the time. Capable of only limited self-care, confined to bed or chair more than 50% of waking hours.
4	100% bedridden. Completely disabled. Cannot carry on any self-care. Totally confined to bed or chair.
5	Dead

CRS/ICANS Consensus Criteria. Kasi PM, Dayyani F, MorrisVK, et al. Tumor-Informed Assessment of Molecular Residual Disease and its Incorporation into Pratice for Patients with Early and Advanced Stage Colorectal Cancer (CRC-MRD Consortia). J Clin Oncol. 2020;38:(suppl; abstr 4108).
